# Supplementary figures and images for: Screening of prognostic biomarkers for endometrial carcinoma based on a ceRNA network (part 1 of 2)
Source: PeerJ. 2018 Dec 10;6:e6091. doi: 10.7717/peerj.6091 (PMC6292375; doi:10.7717/peerj.6091)

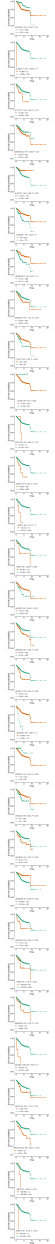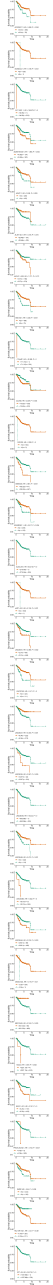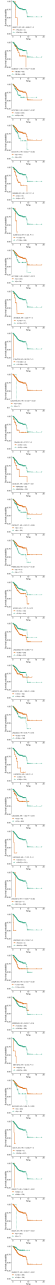

Supplement: Table S9 [file peerj-06-6091-s009.zip › Table S9/bestSep_good.pdf]

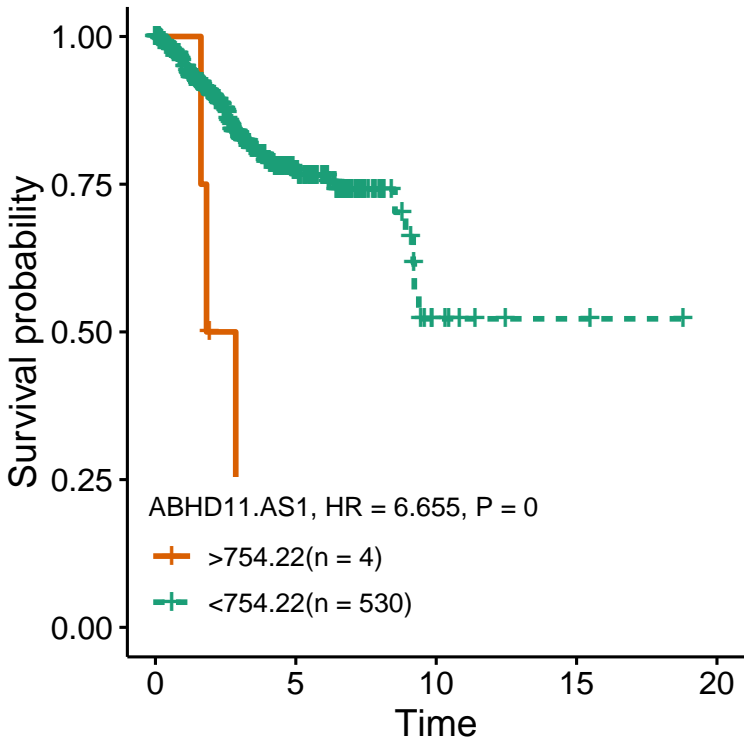

Supplement: Table S9 [file peerj-06-6091-s009.zip › Table S9/good_ABHD11.AS1.pdf]

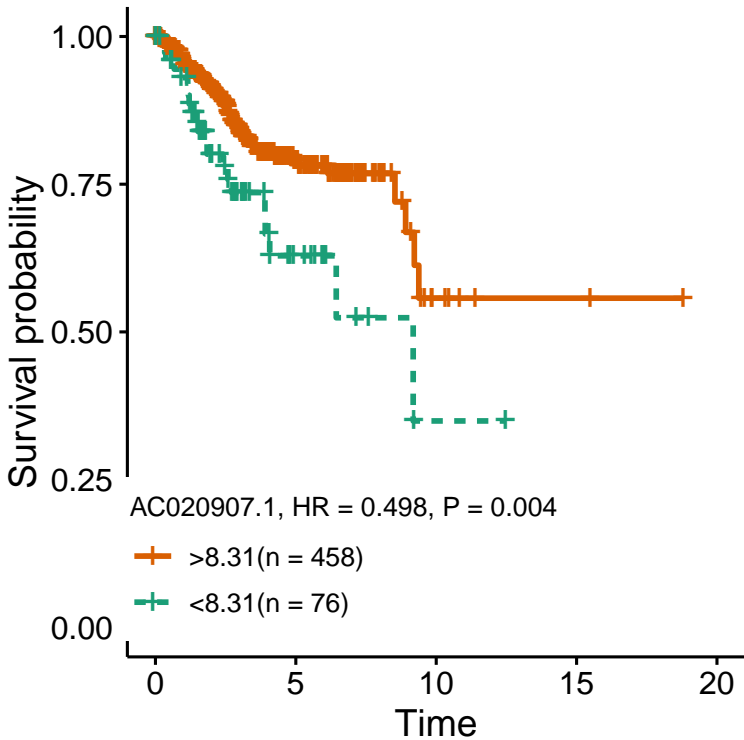

Supplement: Table S9 [file peerj-06-6091-s009.zip › Table S9/good_AC020907.1.pdf]

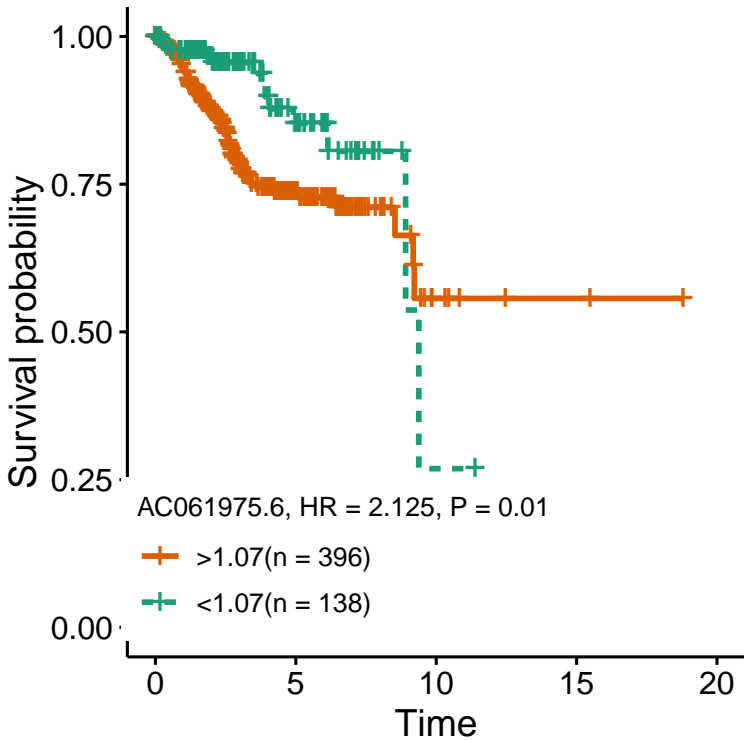

Supplement: Table S9 [file peerj-06-6091-s009.zip › Table S9/good_AC061975.6.pdf]

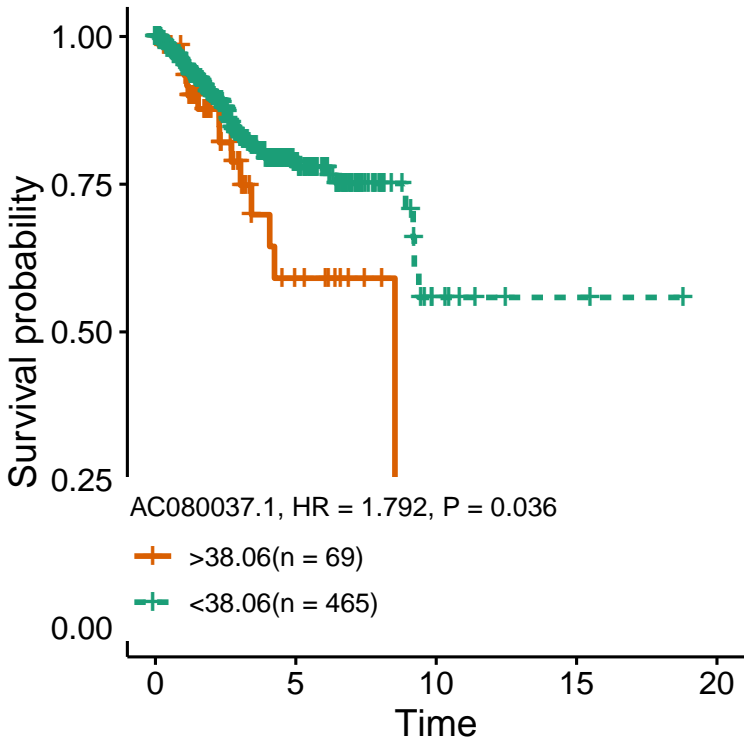

Supplement: Table S9 [file peerj-06-6091-s009.zip › Table S9/good_AC080037.1.pdf]

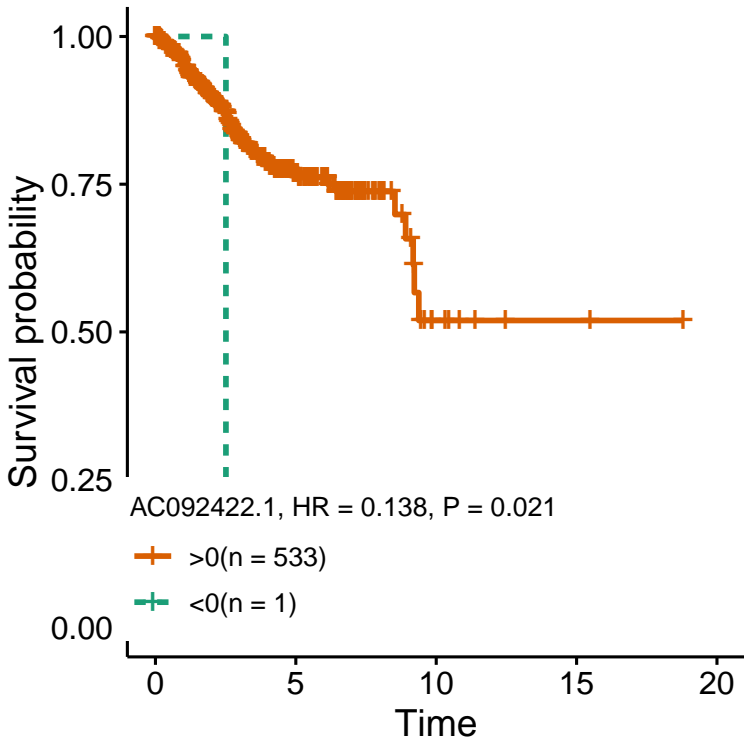

Supplement: Table S9 [file peerj-06-6091-s009.zip › Table S9/good_AC092422.1.pdf]

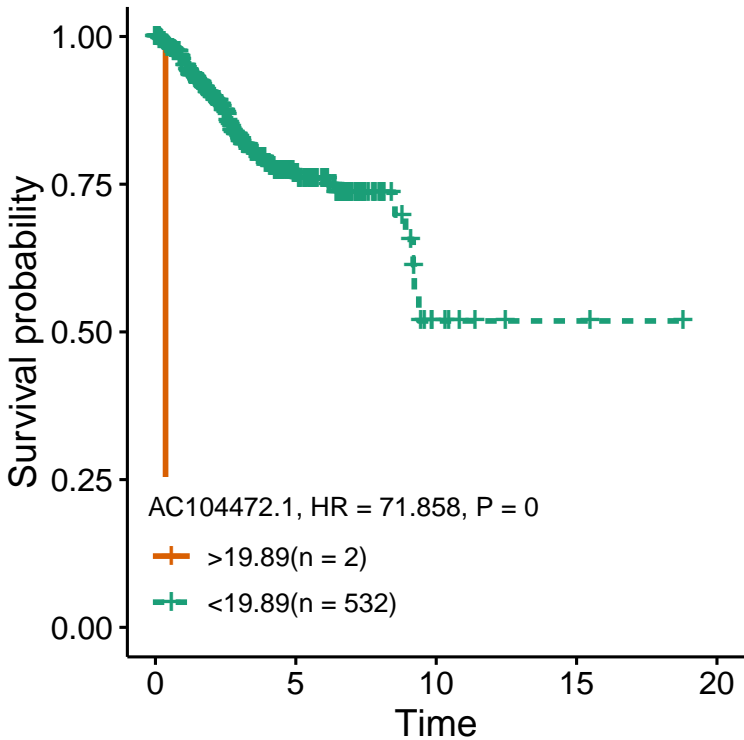

Supplement: Table S9 [file peerj-06-6091-s009.zip › Table S9/good_AC104472.1.pdf]

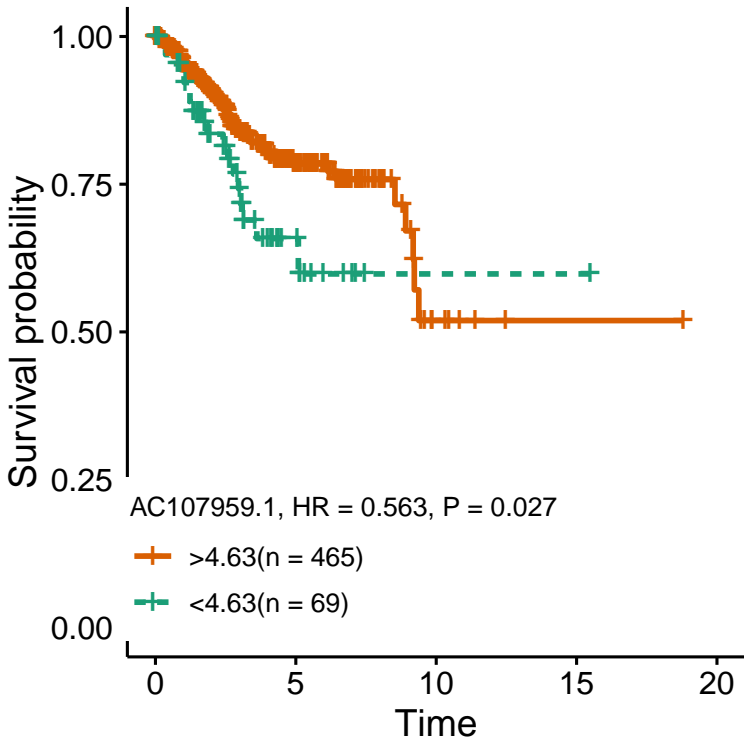

Supplement: Table S9 [file peerj-06-6091-s009.zip › Table S9/good_AC107959.1.pdf]

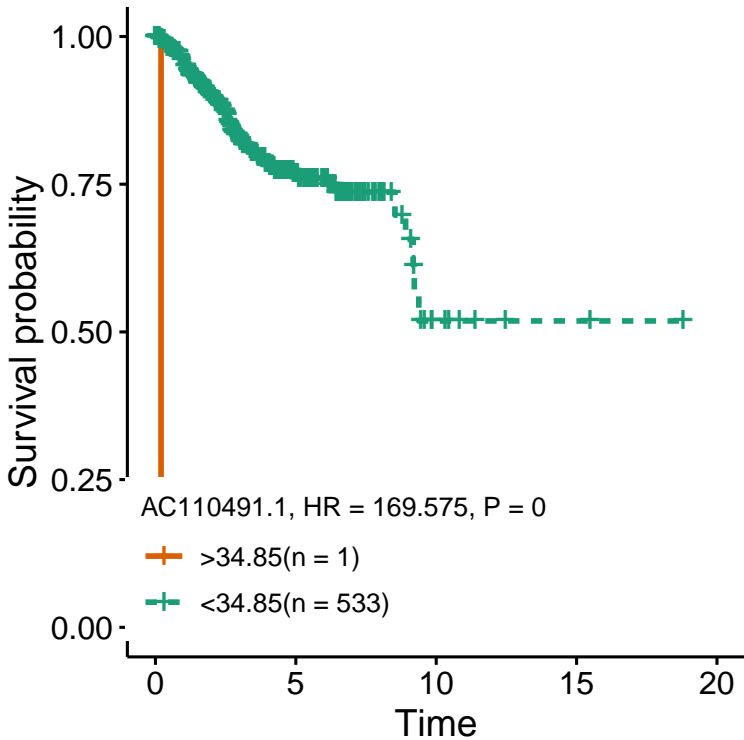

Supplement: Table S9 [file peerj-06-6091-s009.zip › Table S9/good_AC110491.1.pdf]

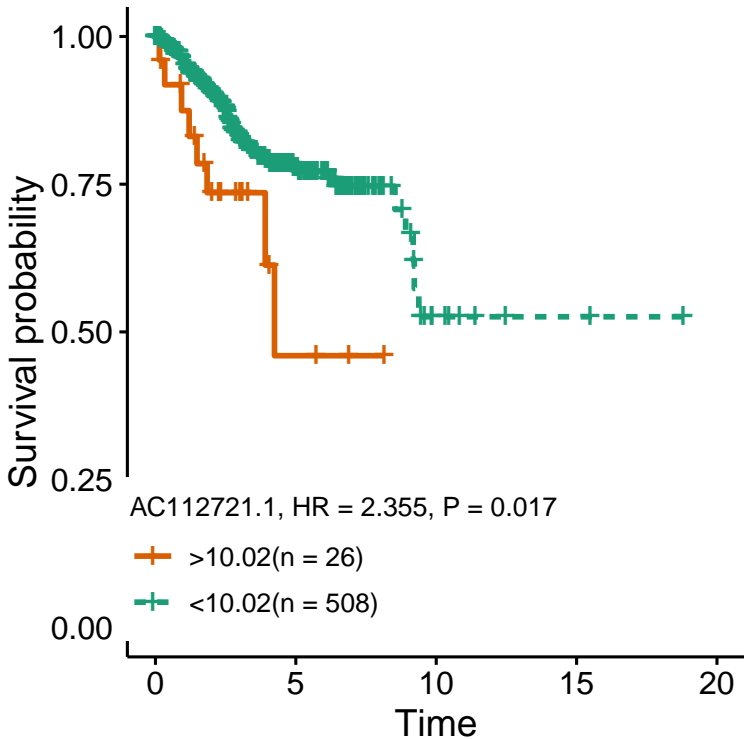

Supplement: Table S9 [file peerj-06-6091-s009.zip › Table S9/good_AC112721.1.pdf]

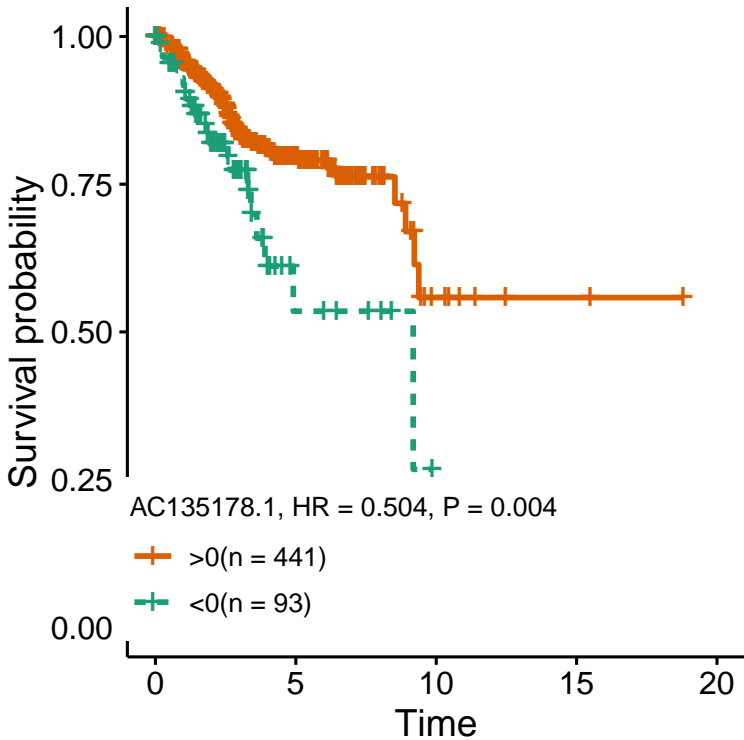

Supplement: Table S9 [file peerj-06-6091-s009.zip › Table S9/good_AC135178.1.pdf]

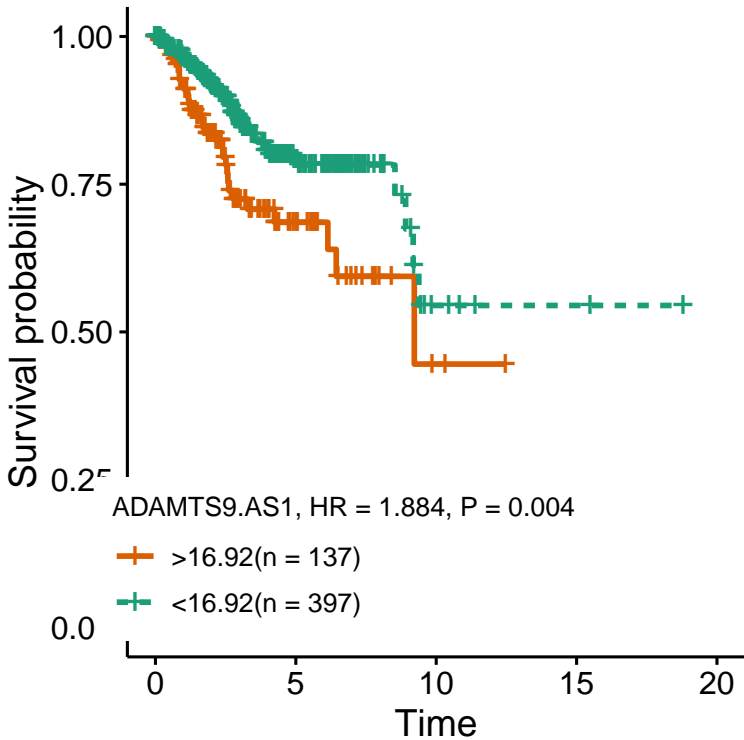

Supplement: Table S9 [file peerj-06-6091-s009.zip › Table S9/good_ADAMTS9.AS1.pdf]

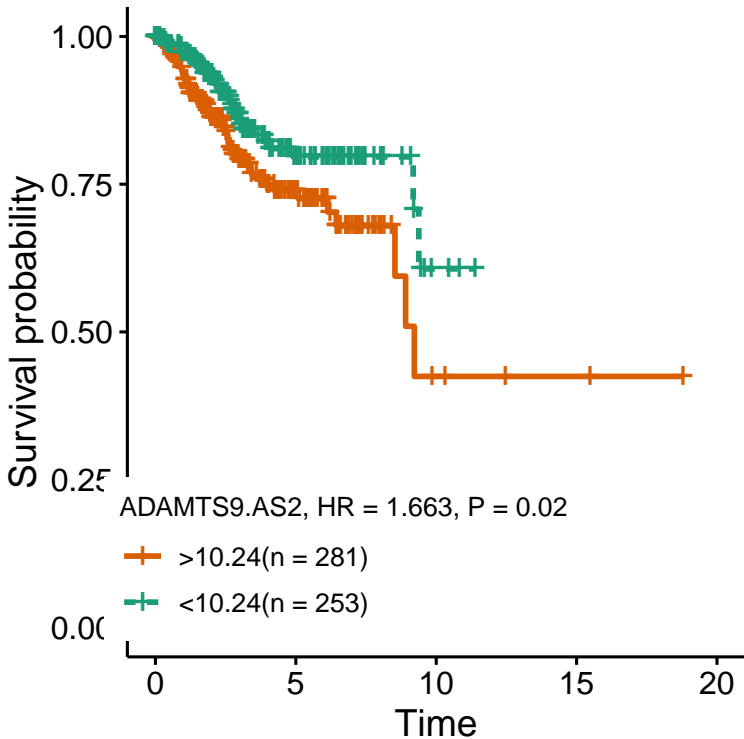

Supplement: Table S9 [file peerj-06-6091-s009.zip › Table S9/good_ADAMTS9.AS2.pdf]

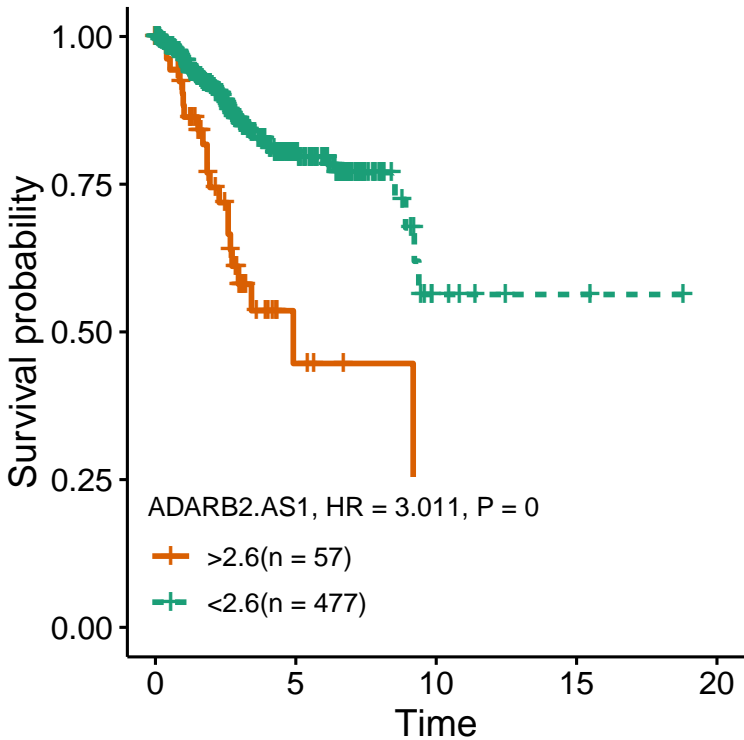

Supplement: Table S9 [file peerj-06-6091-s009.zip › Table S9/good_ADARB2.AS1.pdf]

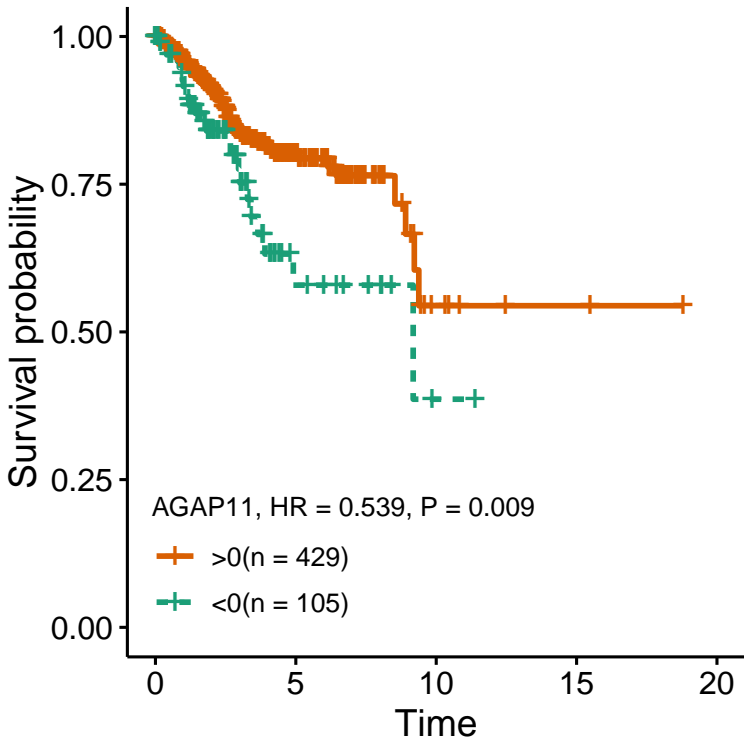

Supplement: Table S9 [file peerj-06-6091-s009.zip › Table S9/good_AGAP11.pdf]

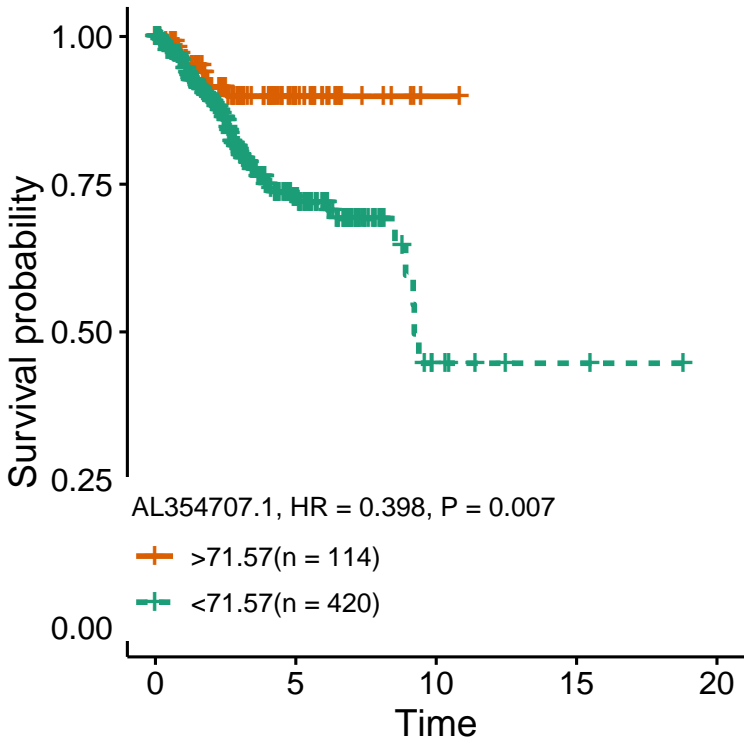

Supplement: Table S9 [file peerj-06-6091-s009.zip › Table S9/good_AL354707.1.pdf]

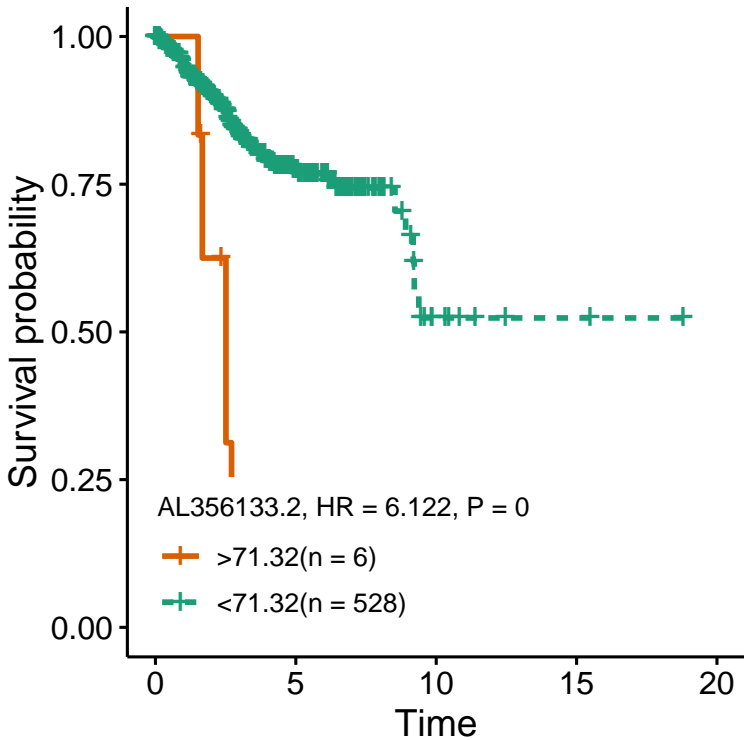

Supplement: Table S9 [file peerj-06-6091-s009.zip › Table S9/good_AL356133.2.pdf]

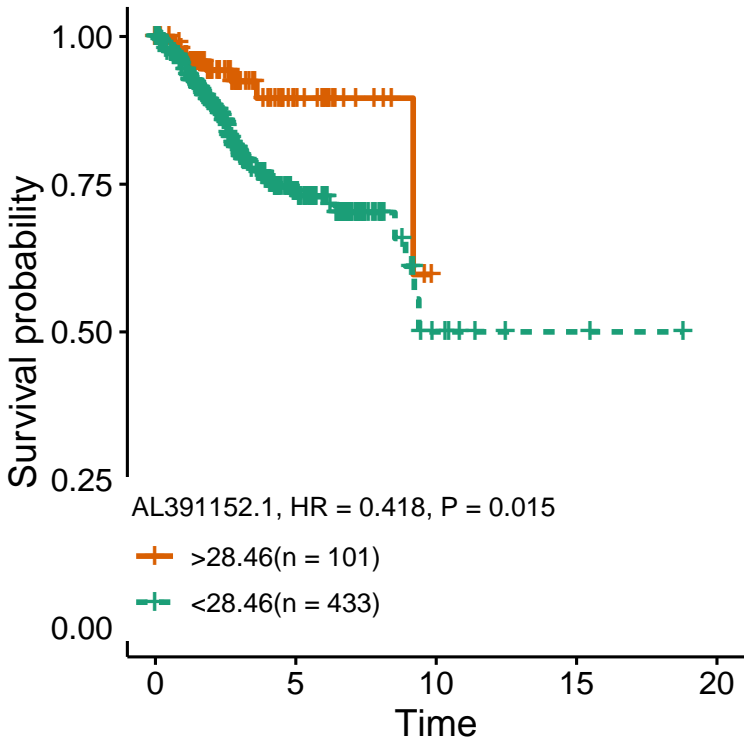

Supplement: Table S9 [file peerj-06-6091-s009.zip › Table S9/good_AL391152.1.pdf]

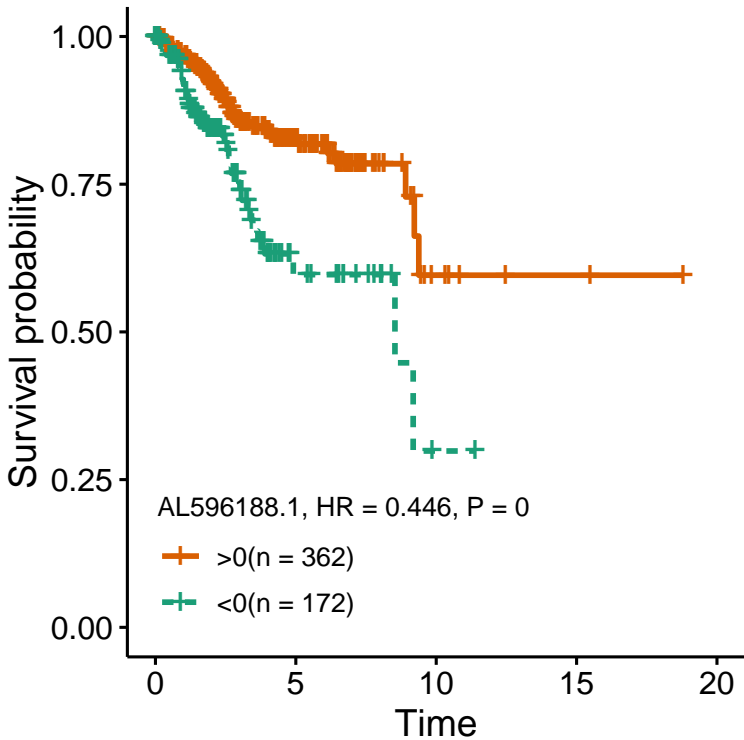

Supplement: Table S9 [file peerj-06-6091-s009.zip › Table S9/good_AL596188.1.pdf]

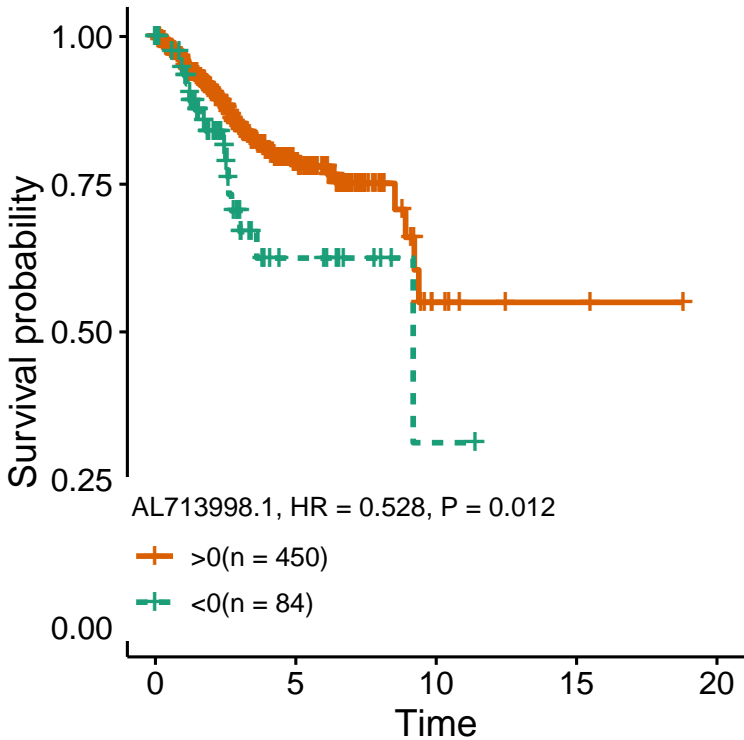

Supplement: Table S9 [file peerj-06-6091-s009.zip › Table S9/good_AL713998.1.pdf]

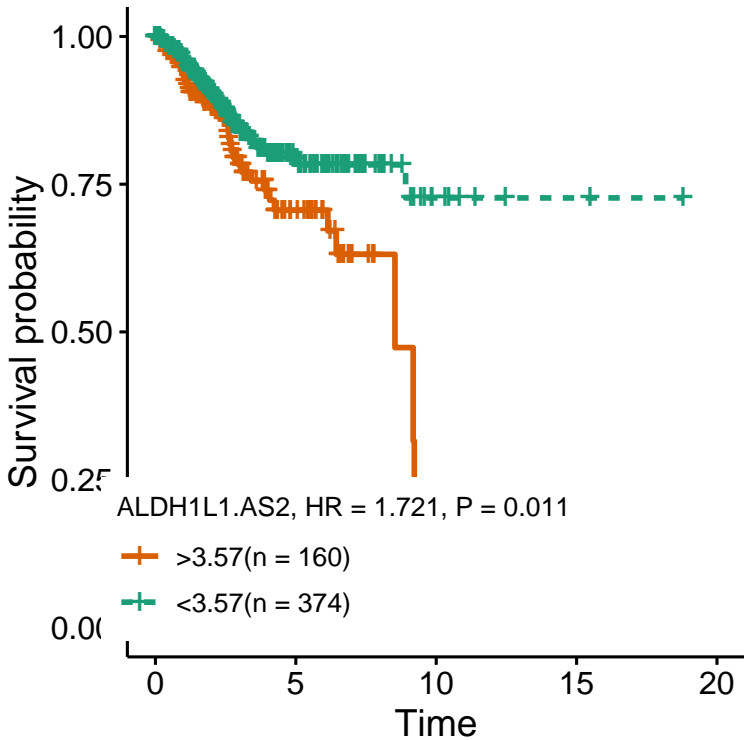

Supplement: Table S9 [file peerj-06-6091-s009.zip › Table S9/good_ALDH1L1.AS2.pdf]

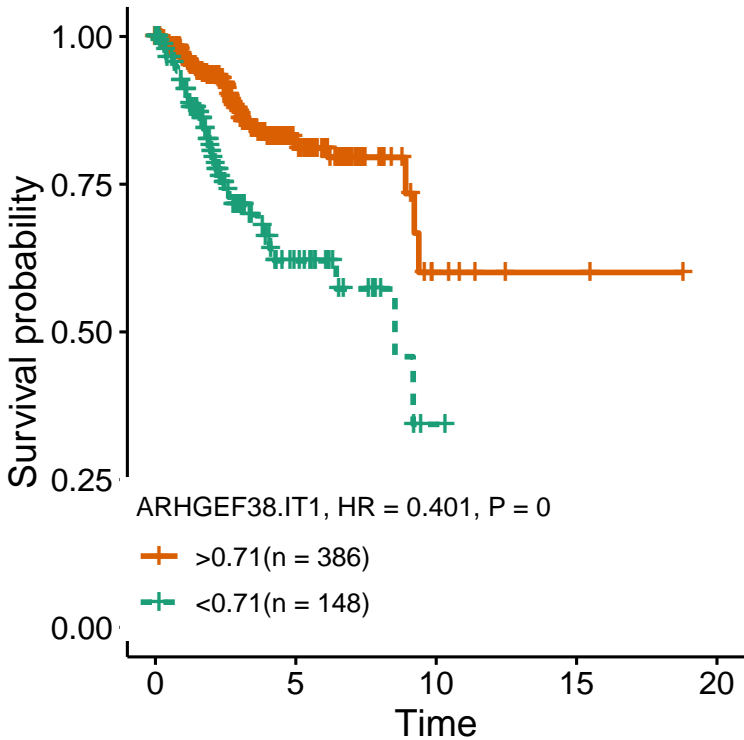

Supplement: Table S9 [file peerj-06-6091-s009.zip › Table S9/good_ARHGEF38.IT1.pdf]

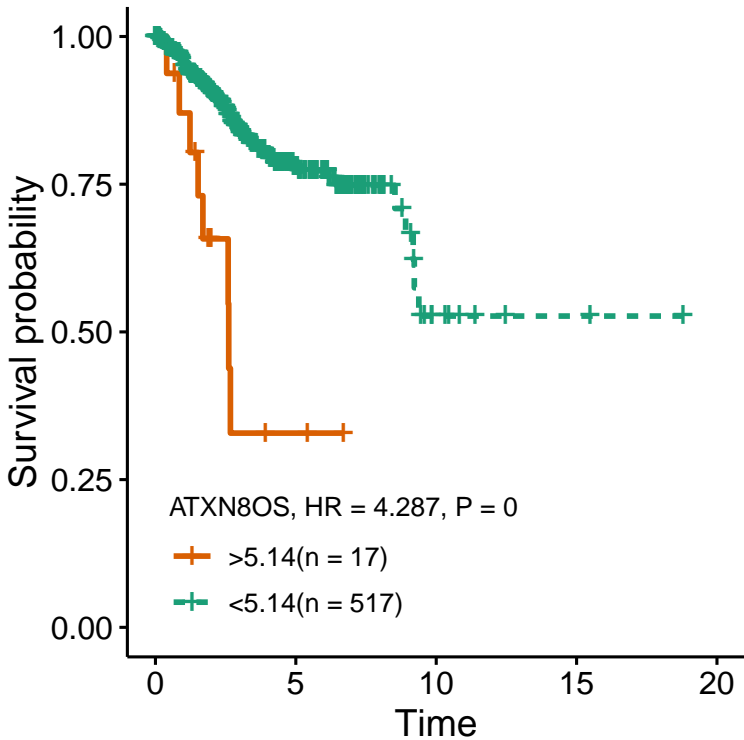

Supplement: Table S9 [file peerj-06-6091-s009.zip › Table S9/good_ATXN8OS.pdf]

Survival probability

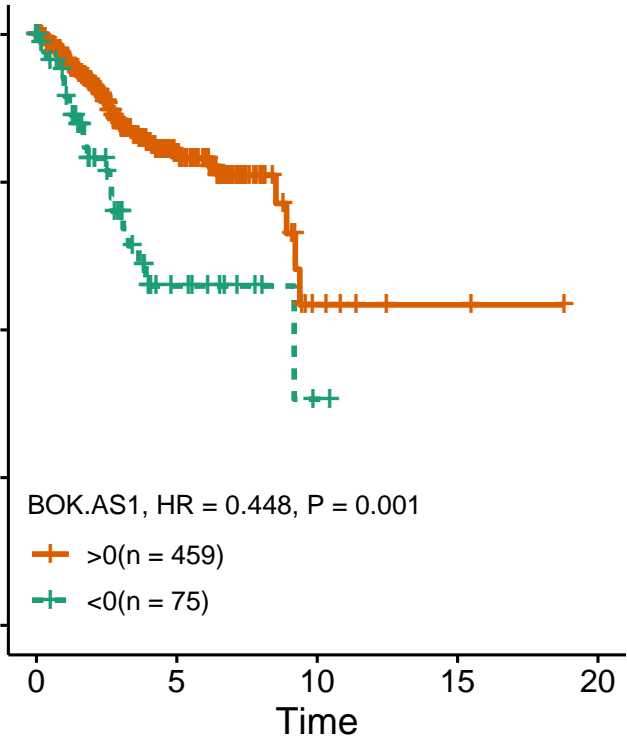

Supplement: Table S9 [file peerj-06-6091-s009.zip › Table S9/good_BOK.AS1.pdf]

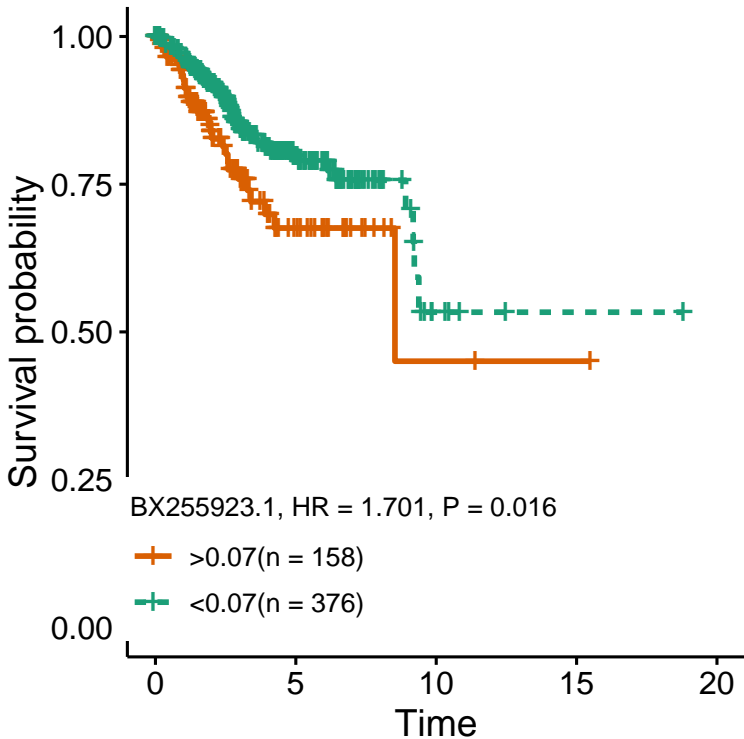

Supplement: Table S9 [file peerj-06-6091-s009.zip › Table S9/good_BX255923.1.pdf]

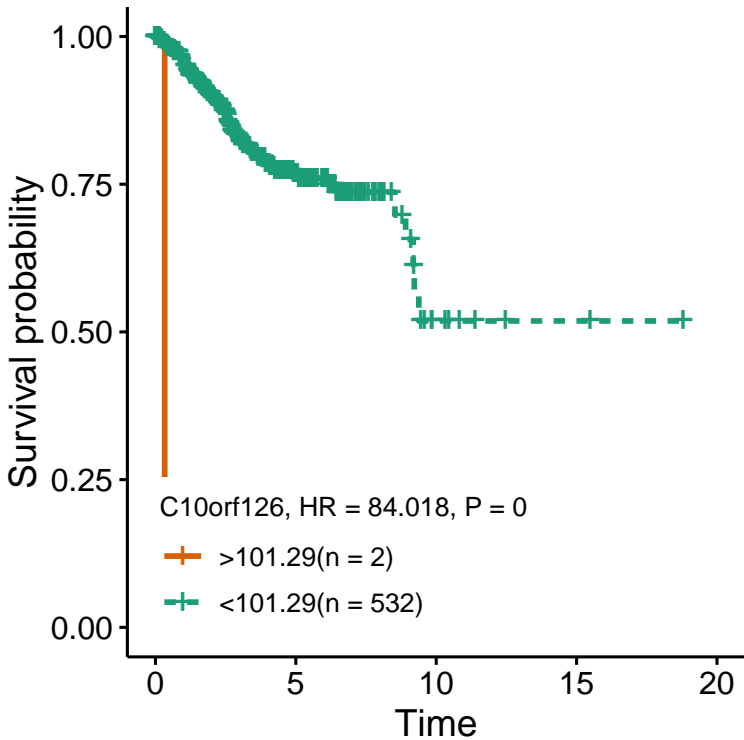

Supplement: Table S9 [file peerj-06-6091-s009.zip › Table S9/good_C10orf126.pdf]

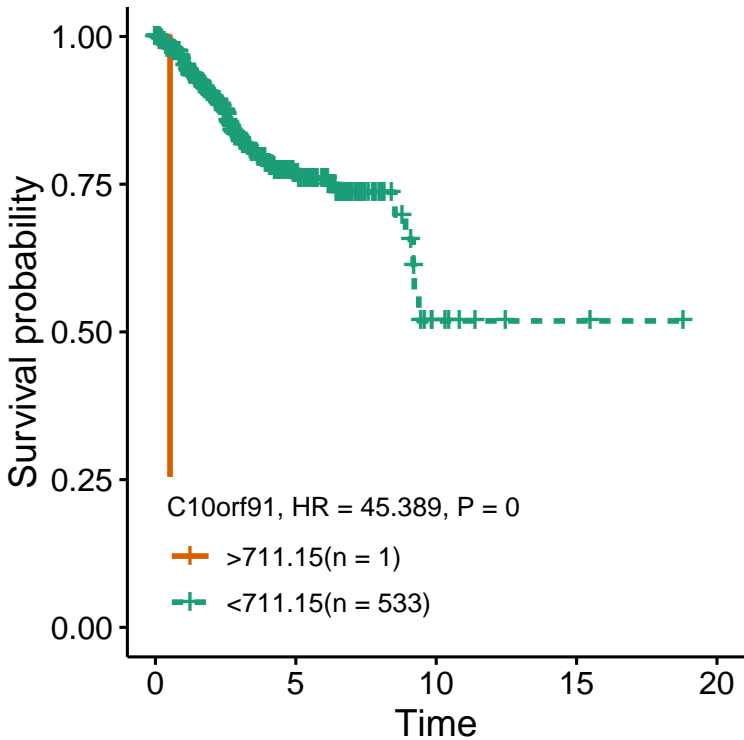

Supplement: Table S9 [file peerj-06-6091-s009.zip › Table S9/good_C10orf91.pdf]

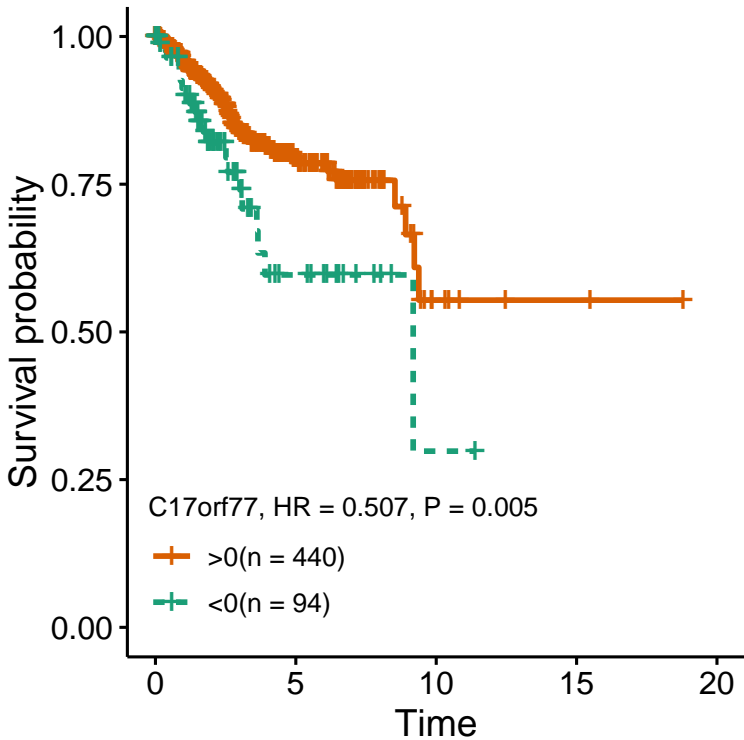

Supplement: Table S9 [file peerj-06-6091-s009.zip › Table S9/good_C17orf77.pdf]

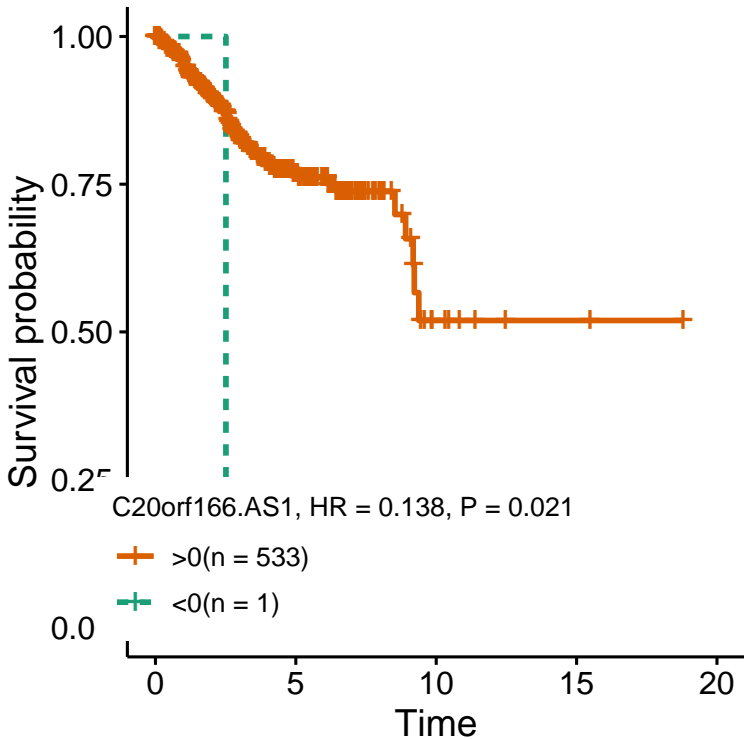

Supplement: Table S9 [file peerj-06-6091-s009.zip › Table S9/good_C20orf166.AS1.pdf]

Survival probability

1.00  
0.75  
0.50  
0.25  
0.00

0

5

Time

10

15

20

C2orf48, HR = 2.049, P = 0.003

+ >10.62(n = 333)

+ <10.62(n = 201)

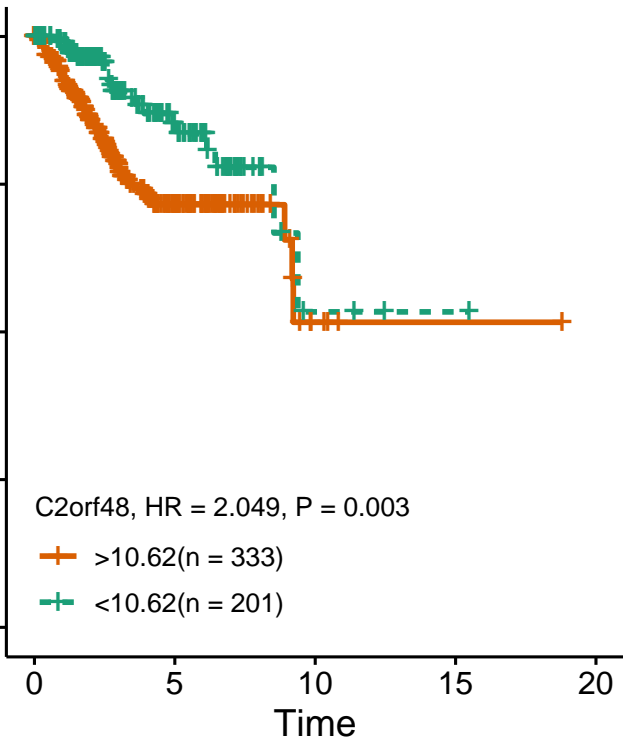

Supplement: Table S9 [file peerj-06-6091-s009.zip › Table S9/good_C2orf48.pdf]

Survival probability

1.00  
0.75  
0.50  
0.25  
0.00

0

5

Time

10

15

20

C6orf99, HR = Inf, P = 0.039

+ >3.74(n = 514)

+ <3.74(n = 20)

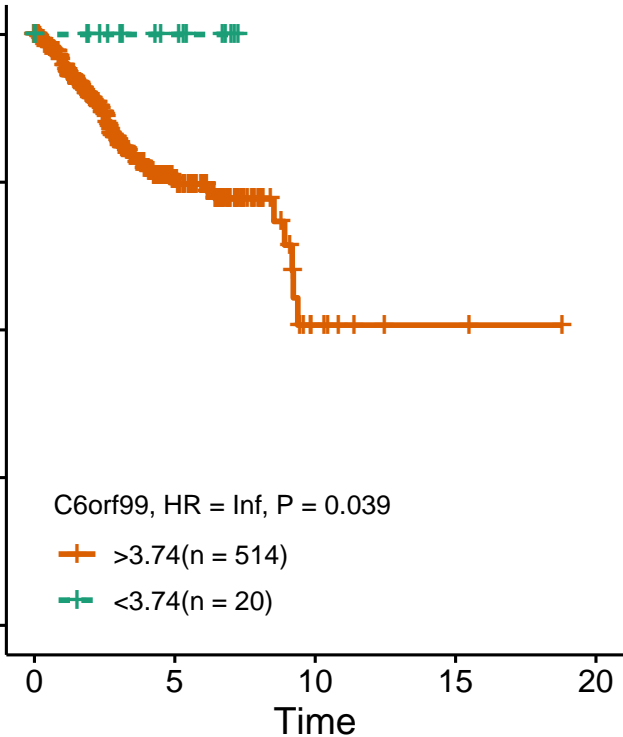

Supplement: Table S9 [file peerj-06-6091-s009.zip › Table S9/good_C6orf99.pdf]

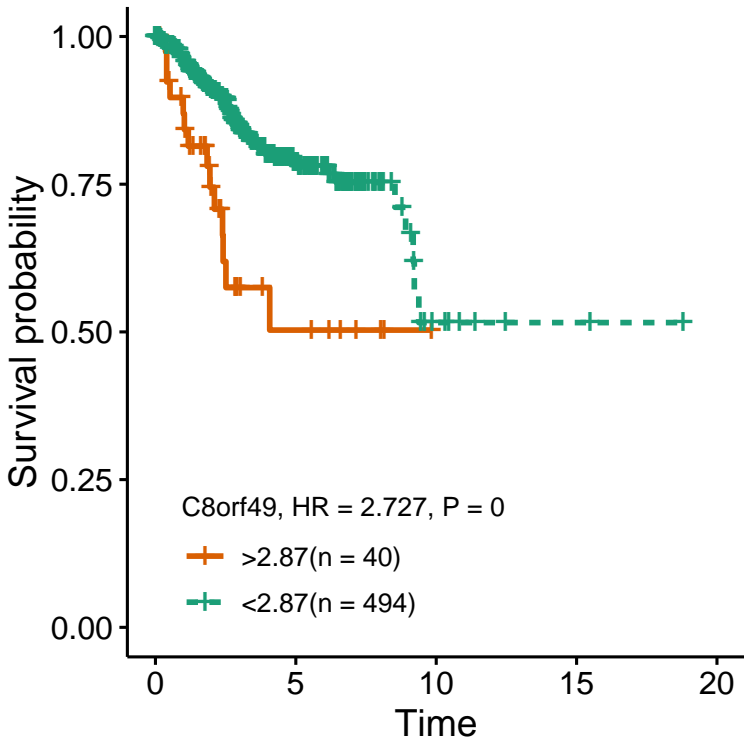

Supplement: Table S9 [file peerj-06-6091-s009.zip › Table S9/good_C8orf49.pdf]

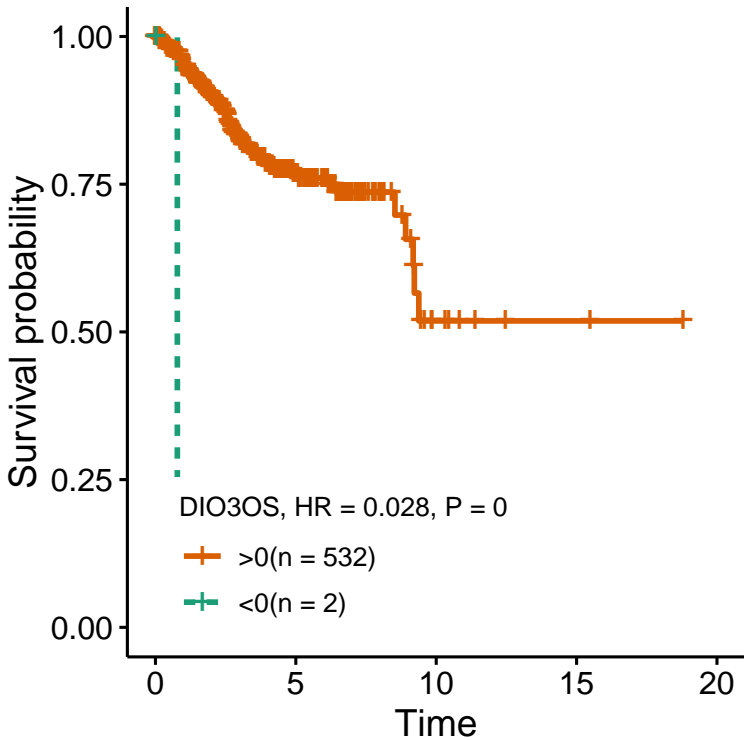

Supplement: Table S9 [file peerj-06-6091-s009.zip › Table S9/good_DIO3OS.pdf]

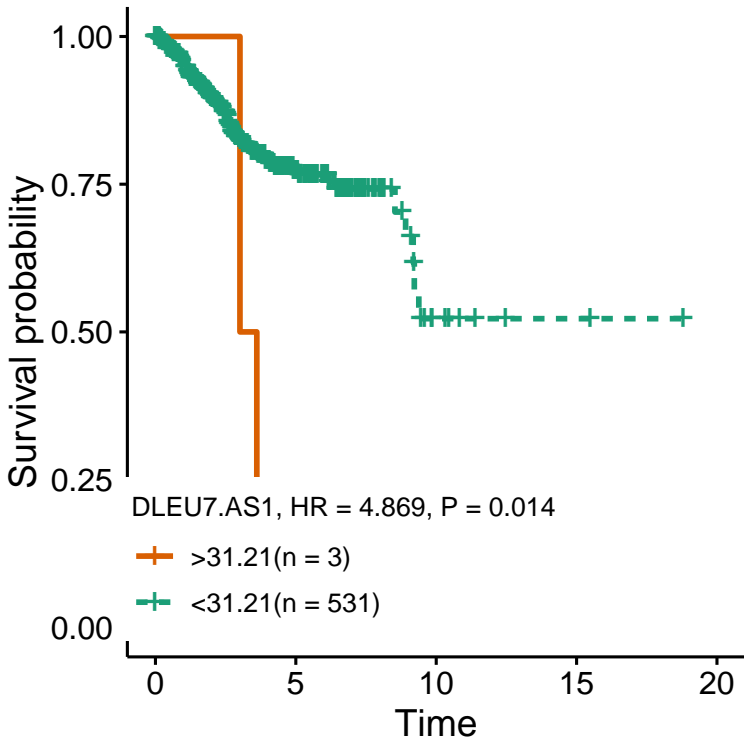

Supplement: Table S9 [file peerj-06-6091-s009.zip › Table S9/good_DLEU7.AS1.pdf]

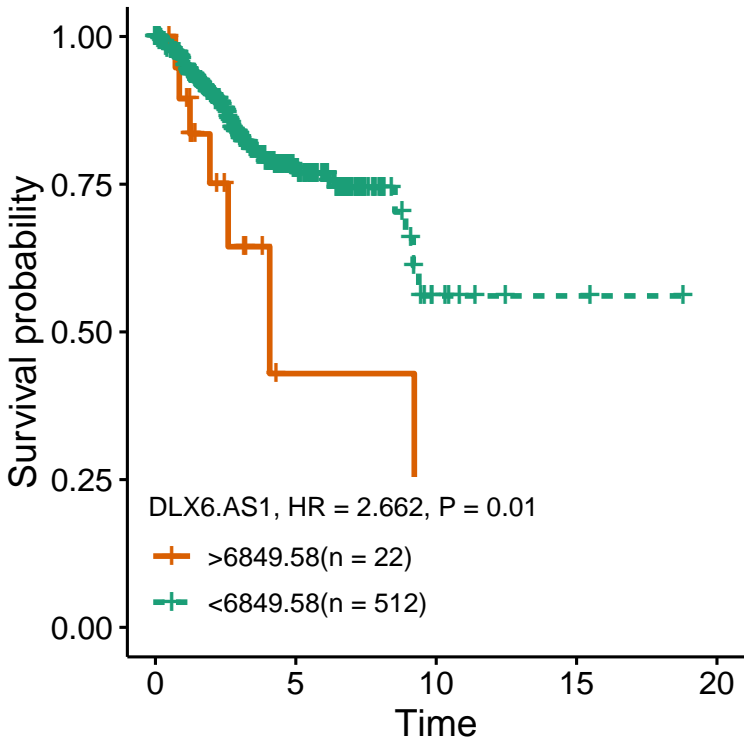

Supplement: Table S9 [file peerj-06-6091-s009.zip › Table S9/good_DLX6.AS1.pdf]

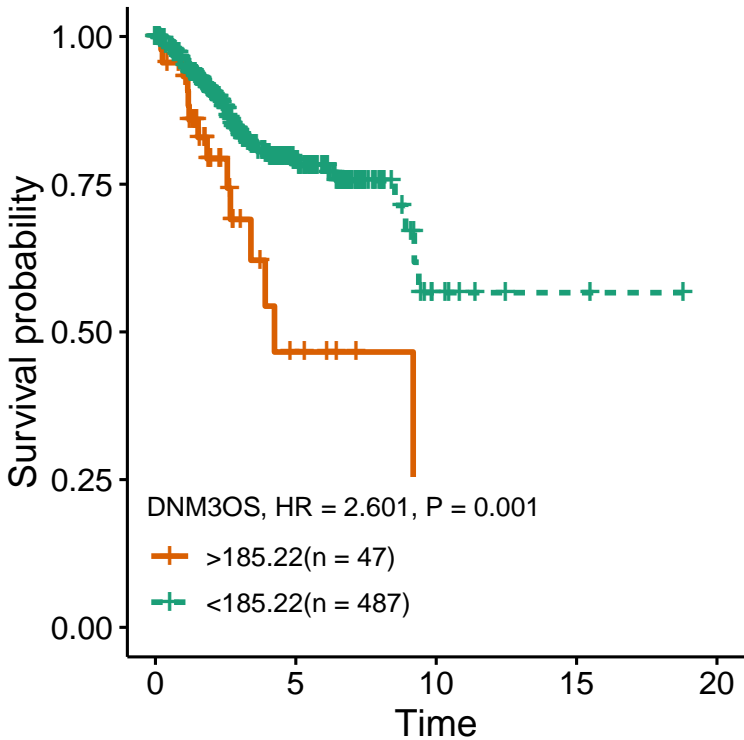

Supplement: Table S9 [file peerj-06-6091-s009.zip › Table S9/good_DNM3OS.pdf]

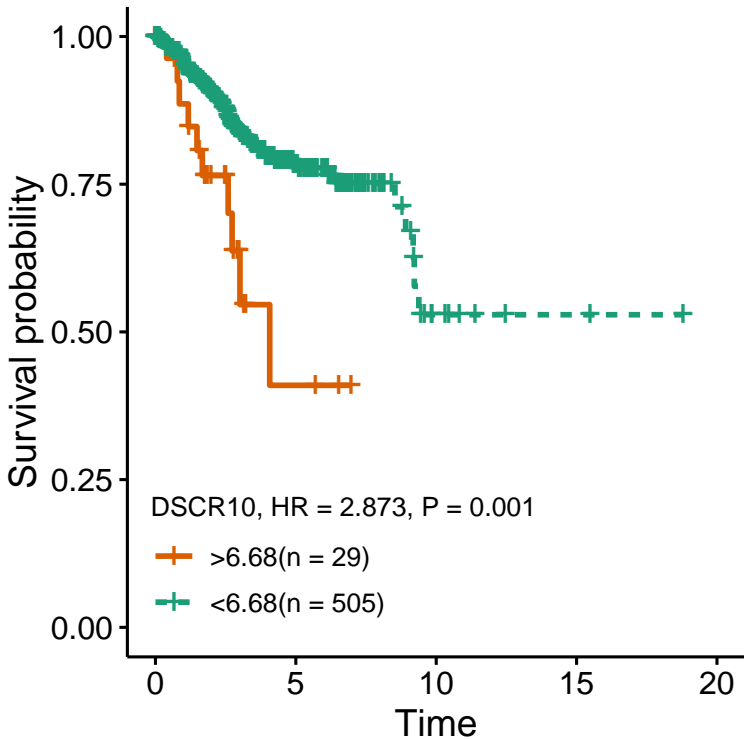

Supplement: Table S9 [file peerj-06-6091-s009.zip › Table S9/good_DSCR10.pdf]

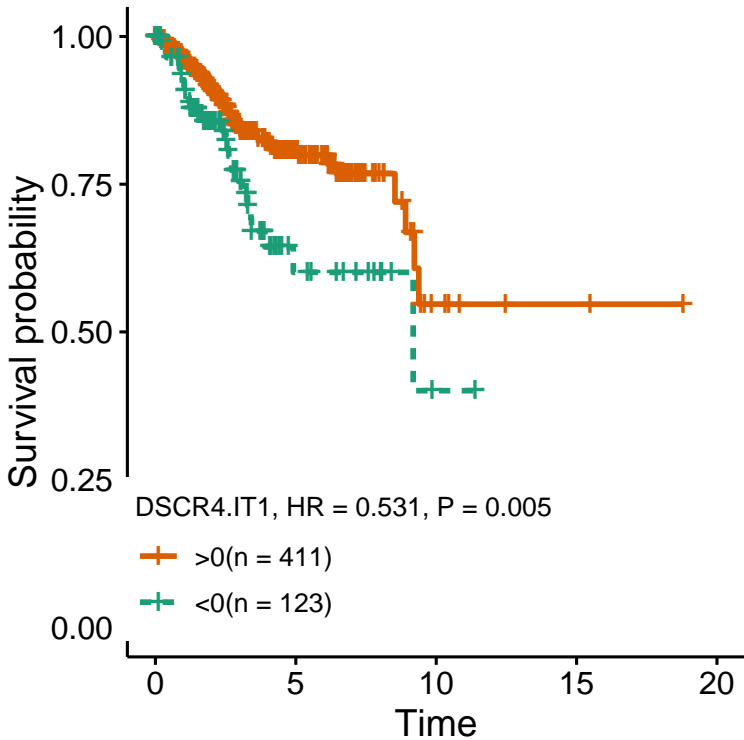

Supplement: Table S9 [file peerj-06-6091-s009.zip › Table S9/good_DSCR4.IT1.pdf]

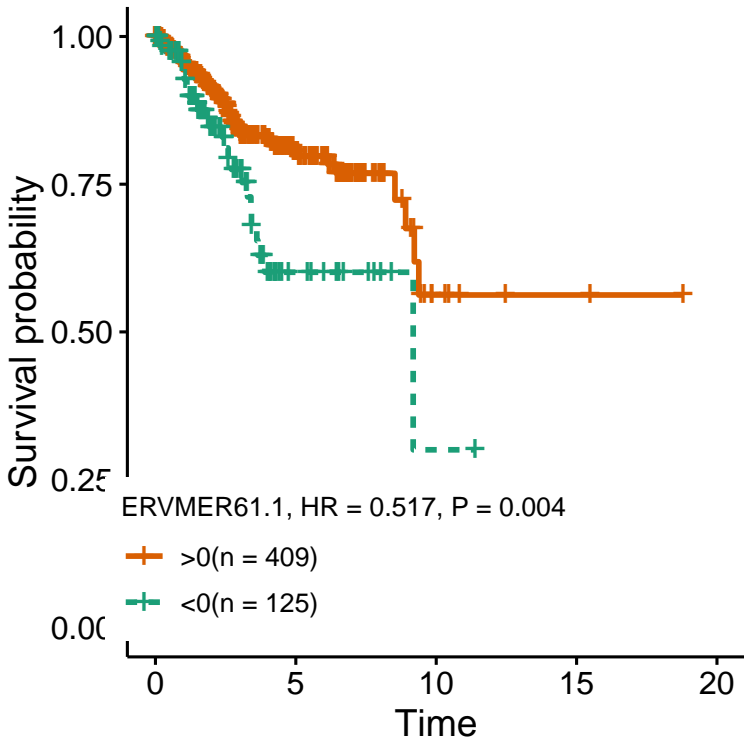

Supplement: Table S9 [file peerj-06-6091-s009.zip › Table S9/good_ERVMER61.1.pdf]

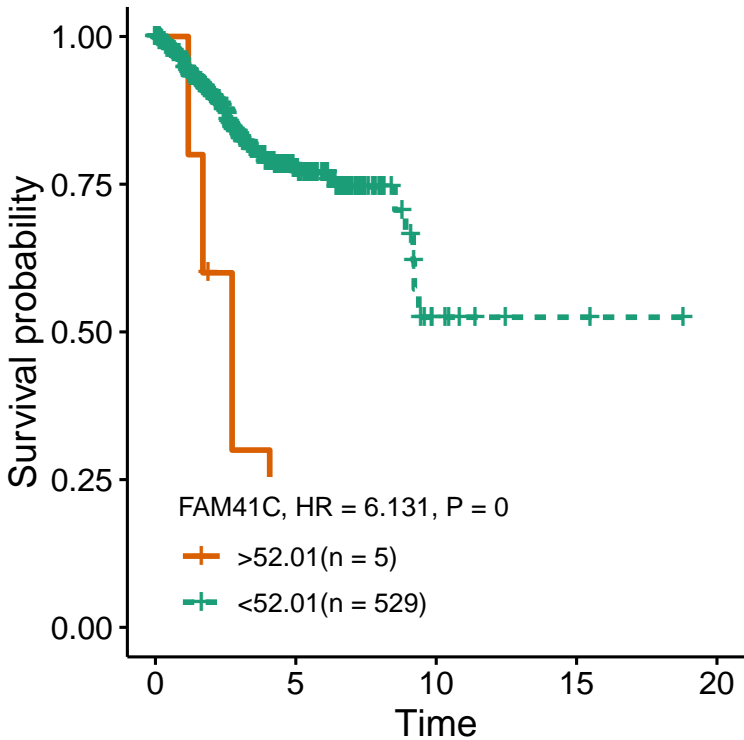

Supplement: Table S9 [file peerj-06-6091-s009.zip › Table S9/good_FAM41C.pdf]

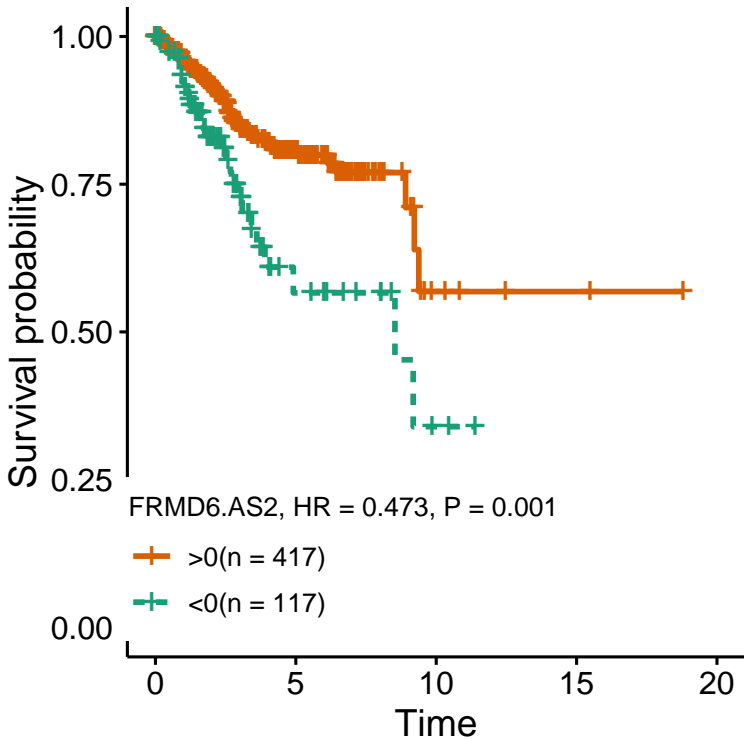

Supplement: Table S9 [file peerj-06-6091-s009.zip › Table S9/good_FRMD6.AS2.pdf]

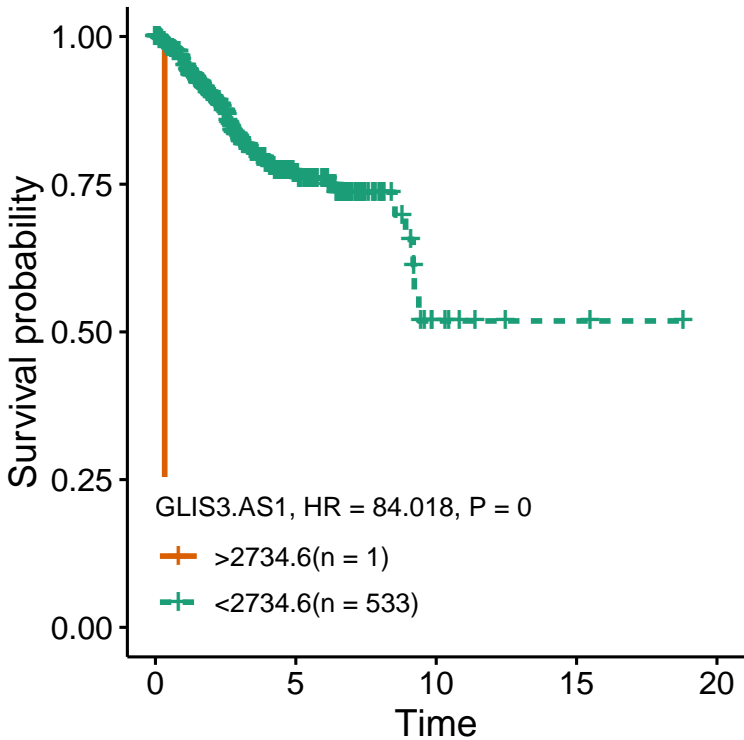

Supplement: Table S9 [file peerj-06-6091-s009.zip › Table S9/good_GLIS3.AS1.pdf]

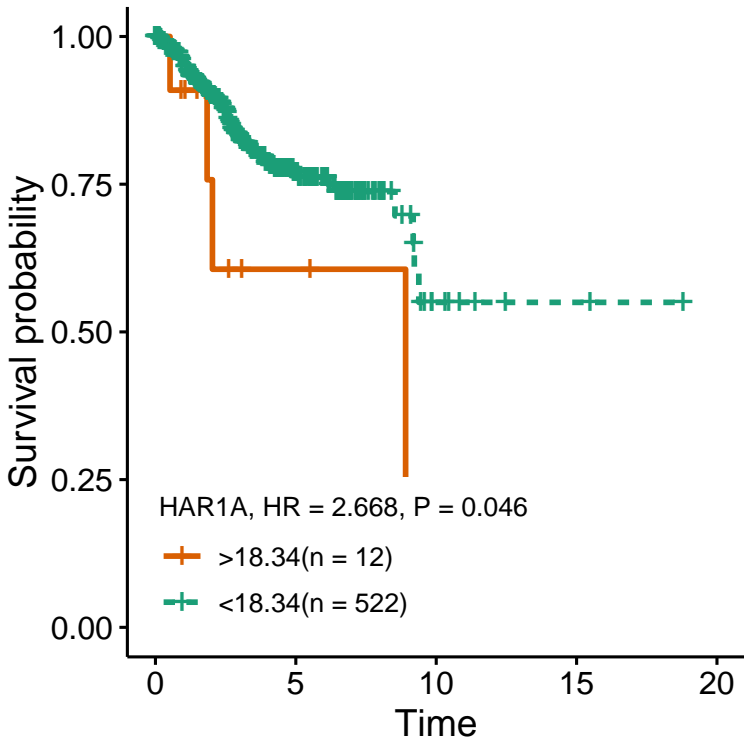

Supplement: Table S9 [file peerj-06-6091-s009.zip › Table S9/good_HAR1A.pdf]

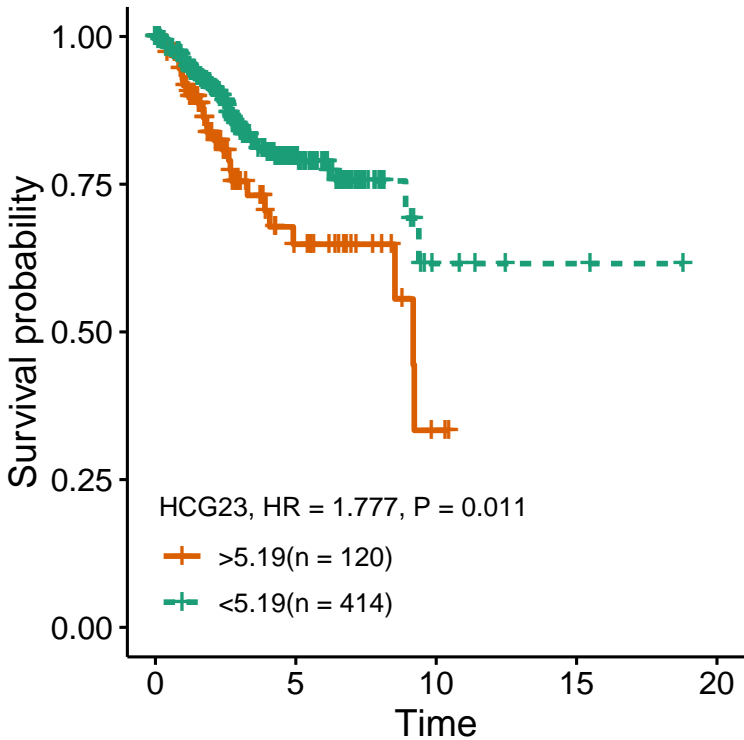

Supplement: Table S9 [file peerj-06-6091-s009.zip › Table S9/good_HCG23.pdf]

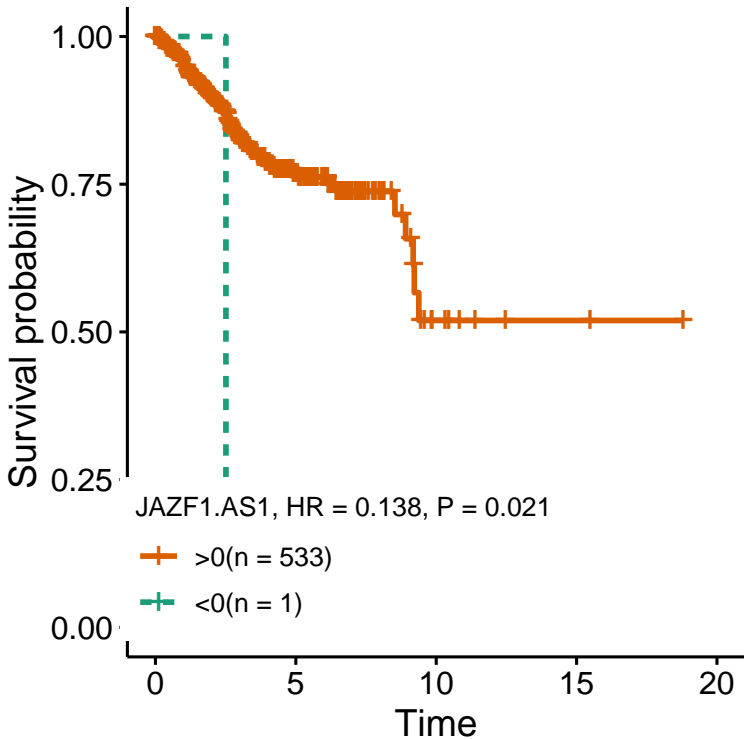

Supplement: Table S9 [file peerj-06-6091-s009.zip › Table S9/good_JAZF1.AS1.pdf]

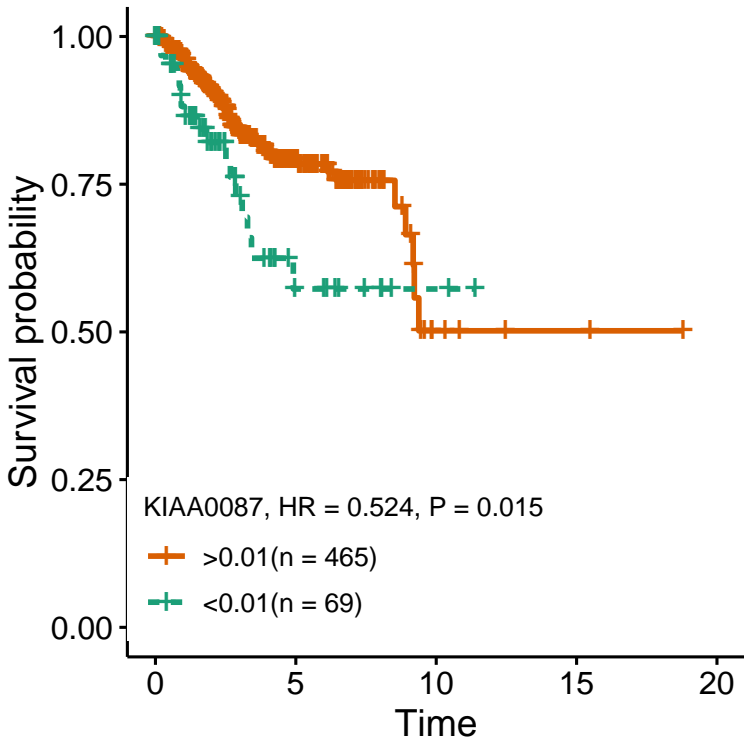

Supplement: Table S9 [file peerj-06-6091-s009.zip › Table S9/good_KIAA0087.pdf]

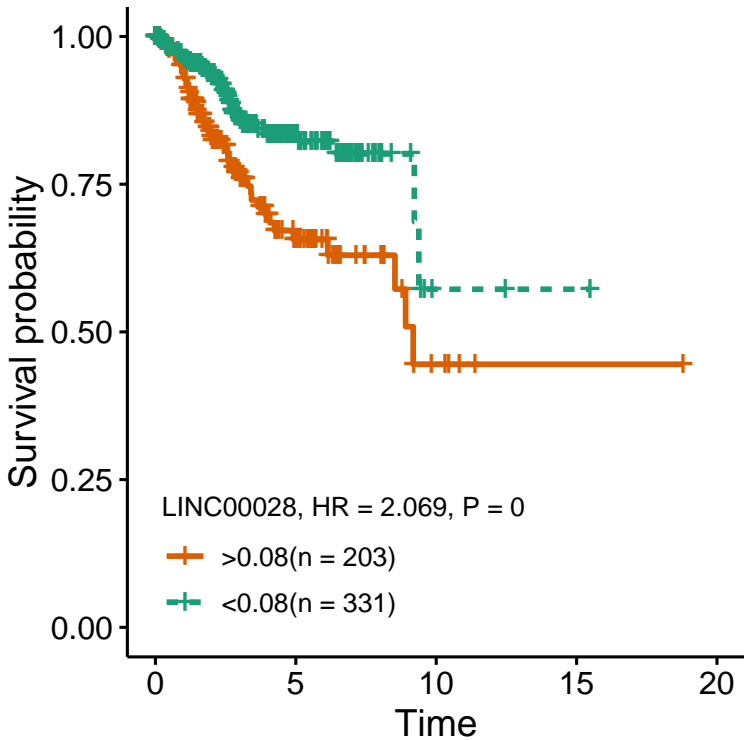

Supplement: Table S9 [file peerj-06-6091-s009.zip › Table S9/good_LINC00028.pdf]

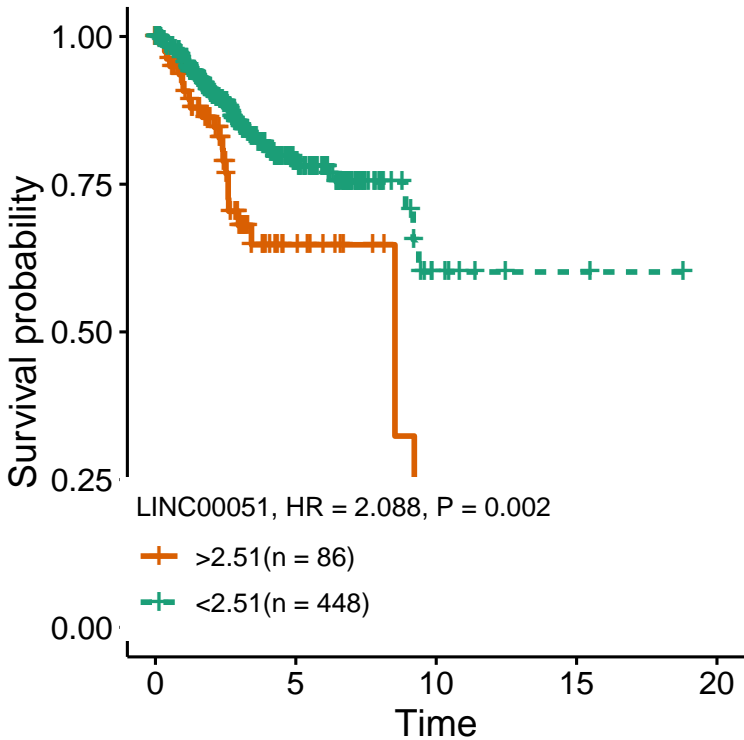

Supplement: Table S9 [file peerj-06-6091-s009.zip › Table S9/good_LINC00051.pdf]

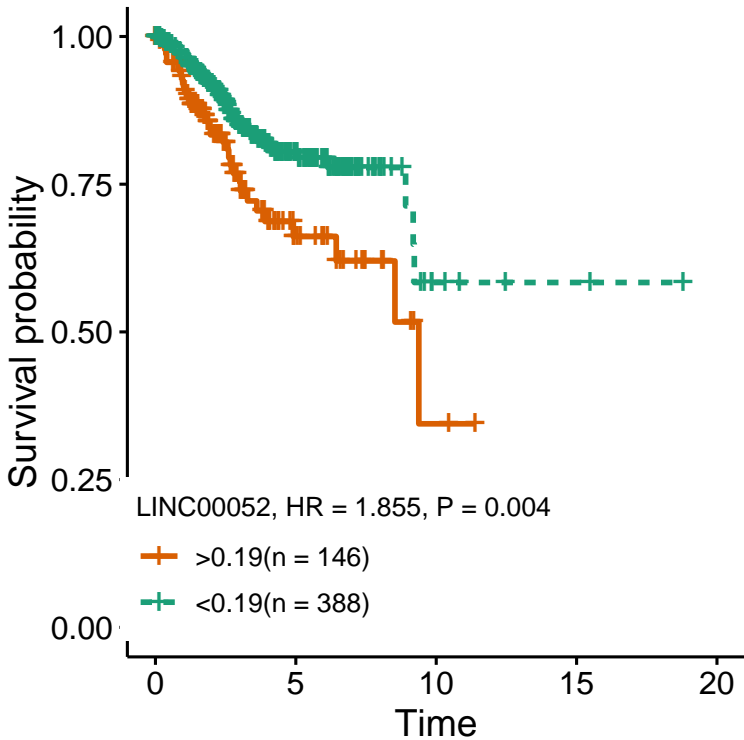

Supplement: Table S9 [file peerj-06-6091-s009.zip › Table S9/good_LINC00052.pdf]

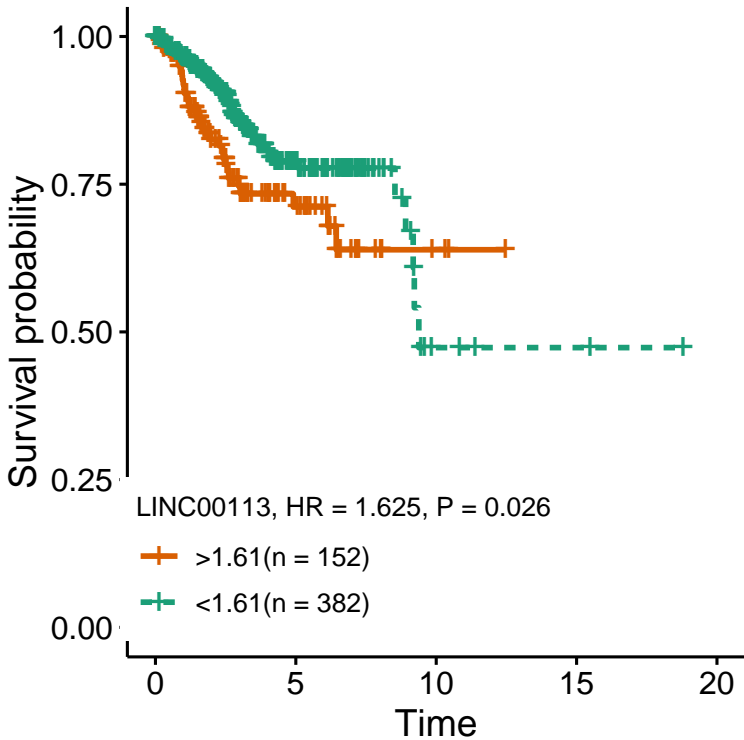

Supplement: Table S9 [file peerj-06-6091-s009.zip › Table S9/good_LINC00113.pdf]

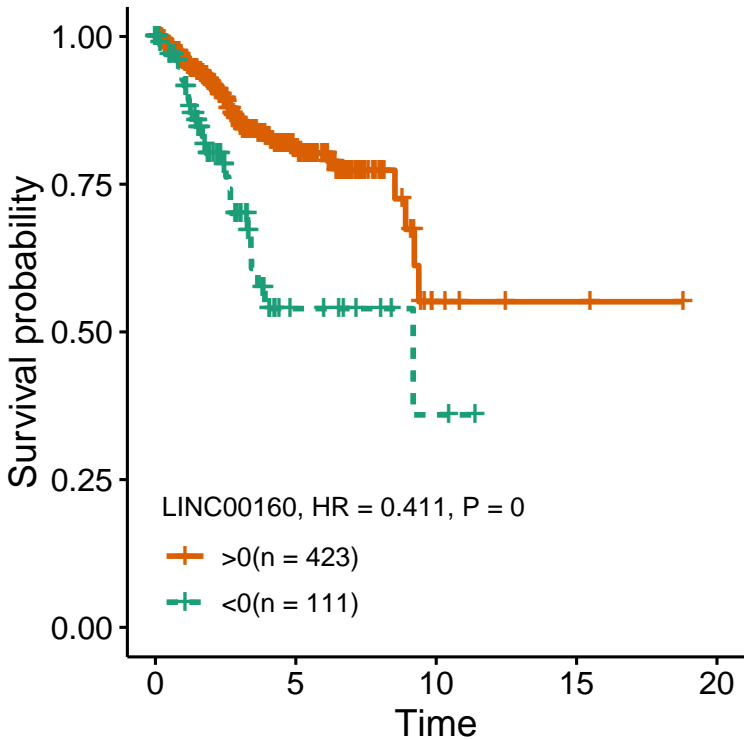

Supplement: Table S9 [file peerj-06-6091-s009.zip › Table S9/good_LINC00160.pdf]

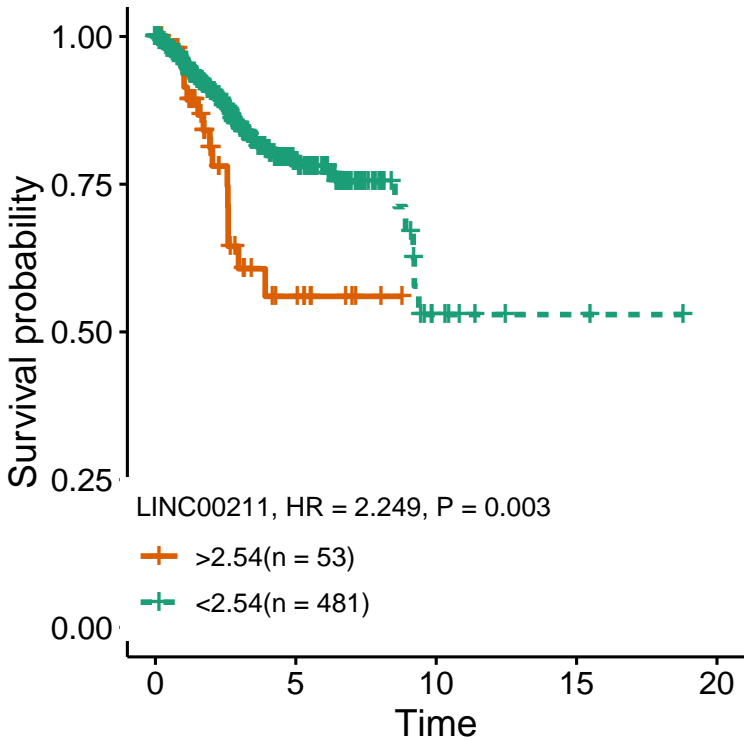

Supplement: Table S9 [file peerj-06-6091-s009.zip › Table S9/good_LINC00211.pdf]

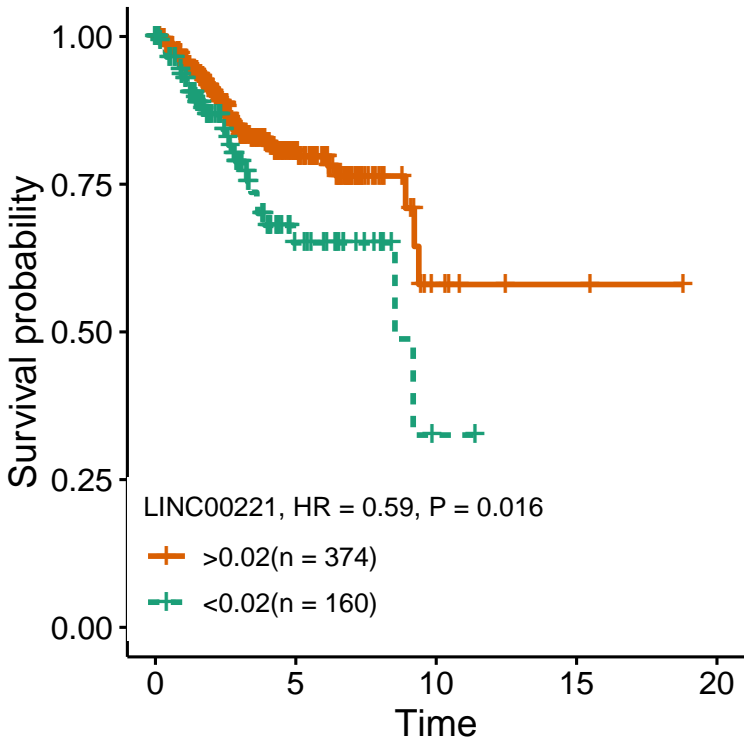

Supplement: Table S9 [file peerj-06-6091-s009.zip › Table S9/good_LINC00221.pdf]

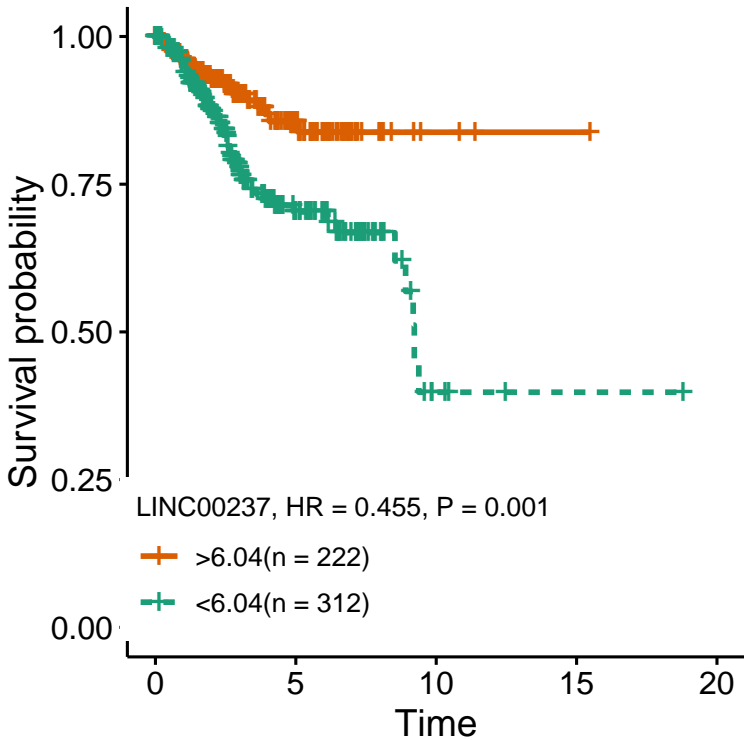

Supplement: Table S9 [file peerj-06-6091-s009.zip › Table S9/good_LINC00237.pdf]

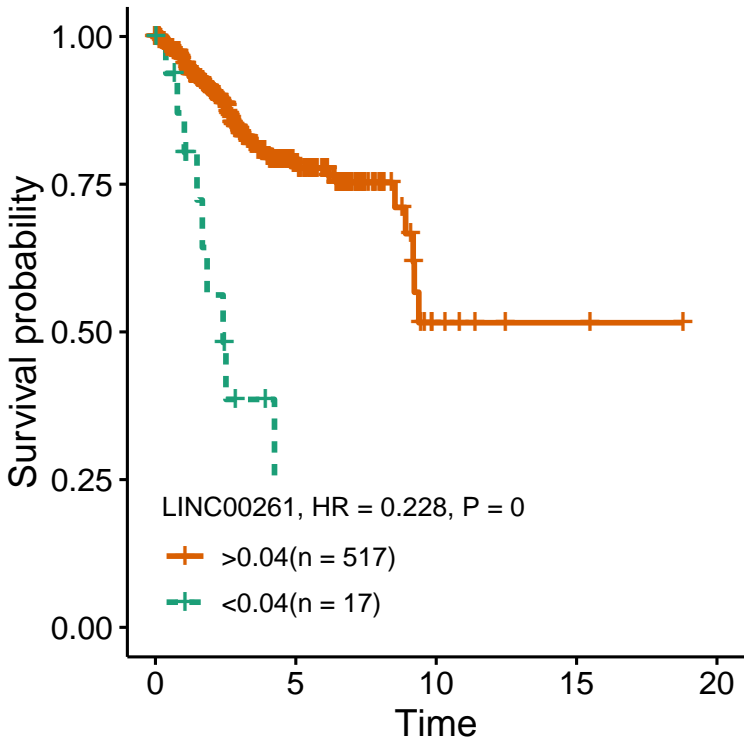

Supplement: Table S9 [file peerj-06-6091-s009.zip › Table S9/good_LINC00261.pdf]

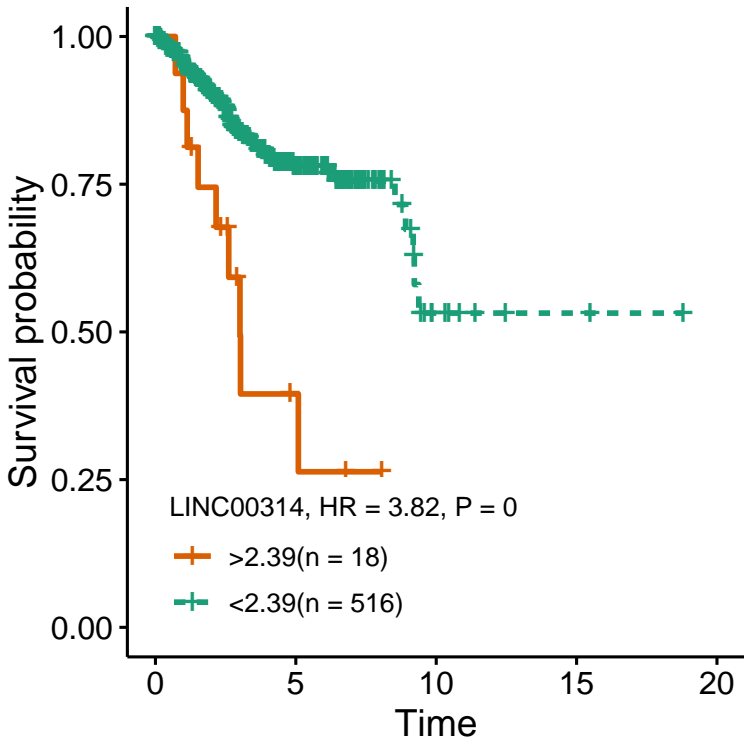

Supplement: Table S9 [file peerj-06-6091-s009.zip › Table S9/good_LINC00314.pdf]

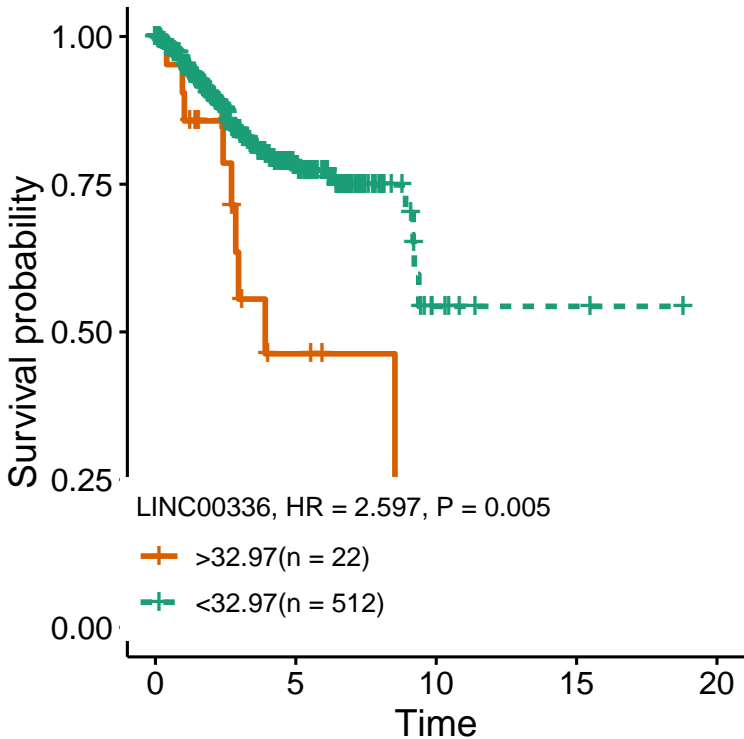

Supplement: Table S9 [file peerj-06-6091-s009.zip › Table S9/good_LINC00336.pdf]

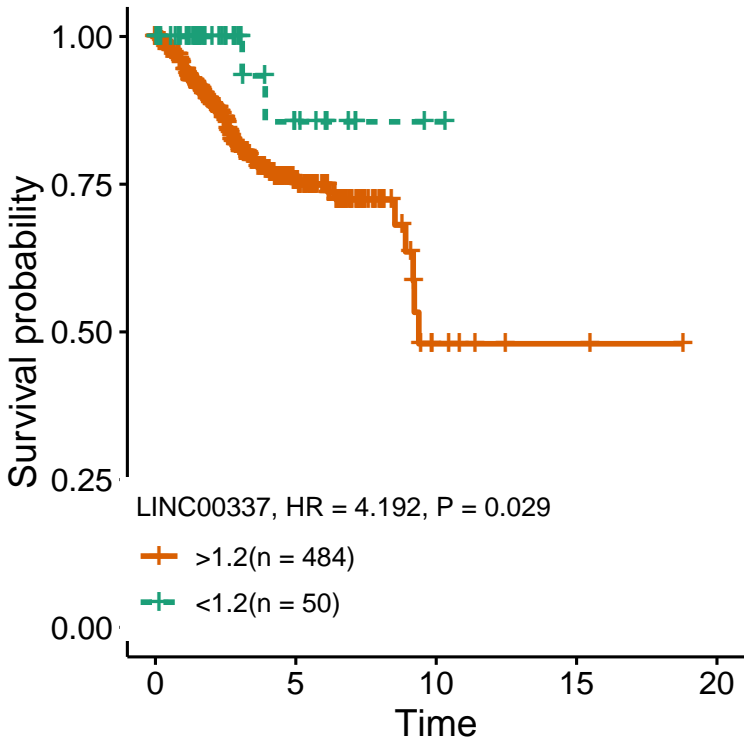

Supplement: Table S9 [file peerj-06-6091-s009.zip › Table S9/good_LINC00337.pdf]

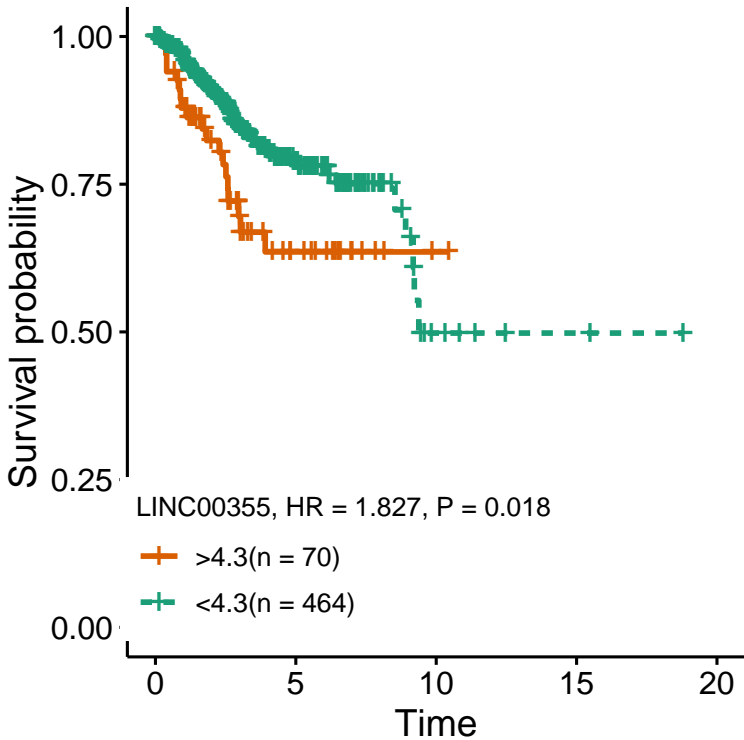

Supplement: Table S9 [file peerj-06-6091-s009.zip › Table S9/good_LINC00355.pdf]

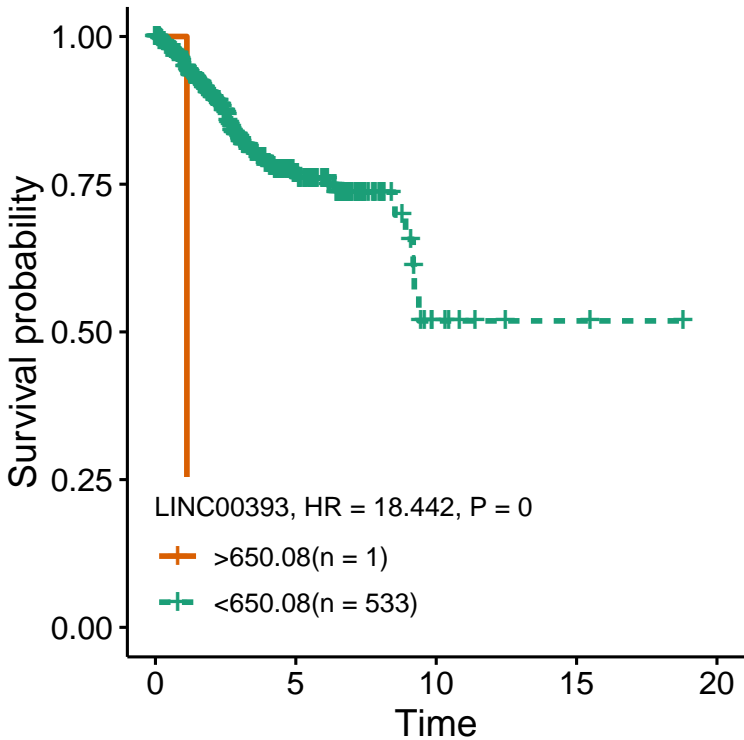

Supplement: Table S9 [file peerj-06-6091-s009.zip › Table S9/good_LINC00393.pdf]

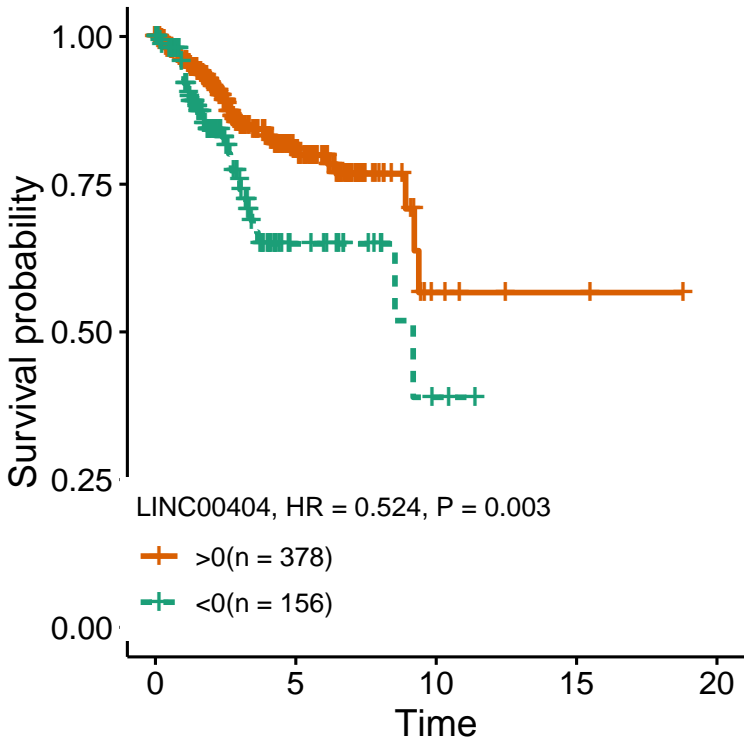

Supplement: Table S9 [file peerj-06-6091-s009.zip › Table S9/good_LINC00404.pdf]

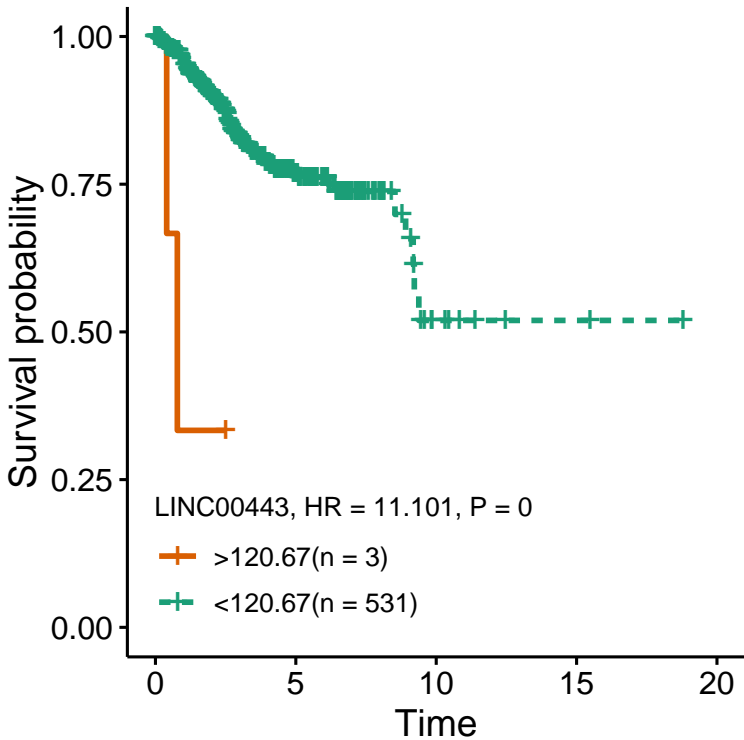

Supplement: Table S9 [file peerj-06-6091-s009.zip › Table S9/good_LINC00443.pdf]

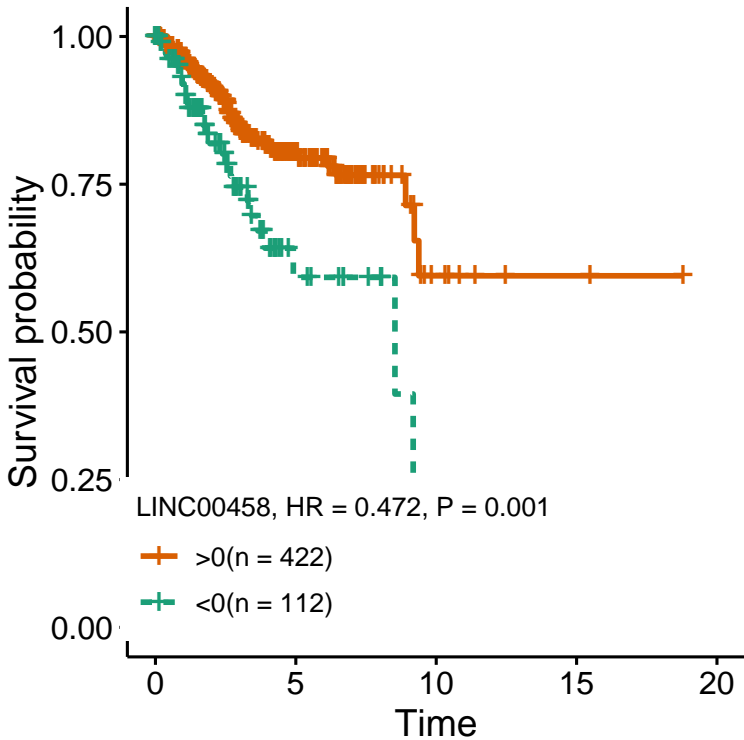

Supplement: Table S9 [file peerj-06-6091-s009.zip › Table S9/good_LINC00458.pdf]

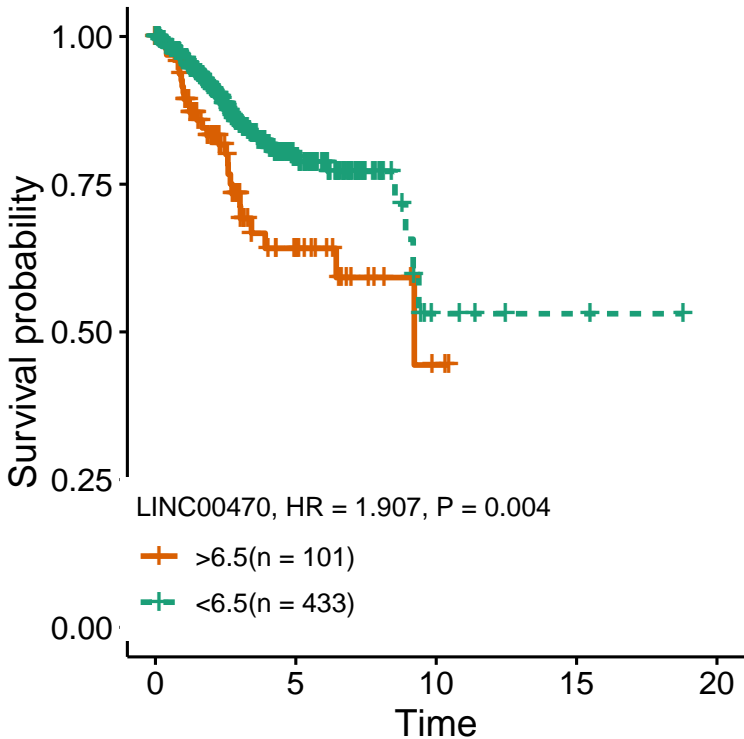

Supplement: Table S9 [file peerj-06-6091-s009.zip › Table S9/good_LINC00470.pdf]

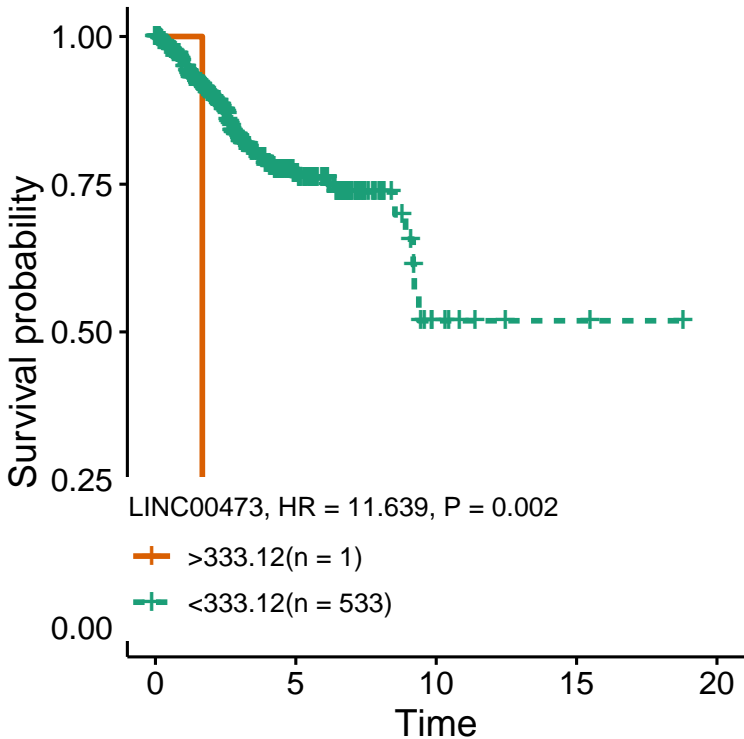

Supplement: Table S9 [file peerj-06-6091-s009.zip › Table S9/good_LINC00473.pdf]

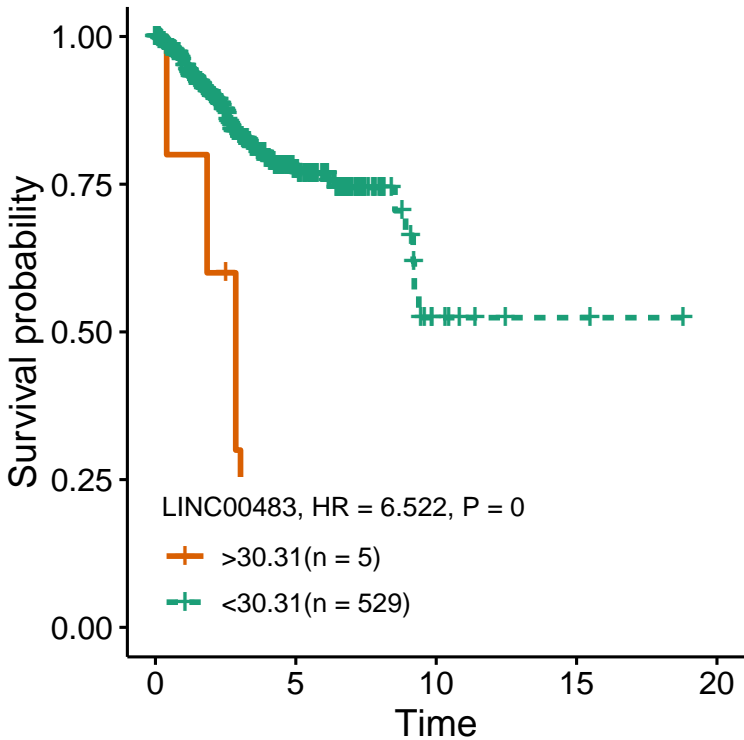

Supplement: Table S9 [file peerj-06-6091-s009.zip › Table S9/good_LINC00483.pdf]

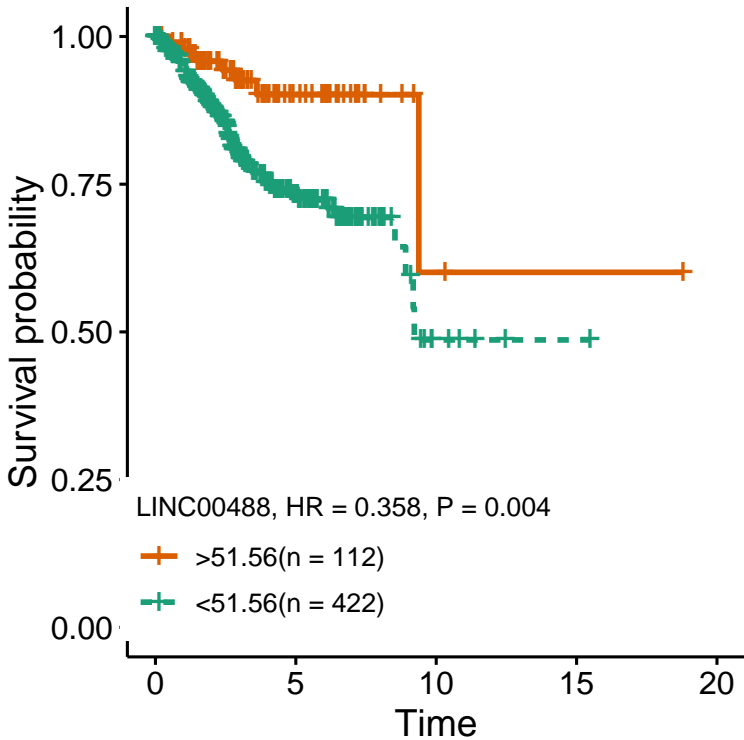

Supplement: Table S9 [file peerj-06-6091-s009.zip › Table S9/good_LINC00488.pdf]

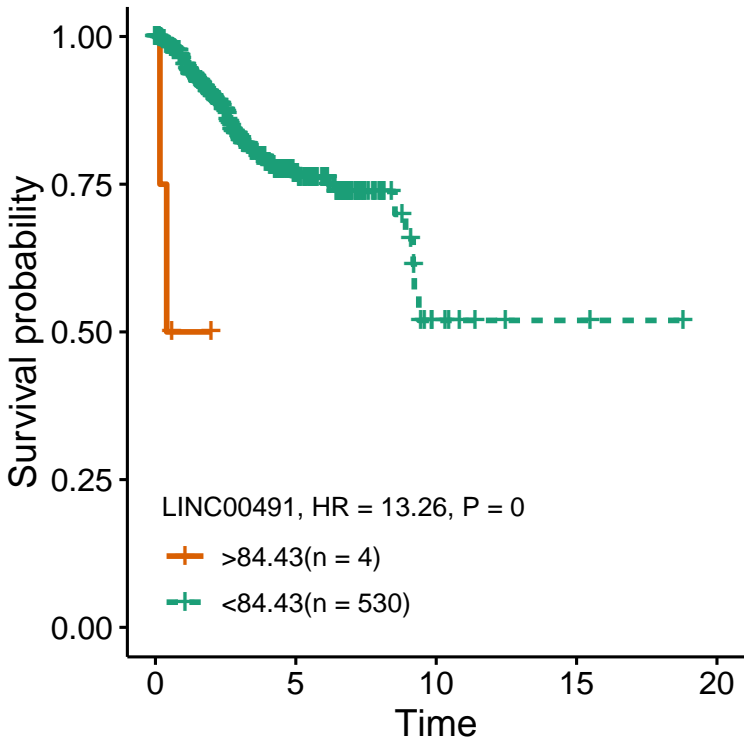

Supplement: Table S9 [file peerj-06-6091-s009.zip › Table S9/good_LINC00491.pdf]

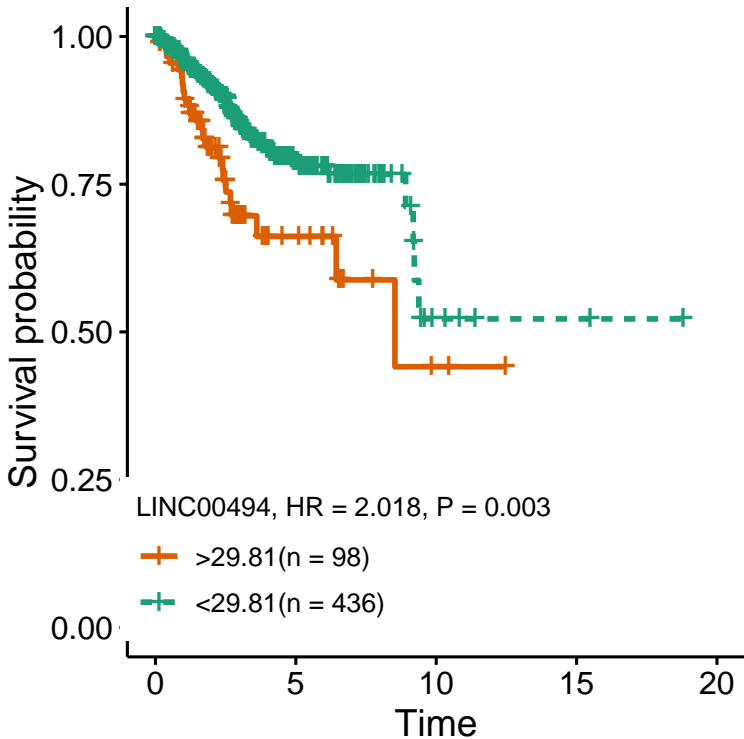

Supplement: Table S9 [file peerj-06-6091-s009.zip › Table S9/good_LINC00494.pdf]

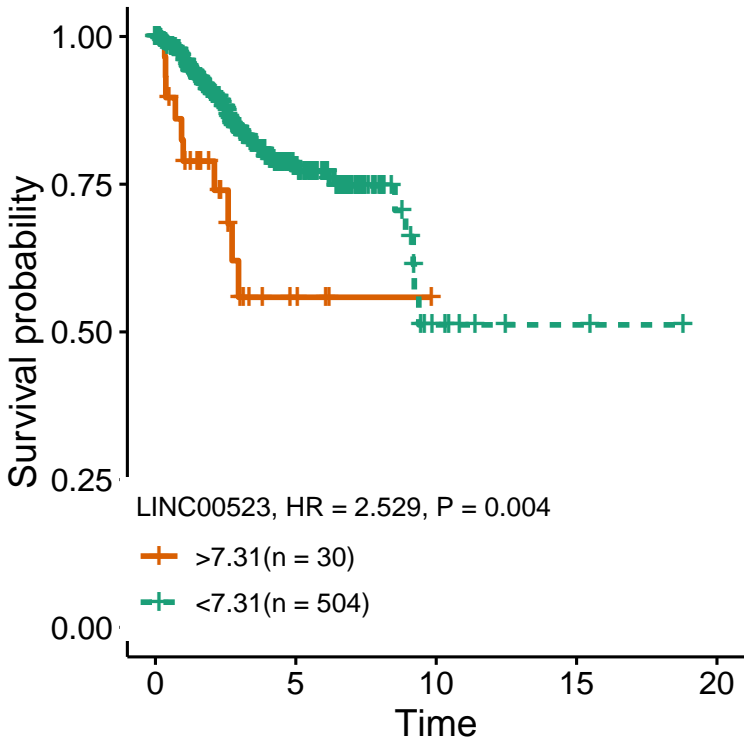

Supplement: Table S9 [file peerj-06-6091-s009.zip › Table S9/good_LINC00523.pdf]

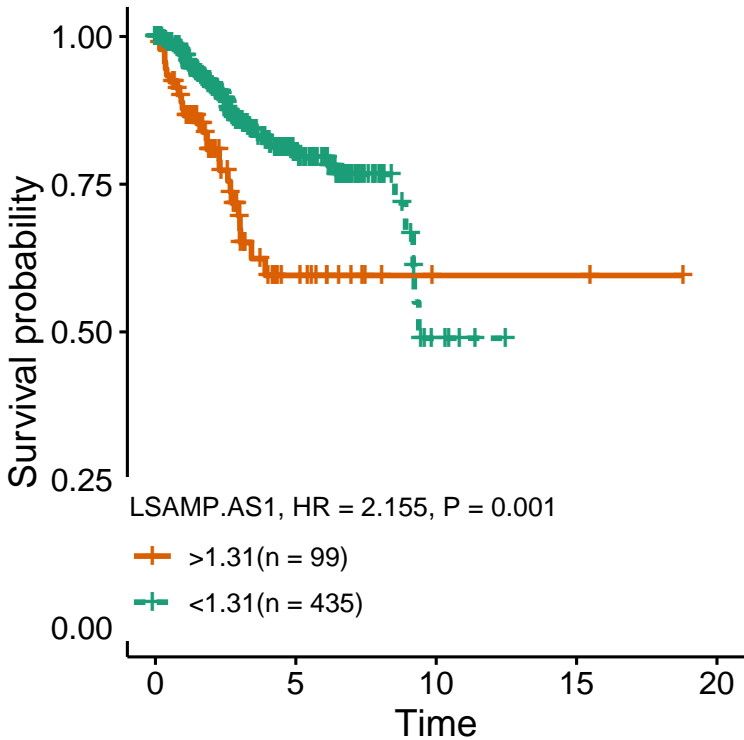

Supplement: Table S9 [file peerj-06-6091-s009.zip › Table S9/good_LSAMP.AS1.pdf]

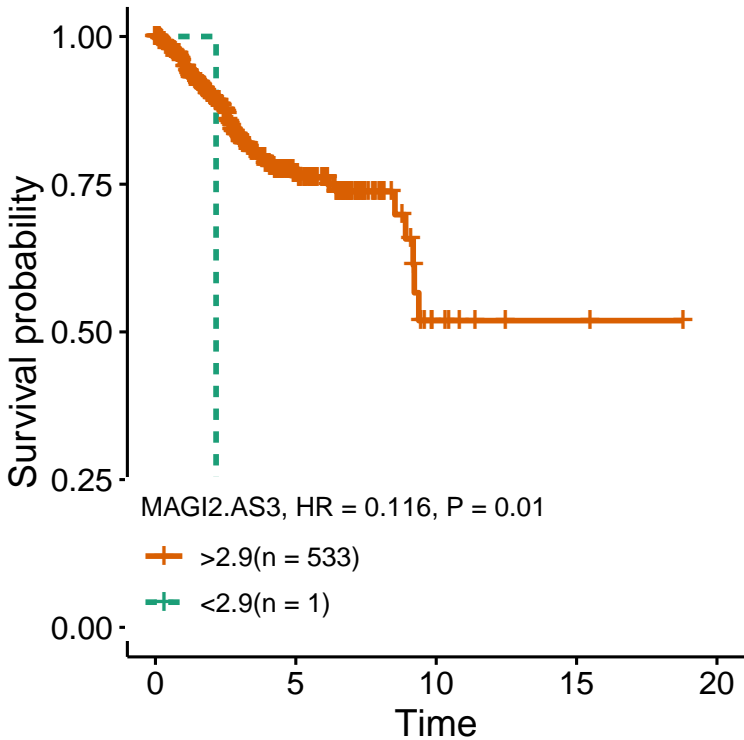

Supplement: Table S9 [file peerj-06-6091-s009.zip › Table S9/good_MAGI2.AS3.pdf]

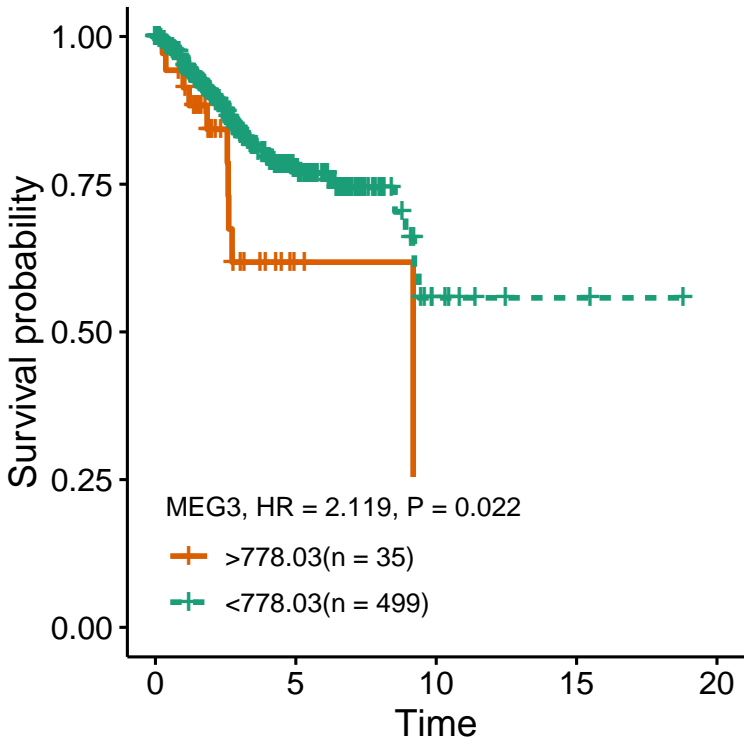

Supplement: Table S9 [file peerj-06-6091-s009.zip › Table S9/good_MEG3.pdf]

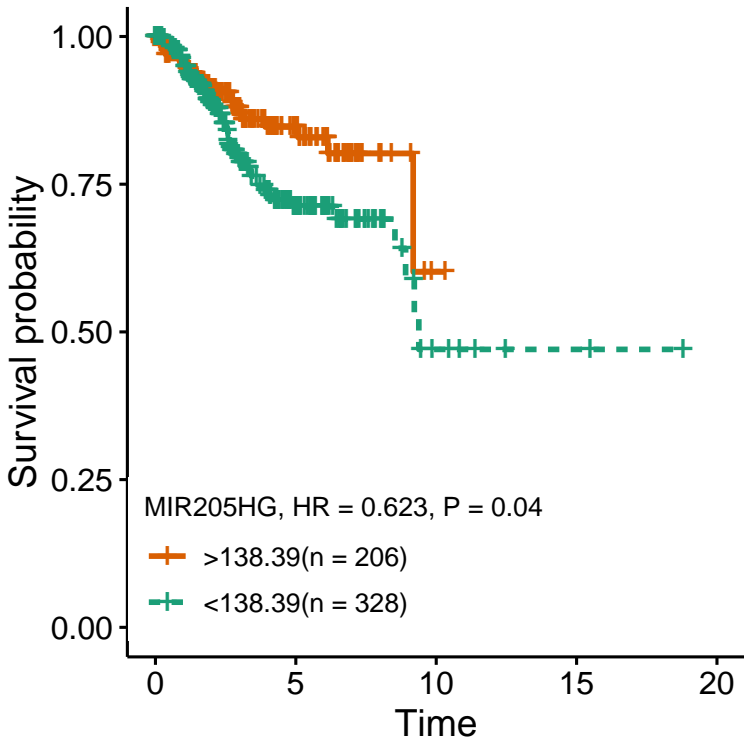

Supplement: Table S9 [file peerj-06-6091-s009.zip › Table S9/good_MIR205HG.pdf]

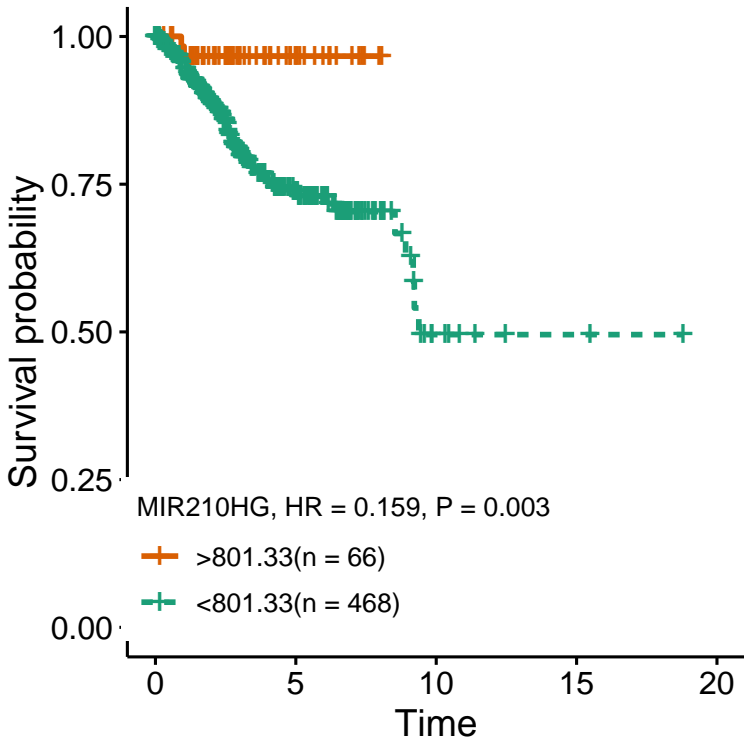

Supplement: Table S9 [file peerj-06-6091-s009.zip › Table S9/good_MIR210HG.pdf]

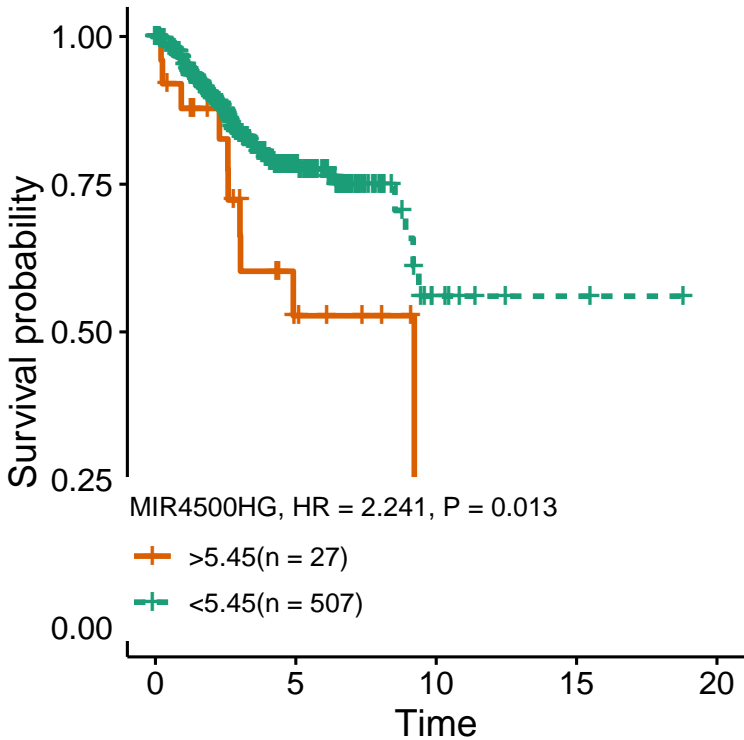

Supplement: Table S9 [file peerj-06-6091-s009.zip › Table S9/good_MIR4500HG.pdf]

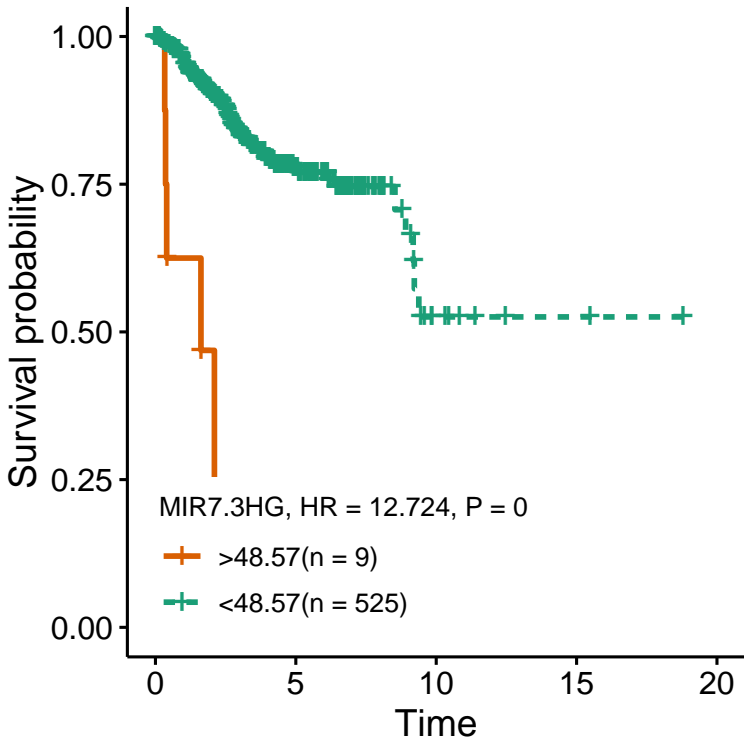

Supplement: Table S9 [file peerj-06-6091-s009.zip › Table S9/good_MIR7.3HG.pdf]

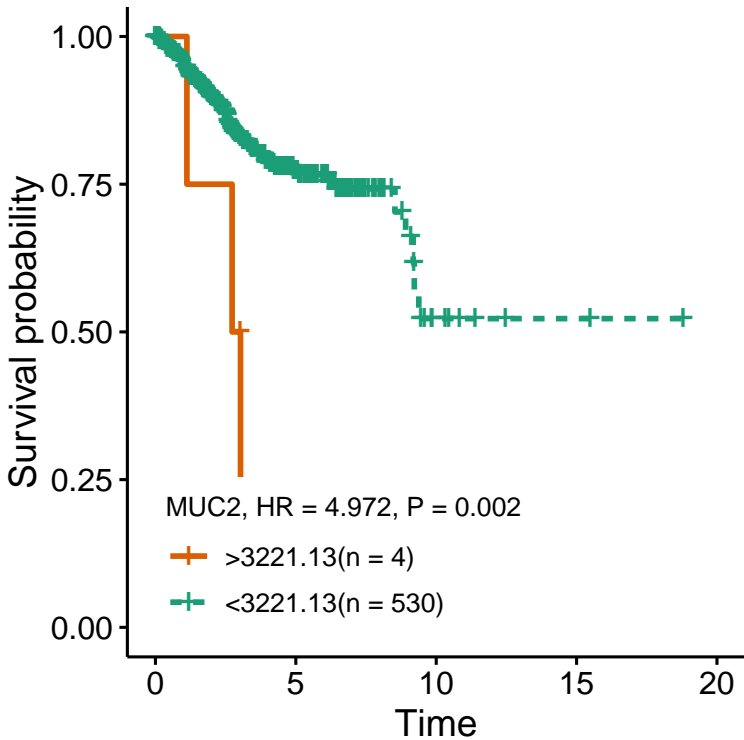

Supplement: Table S9 [file peerj-06-6091-s009.zip › Table S9/good_MUC2.pdf]

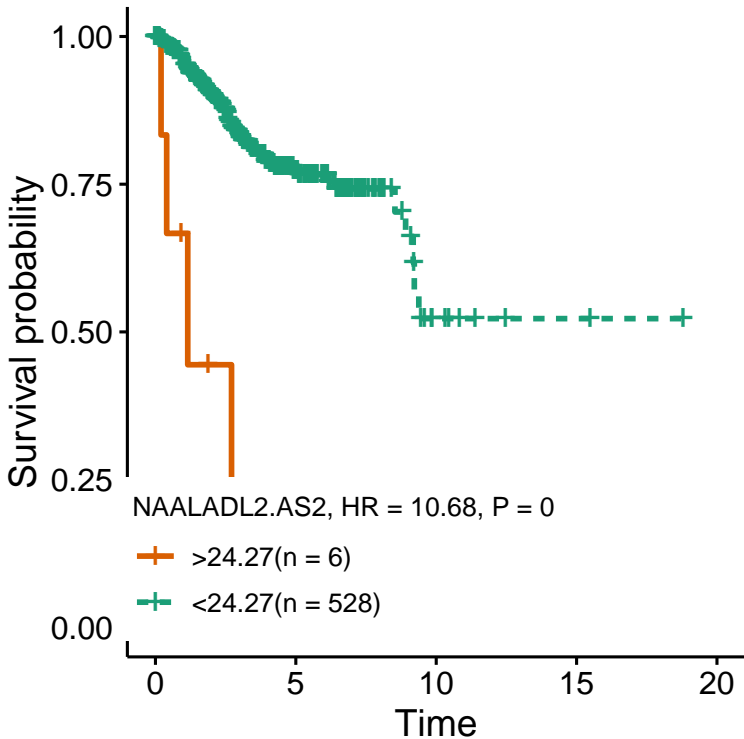

Supplement: Table S9 [file peerj-06-6091-s009.zip › Table S9/good_NAALADL2.AS2.pdf]

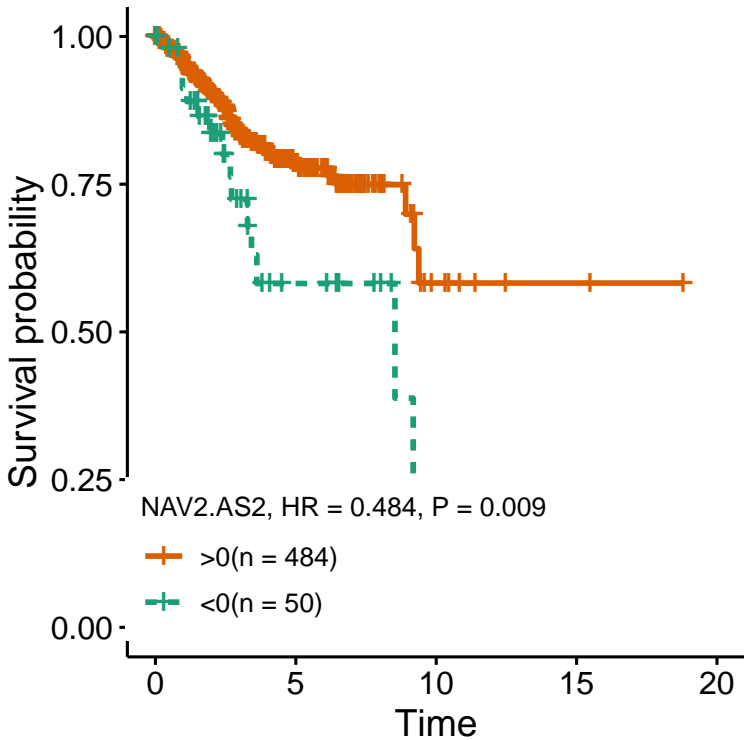

Supplement: Table S9 [file peerj-06-6091-s009.zip › Table S9/good_NAV2.AS2.pdf]

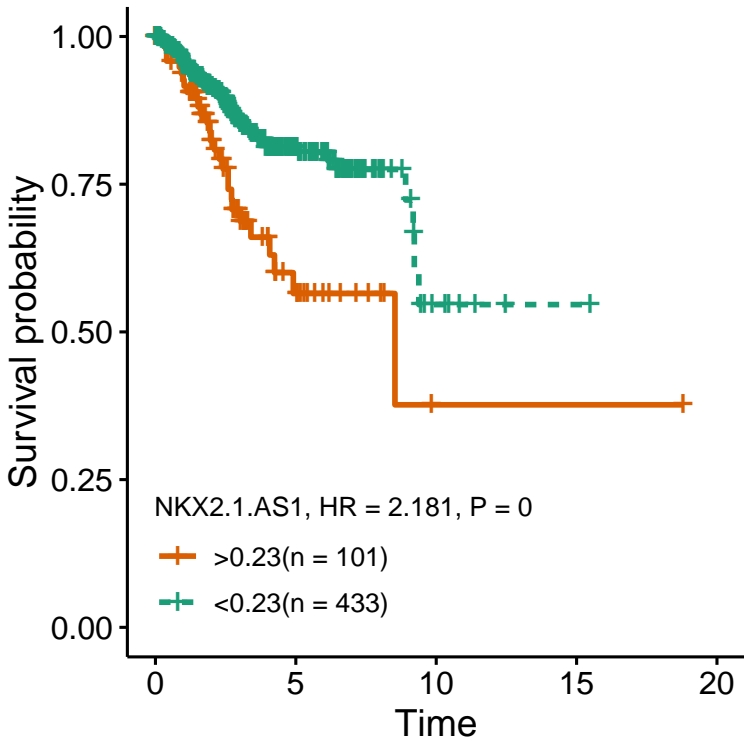

Supplement: Table S9 [file peerj-06-6091-s009.zip › Table S9/good_NKX2.1.AS1.pdf]

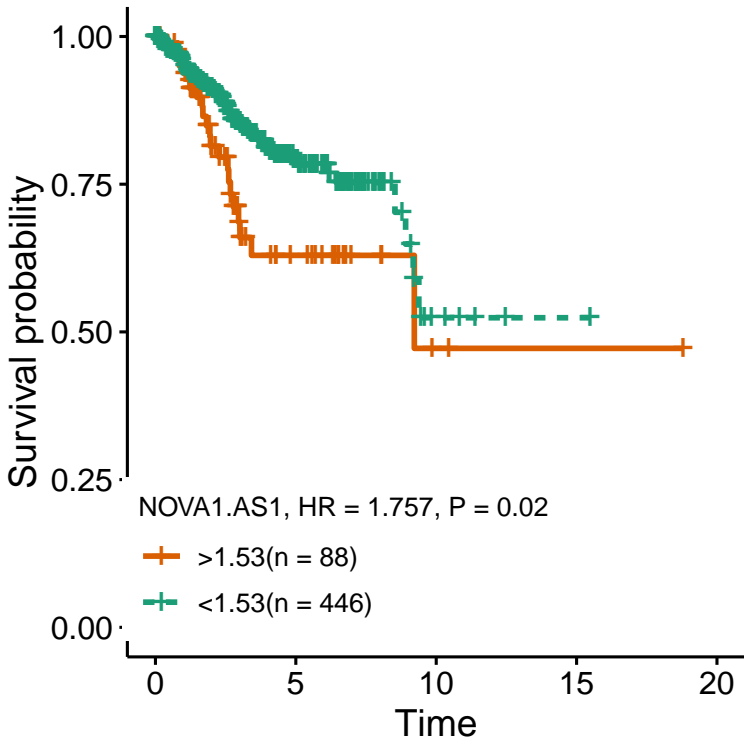

Supplement: Table S9 [file peerj-06-6091-s009.zip › Table S9/good_NOVA1.AS1.pdf]

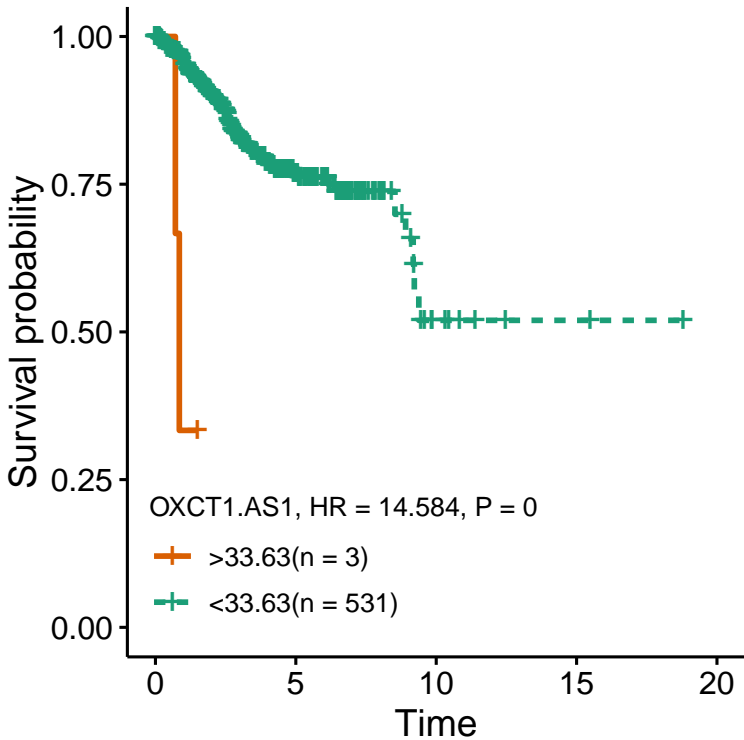

Supplement: Table S9 [file peerj-06-6091-s009.zip › Table S9/good_OXCT1.AS1.pdf]

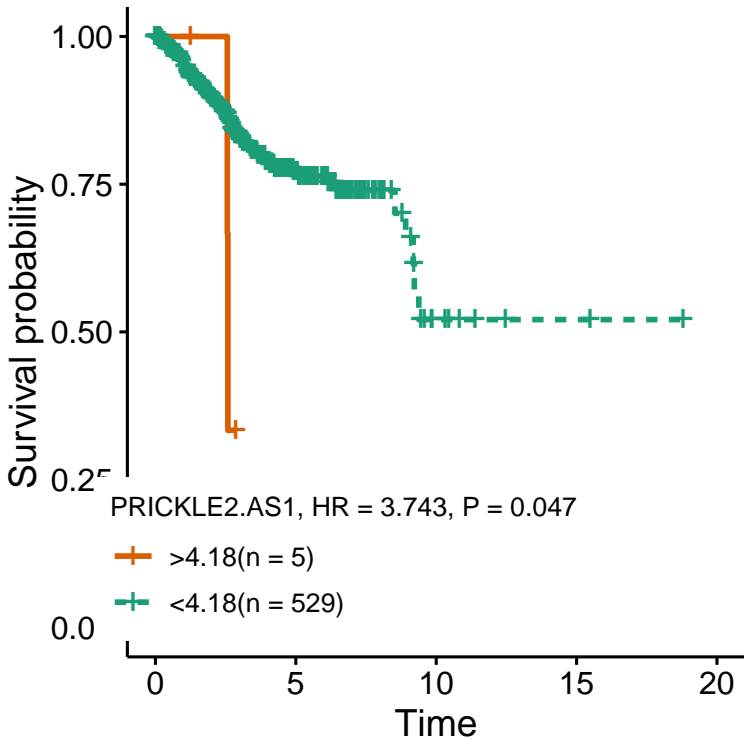

Supplement: Table S9 [file peerj-06-6091-s009.zip › Table S9/good_PRICKLE2.AS1.pdf]

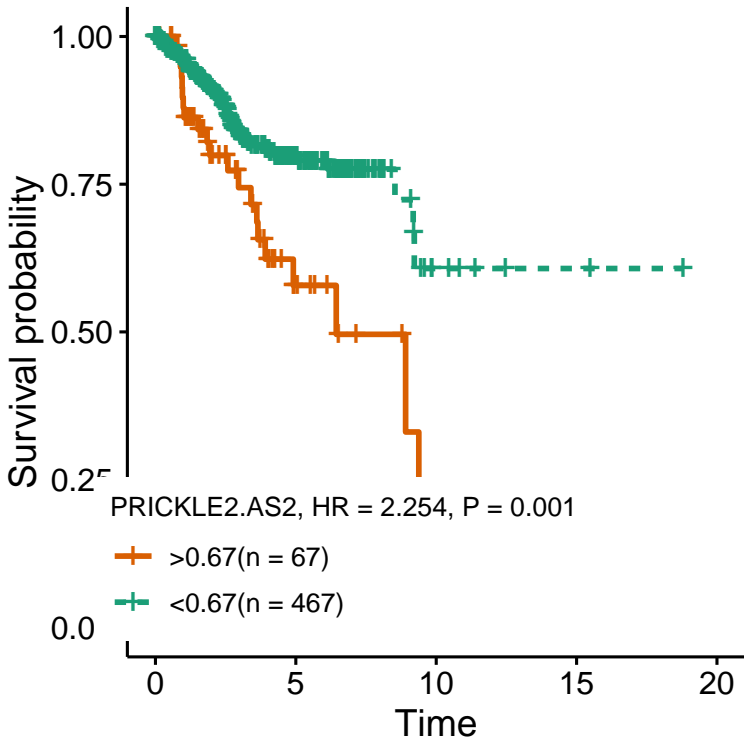

Supplement: Table S9 [file peerj-06-6091-s009.zip › Table S9/good_PRICKLE2.AS2.pdf]

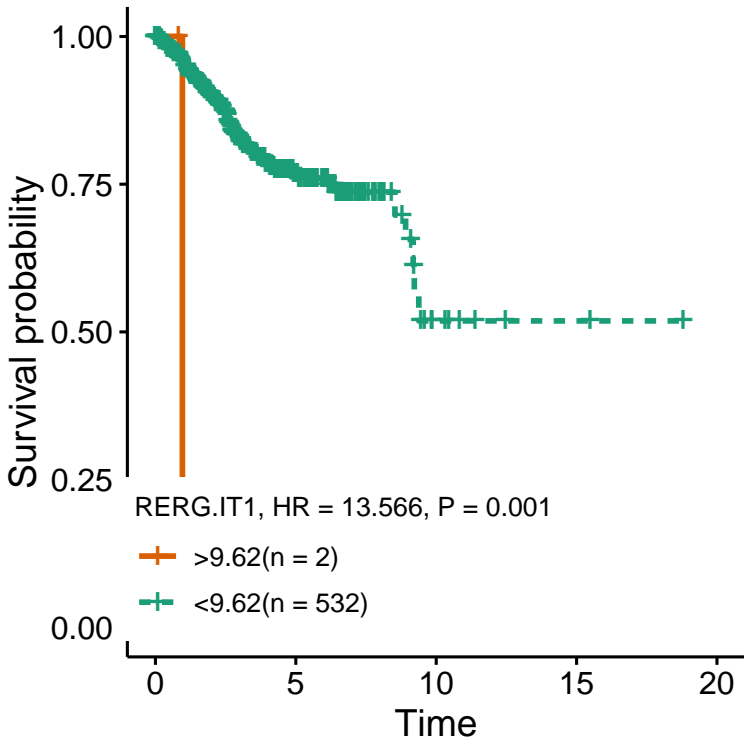

Supplement: Table S9 [file peerj-06-6091-s009.zip › Table S9/good_RERG.IT1.pdf]

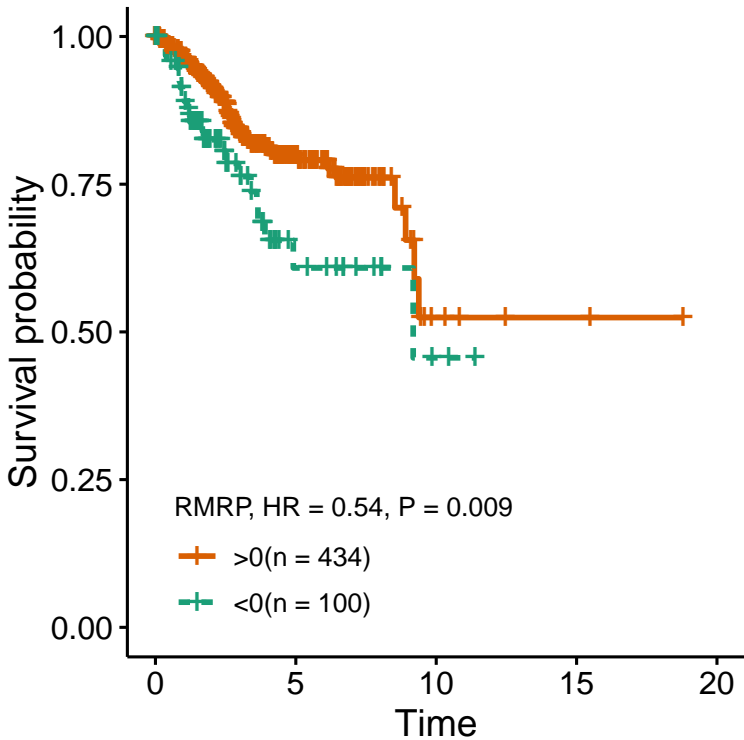

Supplement: Table S9 [file peerj-06-6091-s009.zip › Table S9/good_RMRP.pdf]

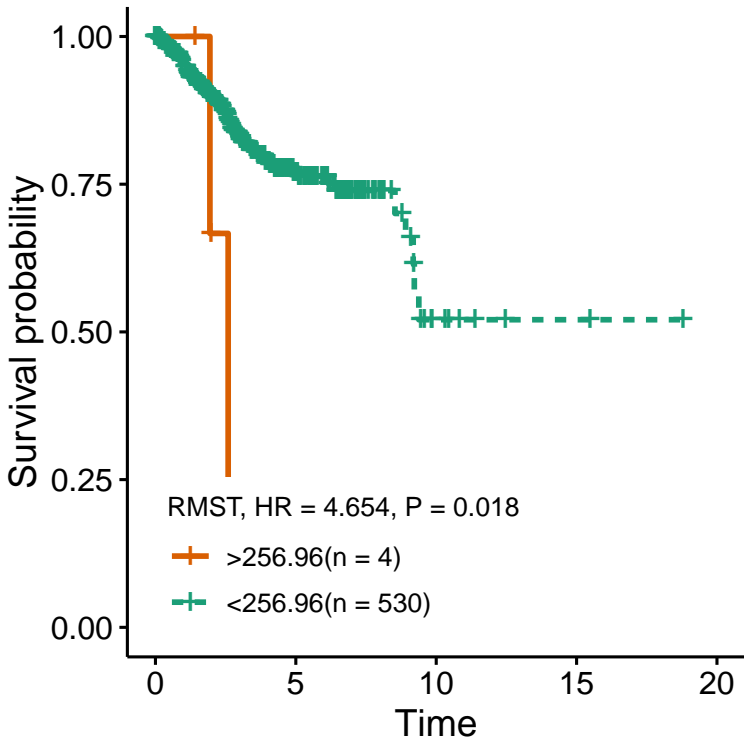

Supplement: Table S9 [file peerj-06-6091-s009.zip › Table S9/good_RMST.pdf]

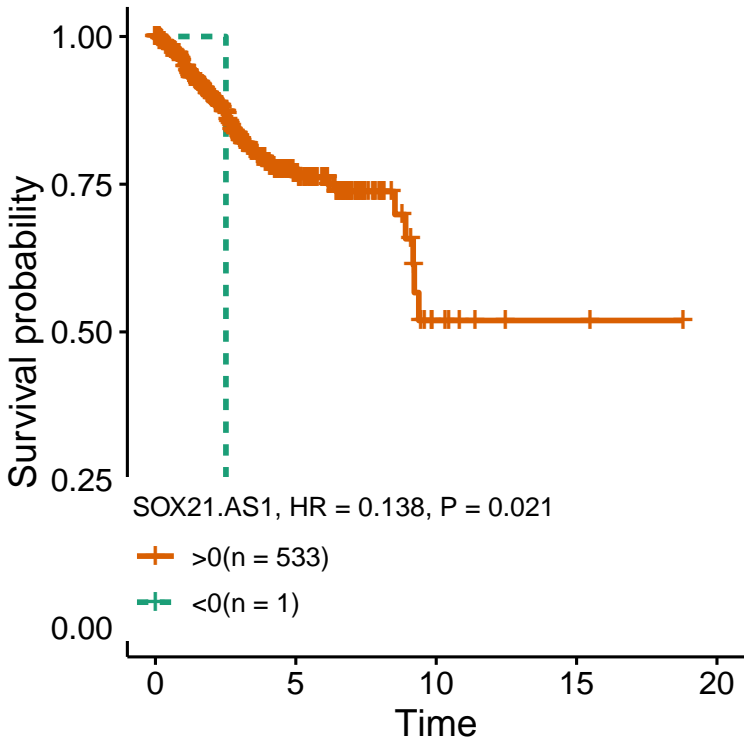

Supplement: Table S9 [file peerj-06-6091-s009.zip › Table S9/good_SOX21.AS1.pdf]

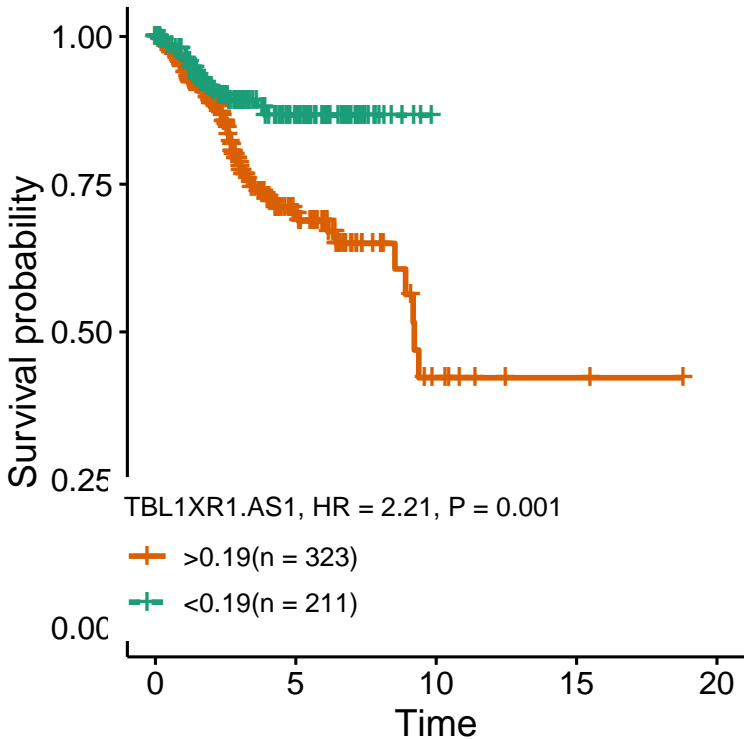

Supplement: Table S9 [file peerj-06-6091-s009.zip › Table S9/good_TBL1XR1.AS1.pdf]

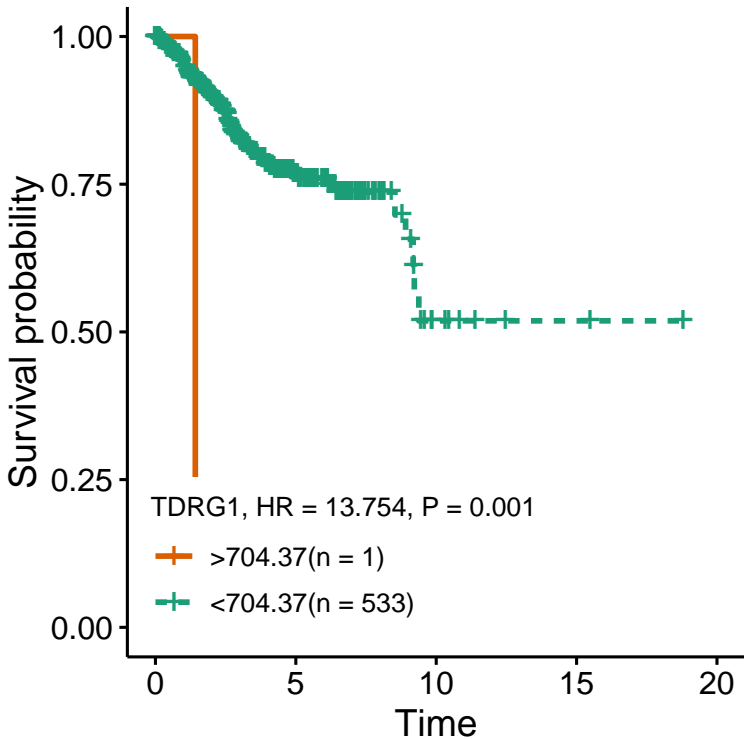

Supplement: Table S9 [file peerj-06-6091-s009.zip › Table S9/good_TDRG1.pdf]

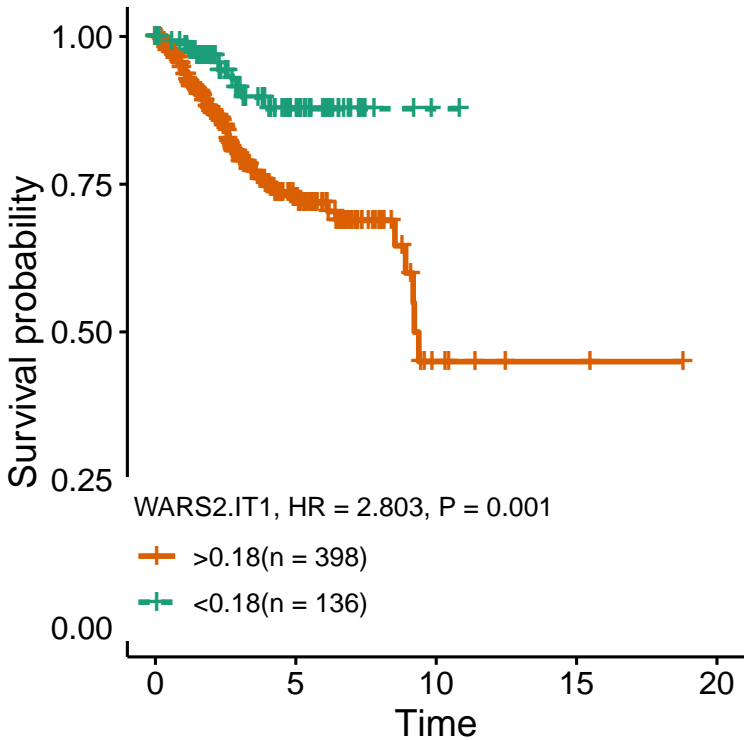

Supplement: Table S9 [file peerj-06-6091-s009.zip › Table S9/good_WARS2.IT1.pdf]

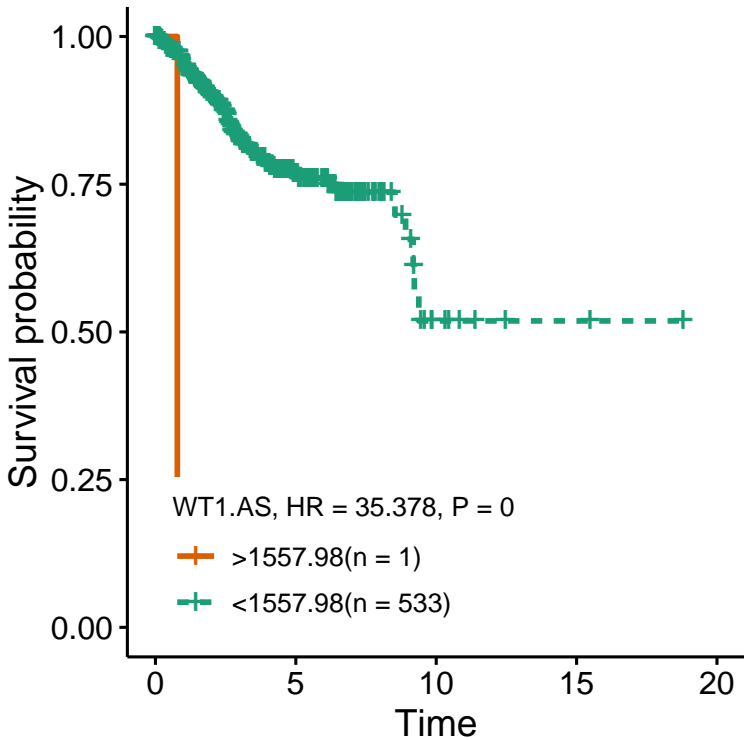

Supplement: Table S9 [file peerj-06-6091-s009.zip › Table S9/good_WT1.AS.pdf]

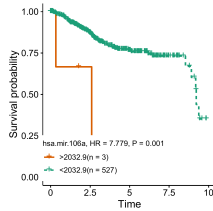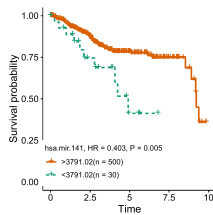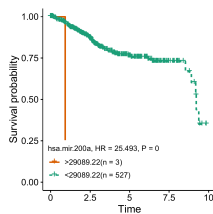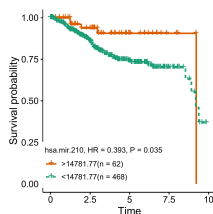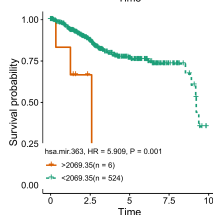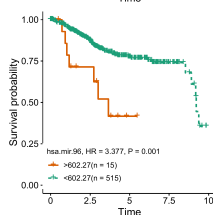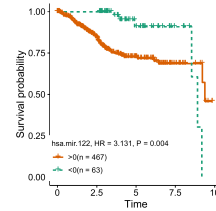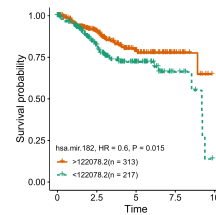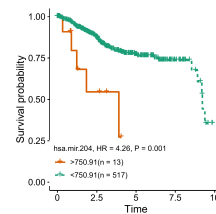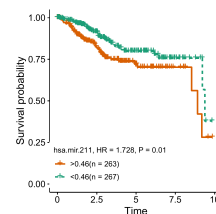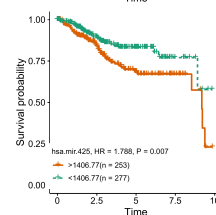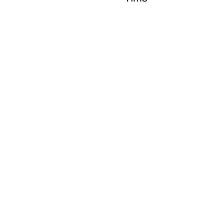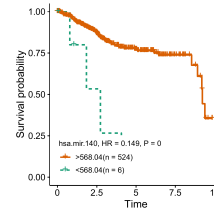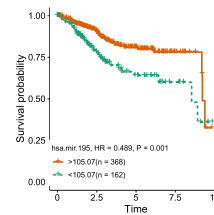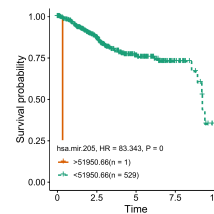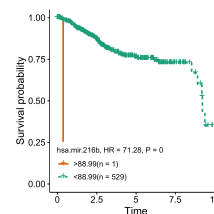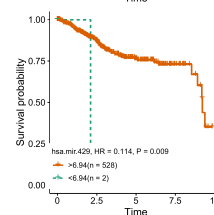

Supplement: Table S10 [file peerj-06-6091-s010.zip › Table S10/bestSep_good.pdf]

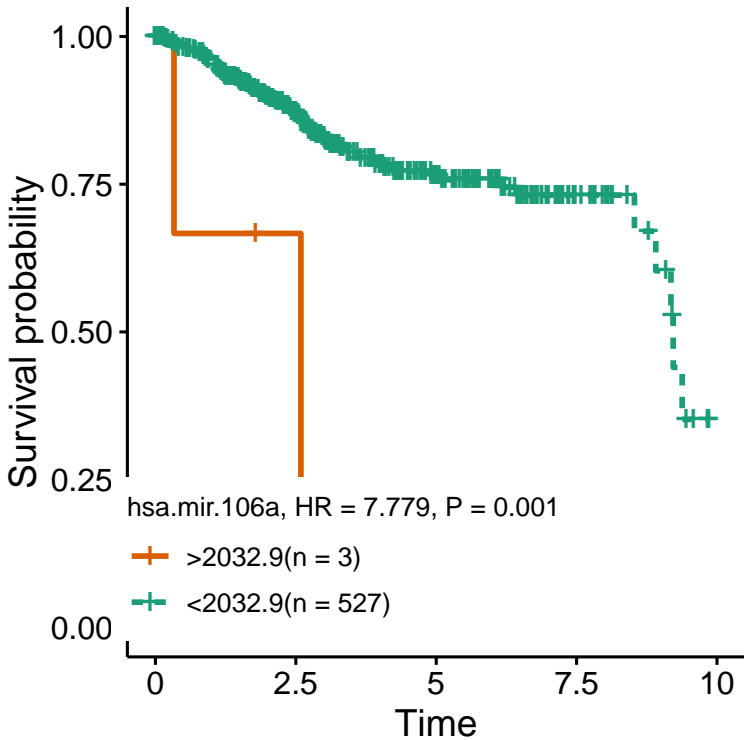

Supplement: Table S10 [file peerj-06-6091-s010.zip › Table S10/good_hsa.mir.106a.pdf]

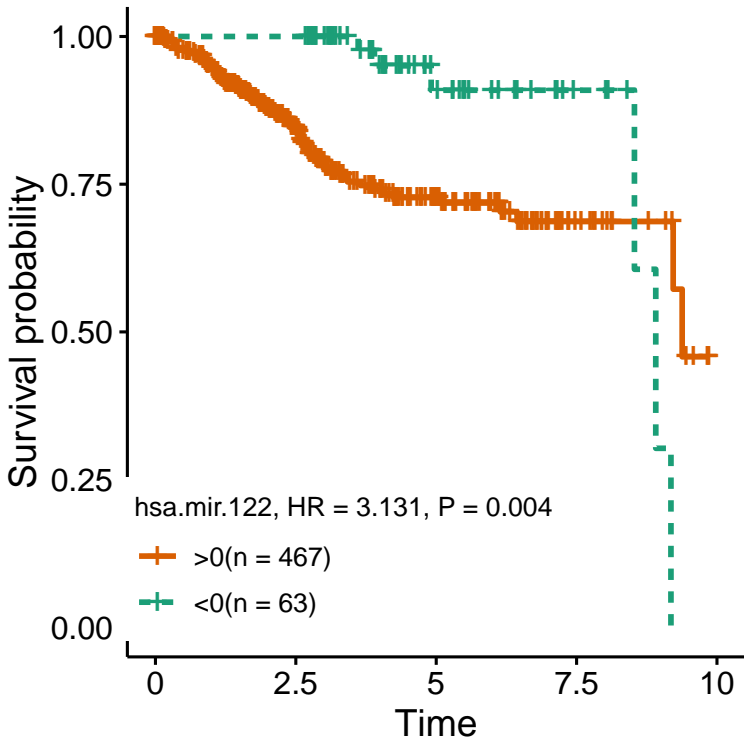

Supplement: Table S10 [file peerj-06-6091-s010.zip › Table S10/good_hsa.mir.122.pdf]

Survival probability

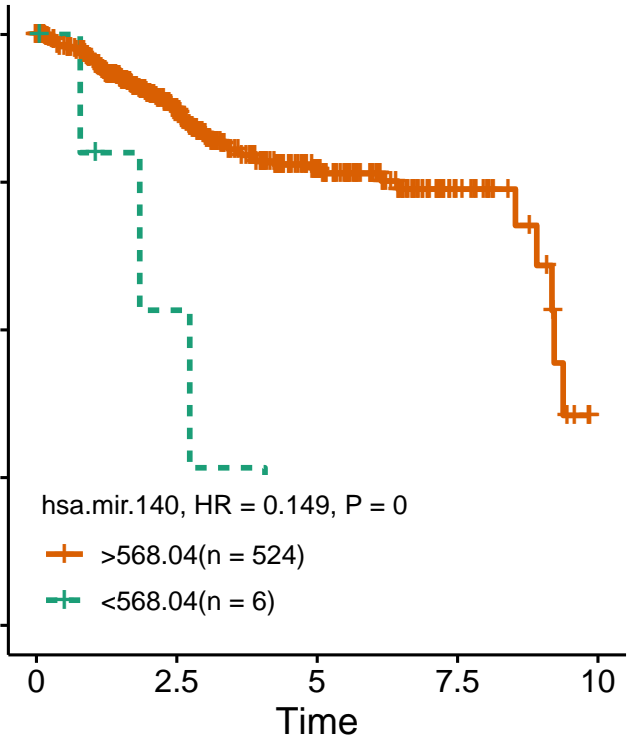

Supplement: Table S10 [file peerj-06-6091-s010.zip › Table S10/good_hsa.mir.140.pdf]

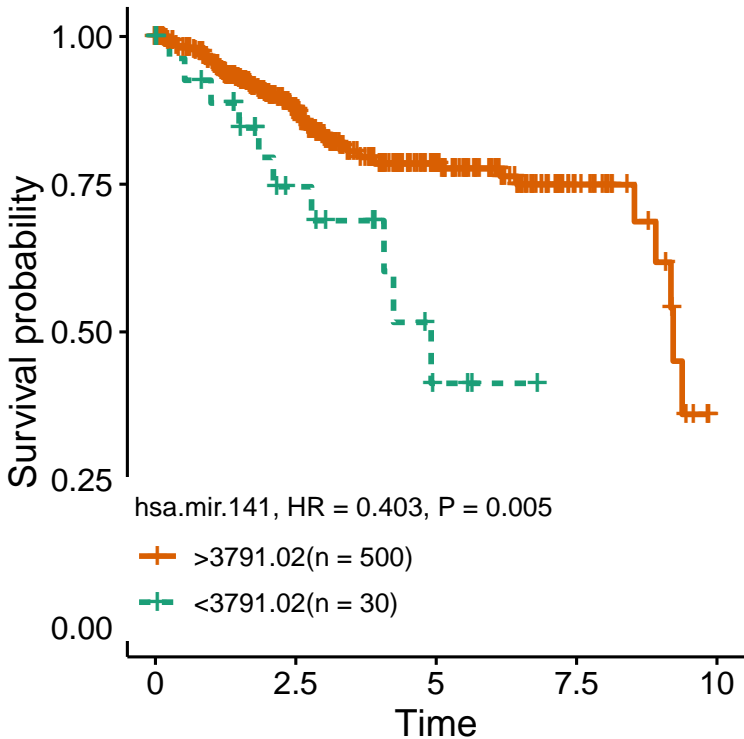

Supplement: Table S10 [file peerj-06-6091-s010.zip › Table S10/good_hsa.mir.141.pdf]

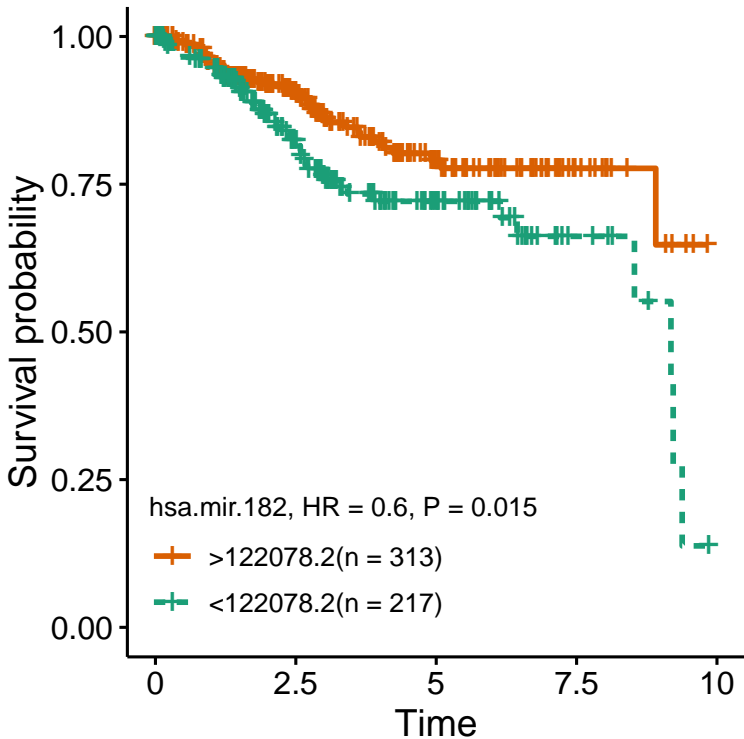

Supplement: Table S10 [file peerj-06-6091-s010.zip › Table S10/good_hsa.mir.182.pdf]

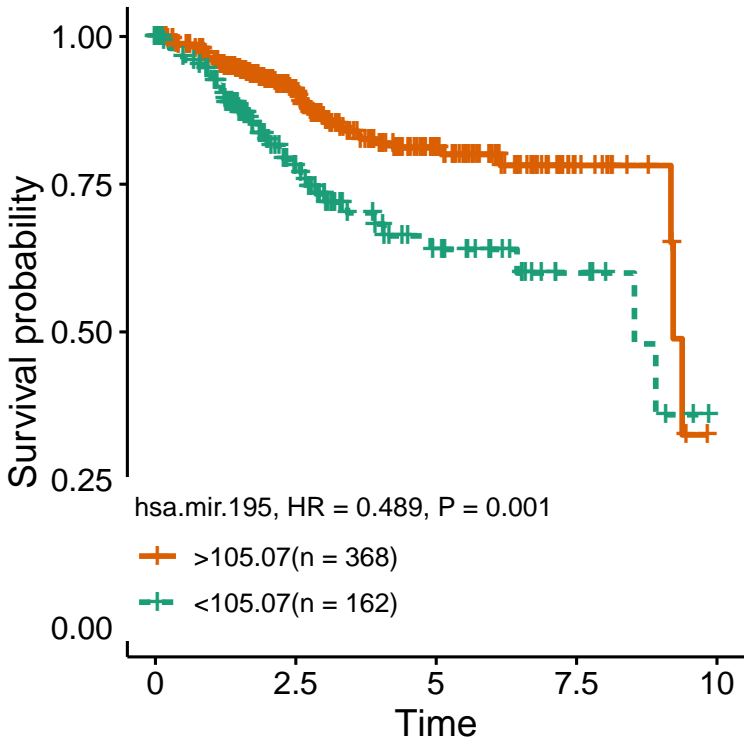

Supplement: Table S10 [file peerj-06-6091-s010.zip › Table S10/good_hsa.mir.195.pdf]
