# Supplementary figures and images for: Screening of prognostic biomarkers for endometrial carcinoma based on a ceRNA network (part 2 of 2)
Source: PeerJ. 2018 Dec 10;6:e6091. doi: 10.7717/peerj.6091 (PMC6292375; doi:10.7717/peerj.6091)

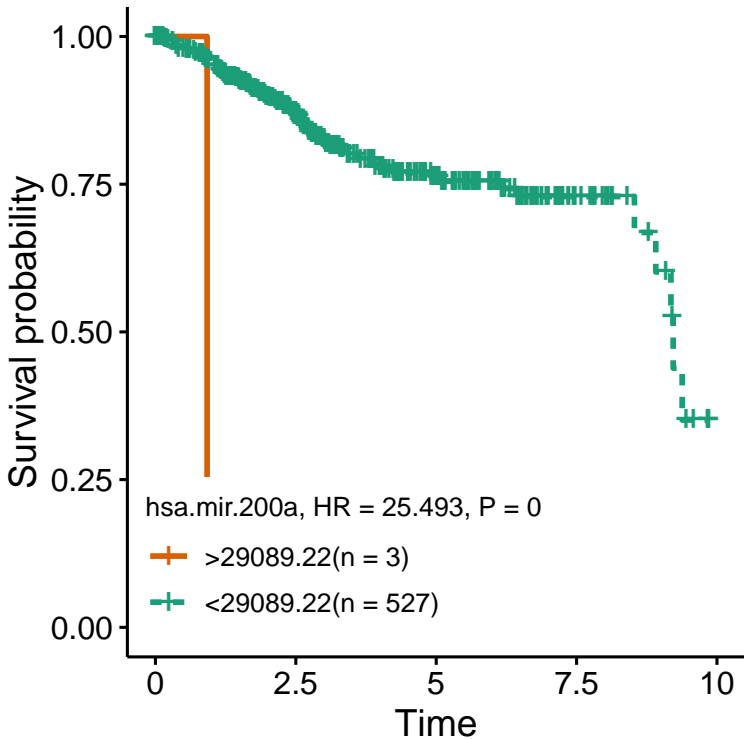

Supplement: Table S10 [file peerj-06-6091-s010.zip › Table S10/good_hsa.mir.200a.pdf]

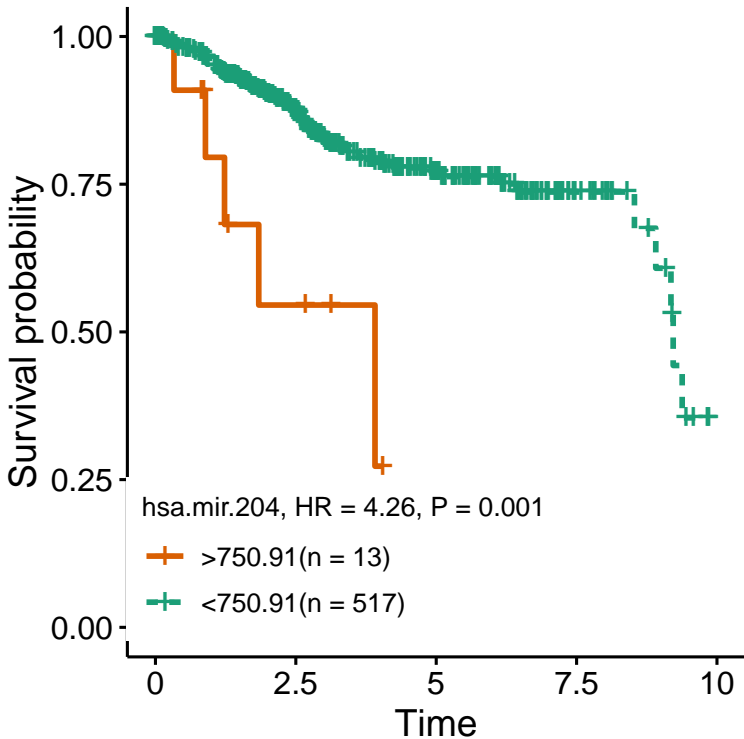

Supplement: Table S10 [file peerj-06-6091-s010.zip › Table S10/good_hsa.mir.204.pdf]

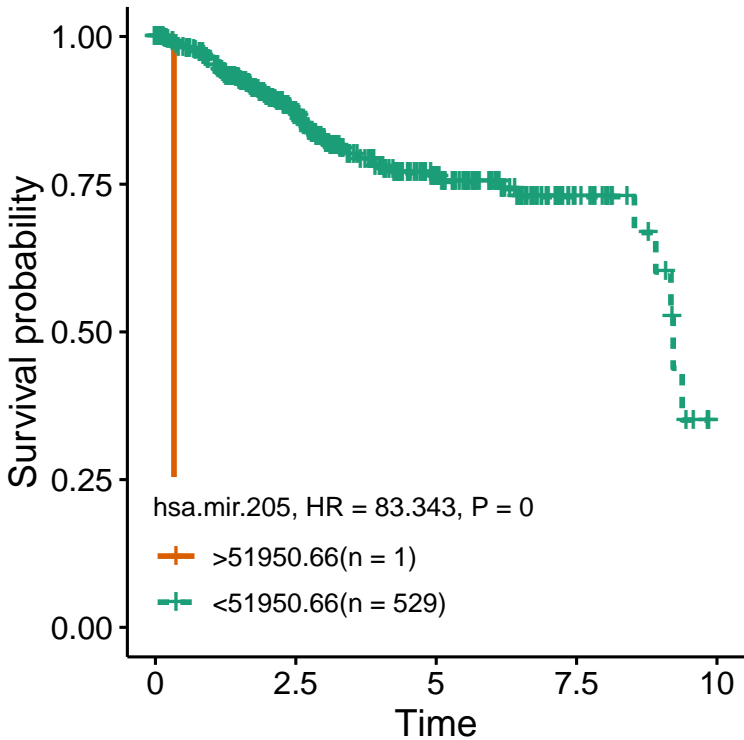

Supplement: Table S10 [file peerj-06-6091-s010.zip › Table S10/good_hsa.mir.205.pdf]

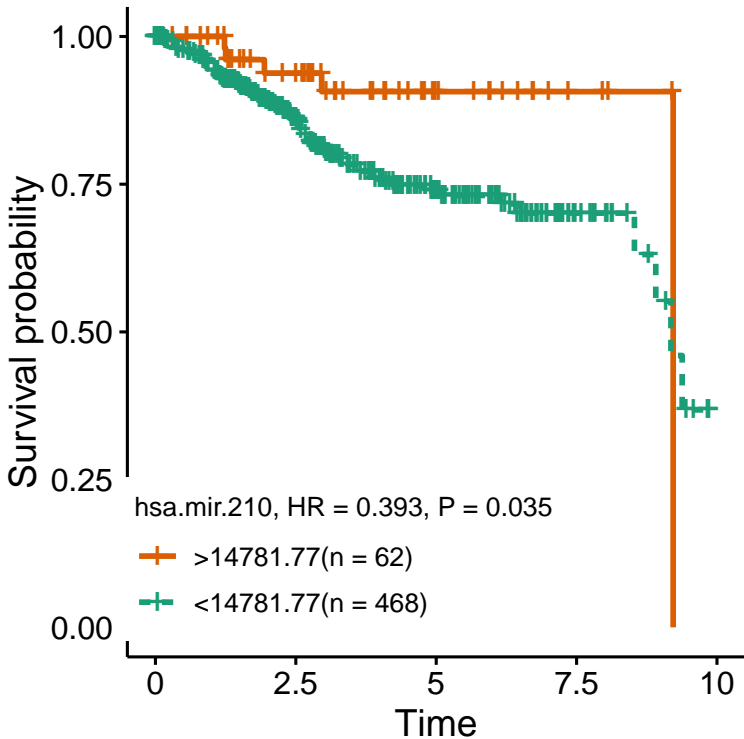

Supplement: Table S10 [file peerj-06-6091-s010.zip › Table S10/good_hsa.mir.210.pdf]

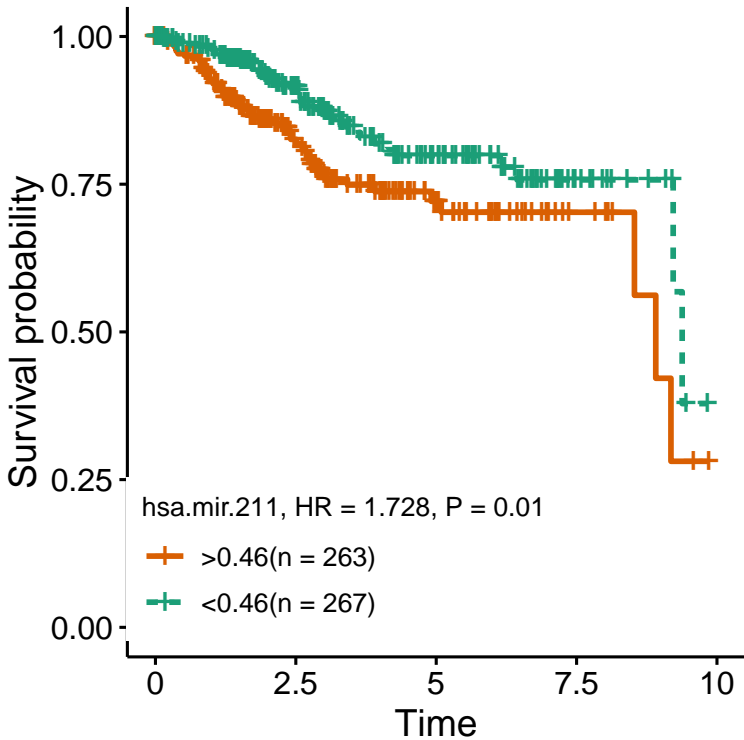

Supplement: Table S10 [file peerj-06-6091-s010.zip › Table S10/good_hsa.mir.211.pdf]

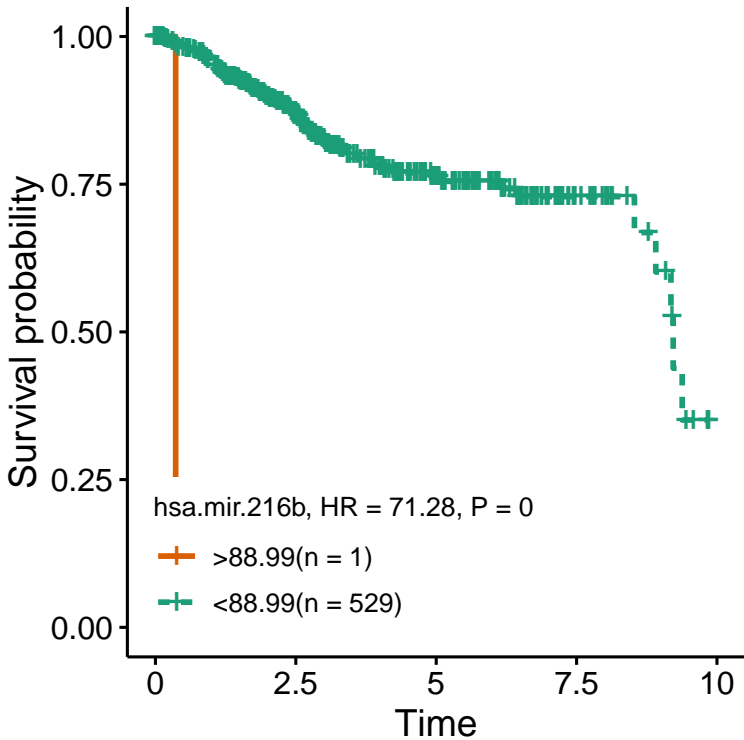

Supplement: Table S10 [file peerj-06-6091-s010.zip › Table S10/good_hsa.mir.216b.pdf]

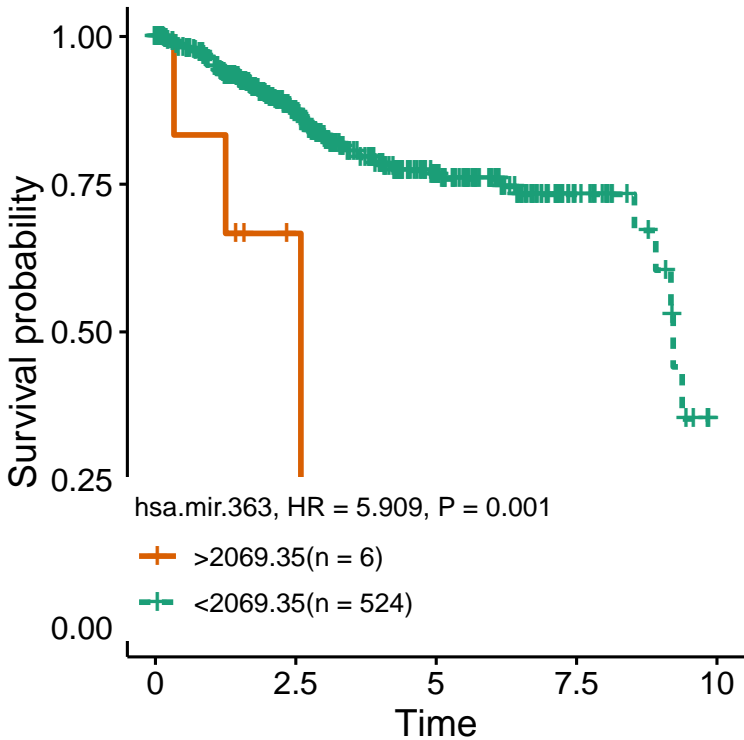

Supplement: Table S10 [file peerj-06-6091-s010.zip › Table S10/good_hsa.mir.363.pdf]

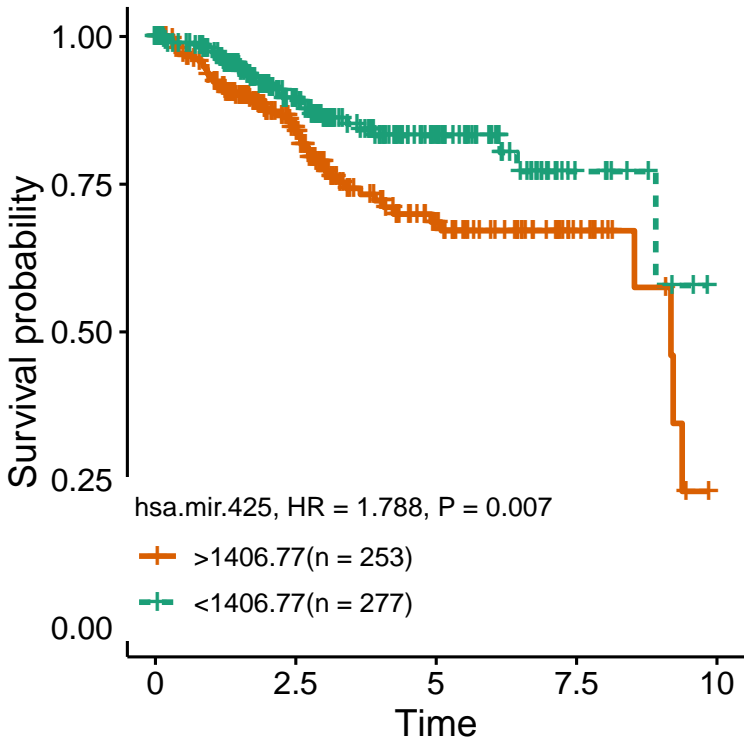

Supplement: Table S10 [file peerj-06-6091-s010.zip › Table S10/good_hsa.mir.425.pdf]

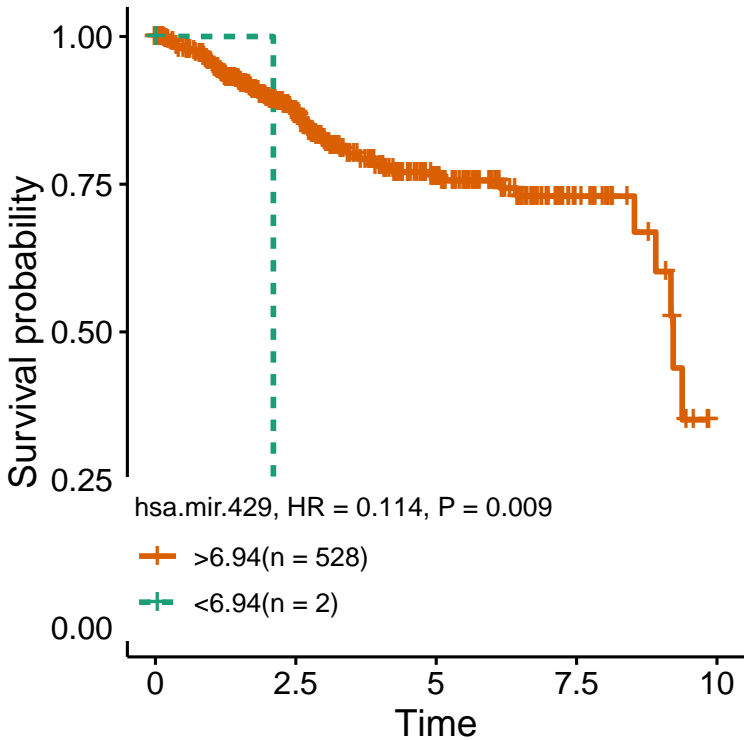

Supplement: Table S10 [file peerj-06-6091-s010.zip › Table S10/good_hsa.mir.429.pdf]

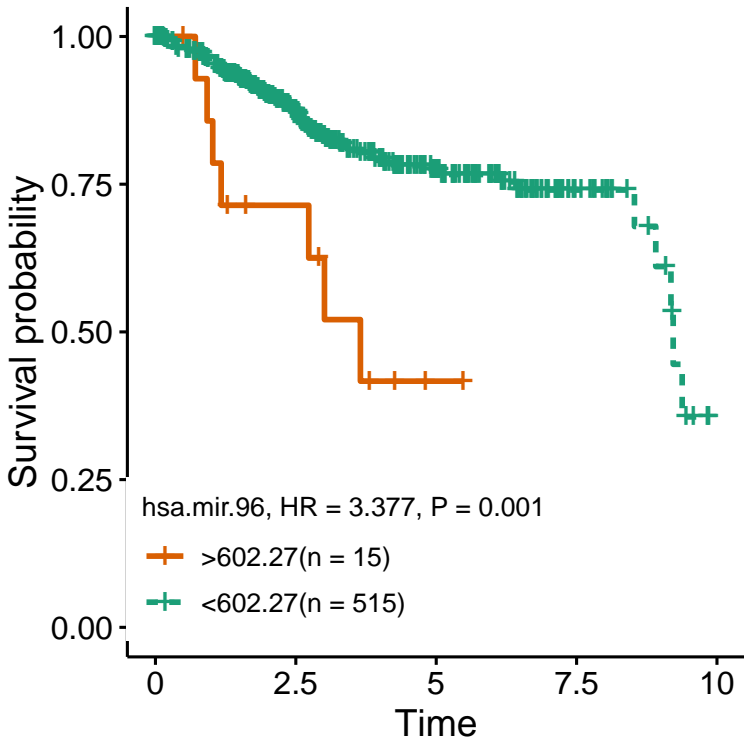

Supplement: Table S10 [file peerj-06-6091-s010.zip › Table S10/good_hsa.mir.96.pdf]

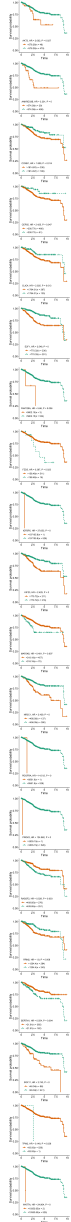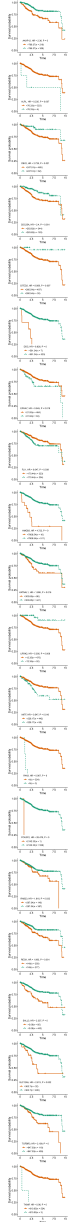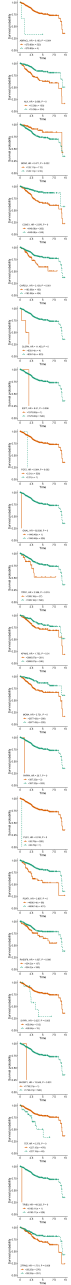

Supplement: Table S11 [file peerj-06-6091-s011.zip › Table S11/bestSep_good.pdf]

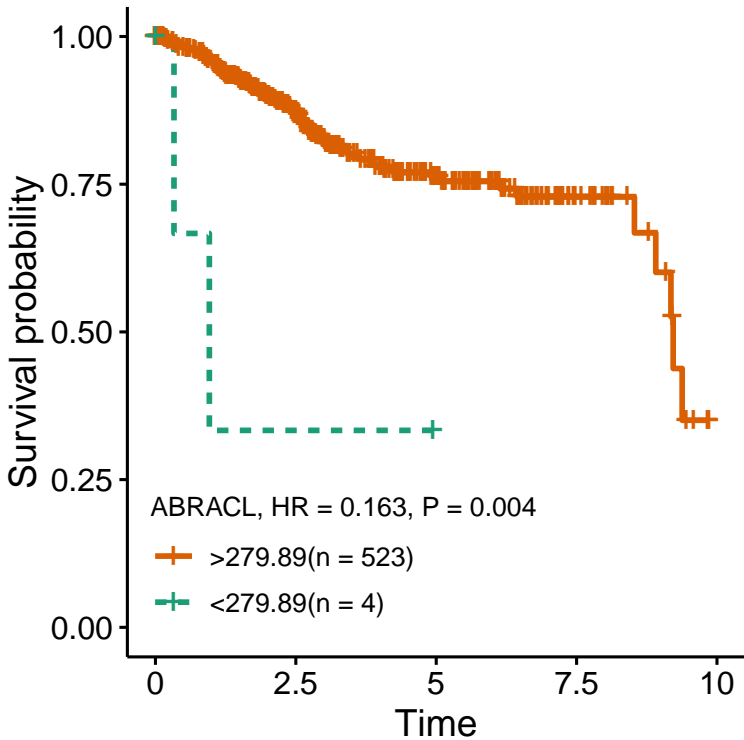

Supplement: Table S11 [file peerj-06-6091-s011.zip › Table S11/good_ABRACL.pdf]

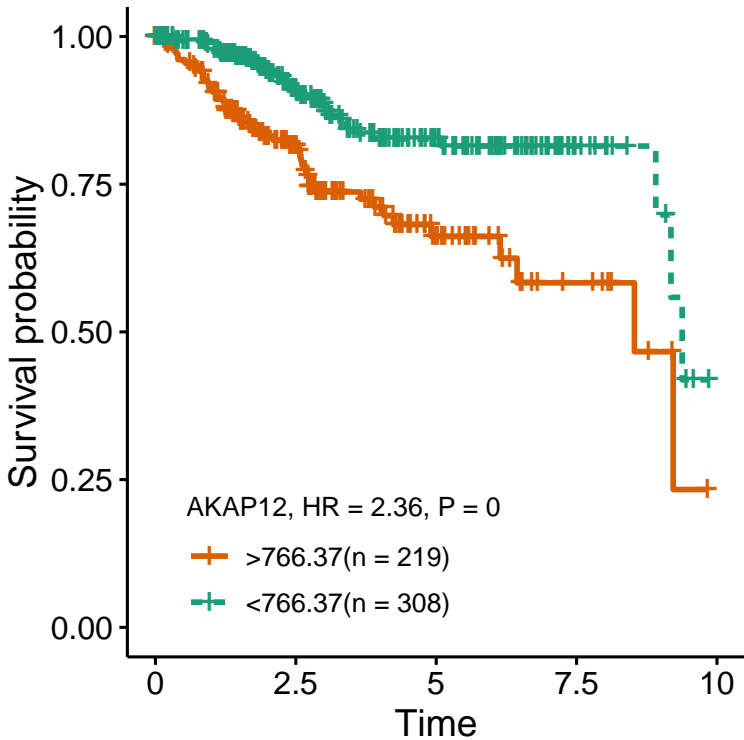

Supplement: Table S11 [file peerj-06-6091-s011.zip › Table S11/good_AKAP12.pdf]

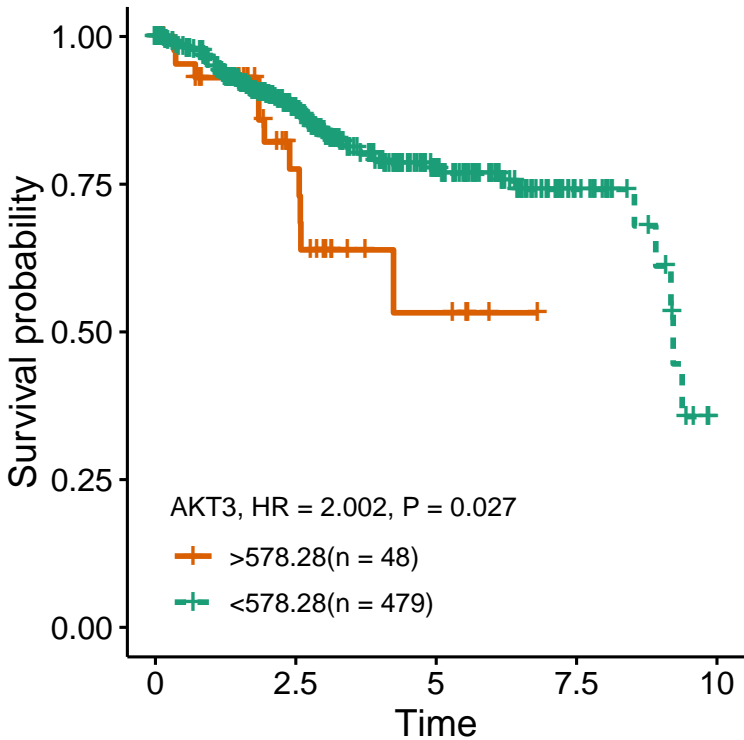

Supplement: Table S11 [file peerj-06-6091-s011.zip › Table S11/good_AKT3.pdf]

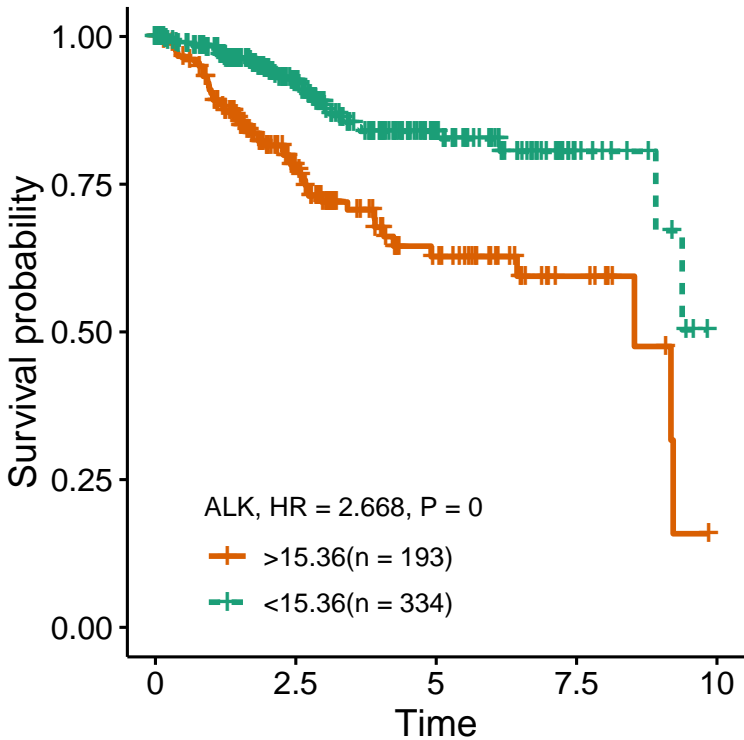

Supplement: Table S11 [file peerj-06-6091-s011.zip › Table S11/good_ALK.pdf]

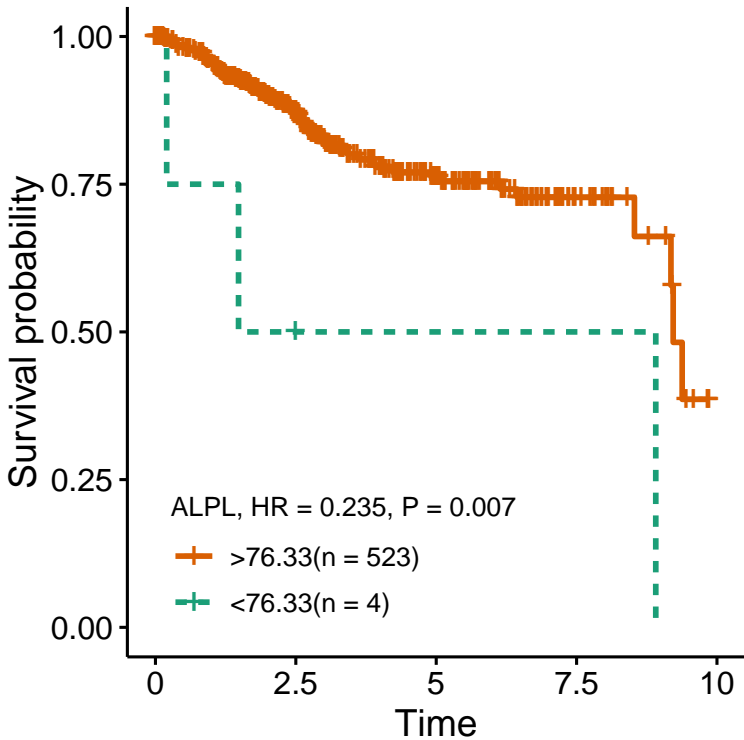

Supplement: Table S11 [file peerj-06-6091-s011.zip › Table S11/good_ALPL.pdf]

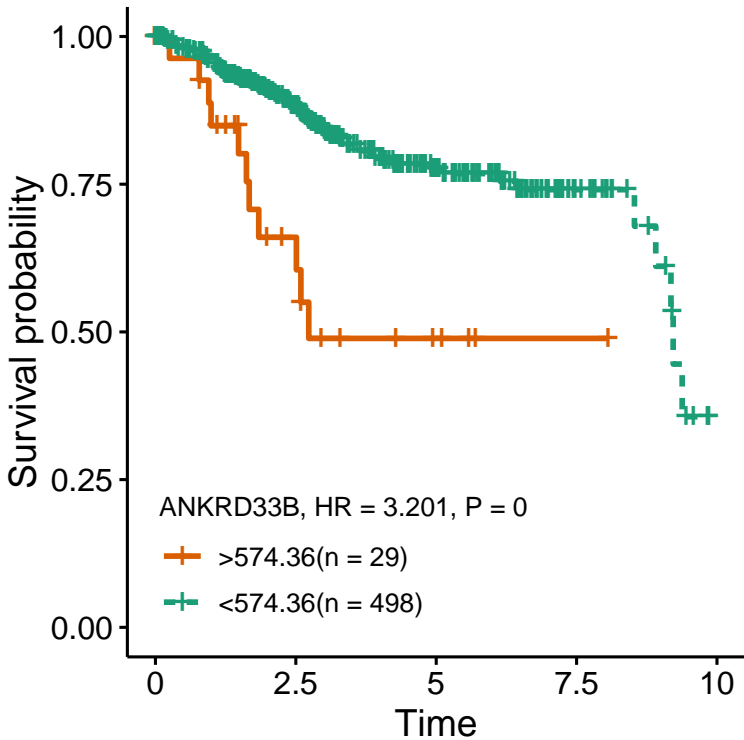

Supplement: Table S11 [file peerj-06-6091-s011.zip › Table S11/good_ANKRD33B.pdf]

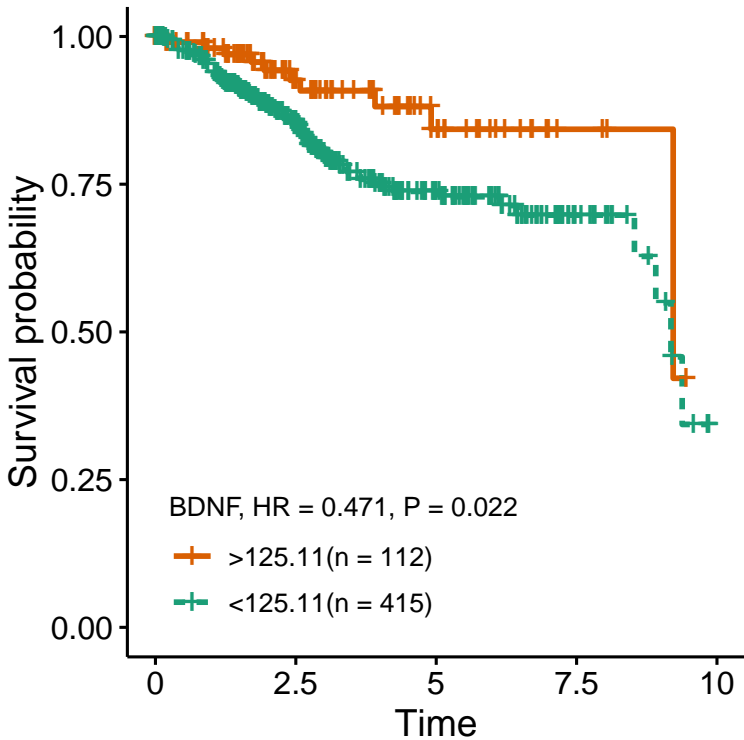

Supplement: Table S11 [file peerj-06-6091-s011.zip › Table S11/good_BDNF.pdf]

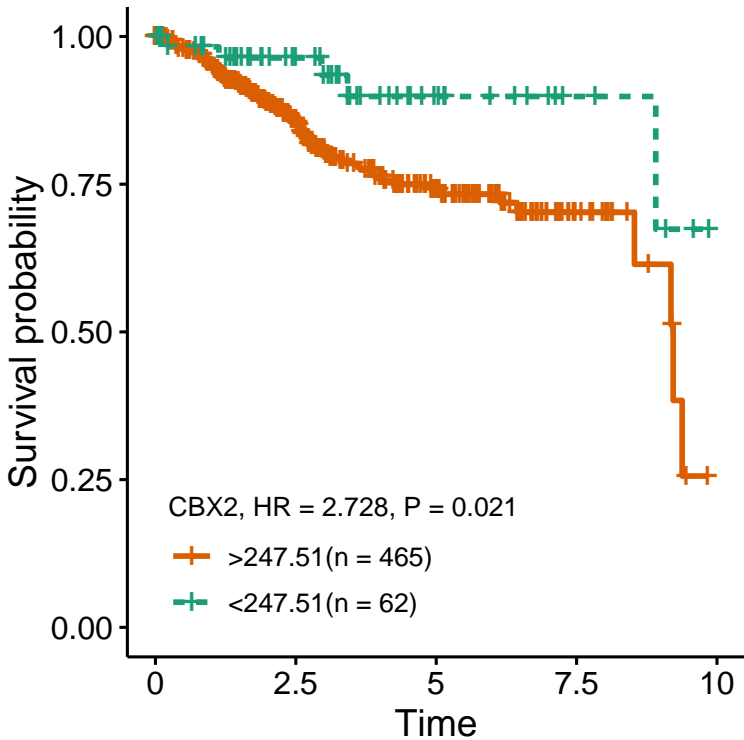

Supplement: Table S11 [file peerj-06-6091-s011.zip › Table S11/good_CBX2.pdf]

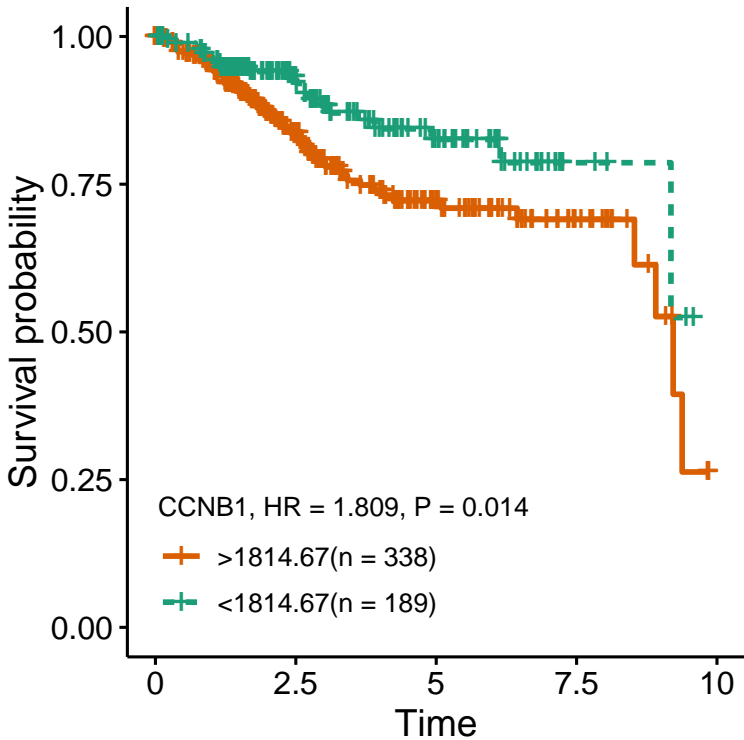

Supplement: Table S11 [file peerj-06-6091-s011.zip › Table S11/good_CCNB1.pdf]

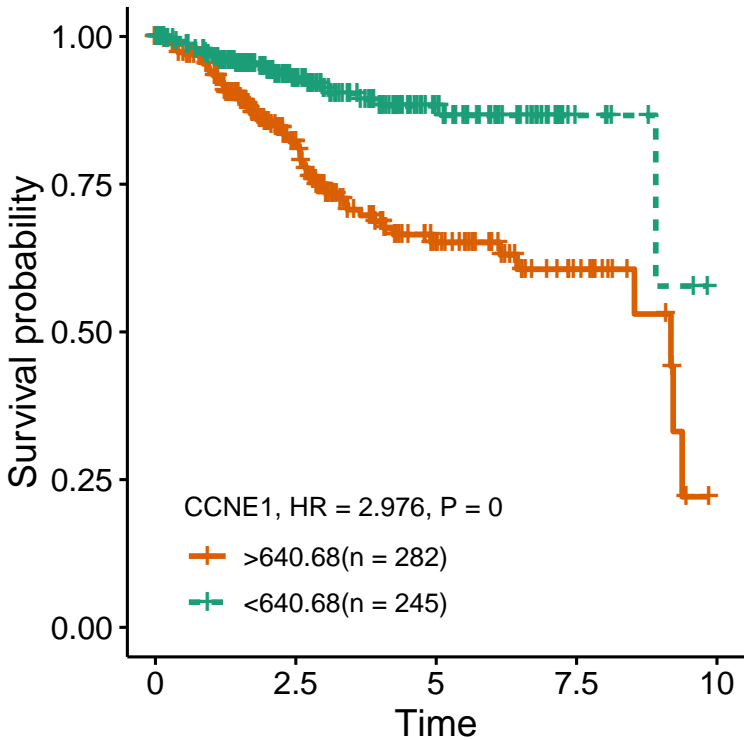

Supplement: Table S11 [file peerj-06-6091-s011.zip › Table S11/good_CCNE1.pdf]

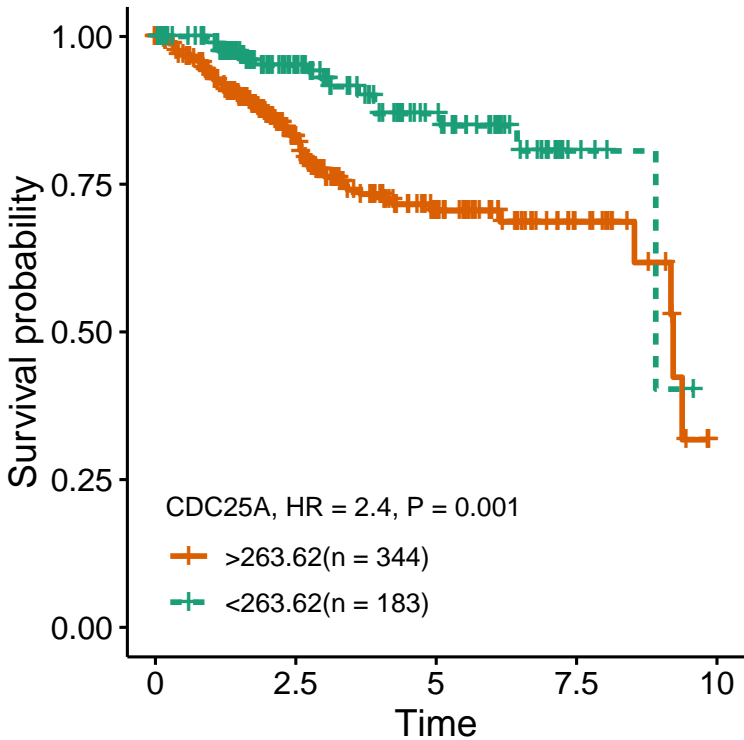

Supplement: Table S11 [file peerj-06-6091-s011.zip › Table S11/good_CDC25A.pdf]

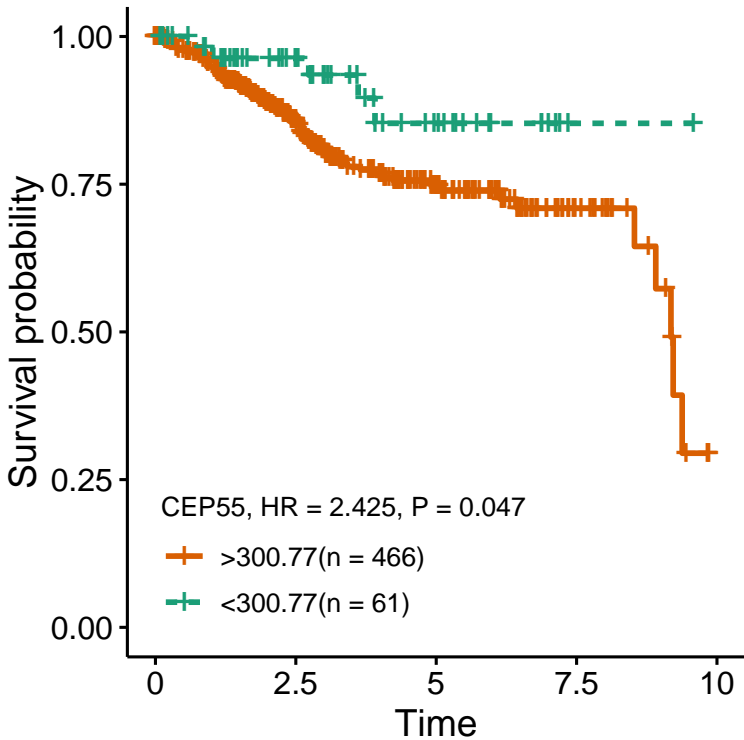

Supplement: Table S11 [file peerj-06-6091-s011.zip › Table S11/good_CEP55.pdf]

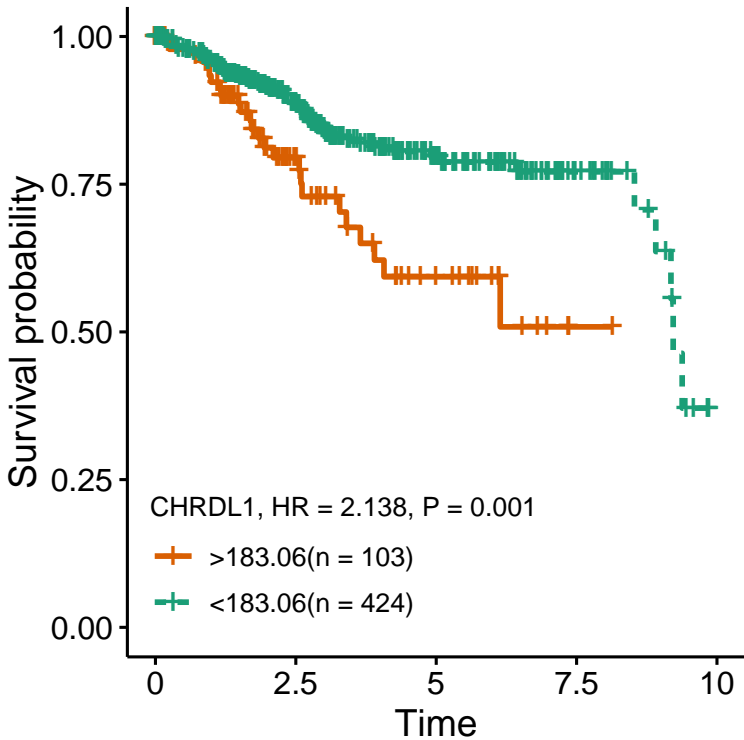

Supplement: Table S11 [file peerj-06-6091-s011.zip › Table S11/good_CHRDL1.pdf]

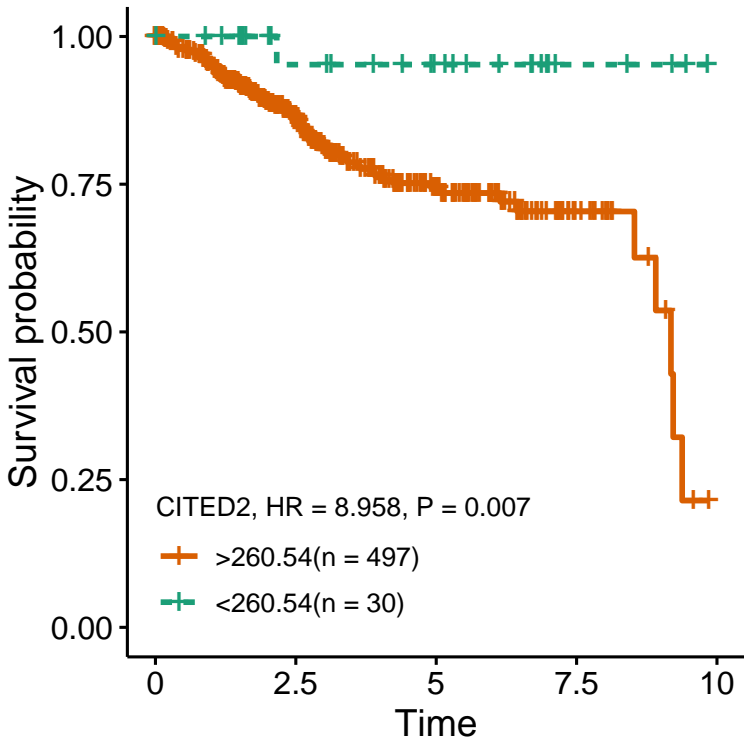

Supplement: Table S11 [file peerj-06-6091-s011.zip › Table S11/good_CITED2.pdf]

Survival probability

1.00  
0.75  
0.50  
0.25  
0.00

0

2.5

5

7.5

10

Time

CLIC4, HR = 2.025, P = 0.013

—+— >1794.01(n = 397)

—+— <1794.01(n = 130)

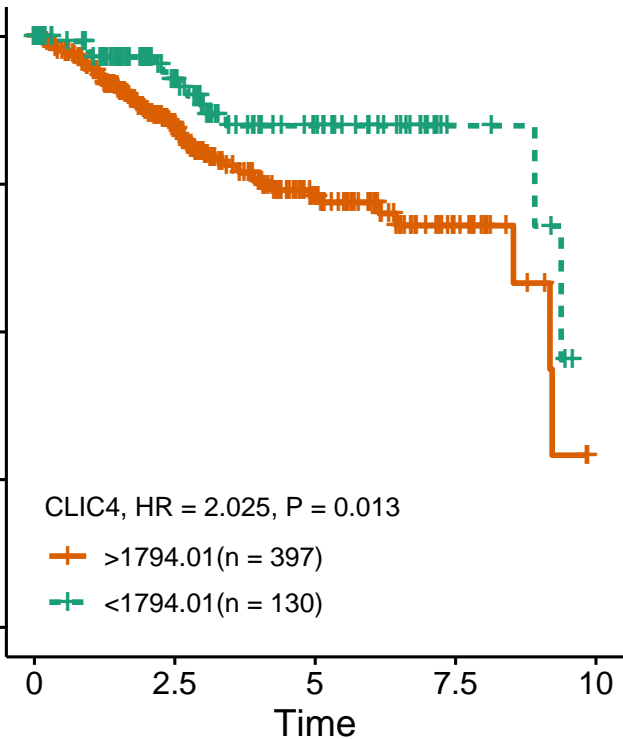

Supplement: Table S11 [file peerj-06-6091-s011.zip › Table S11/good_CLIC4.pdf]

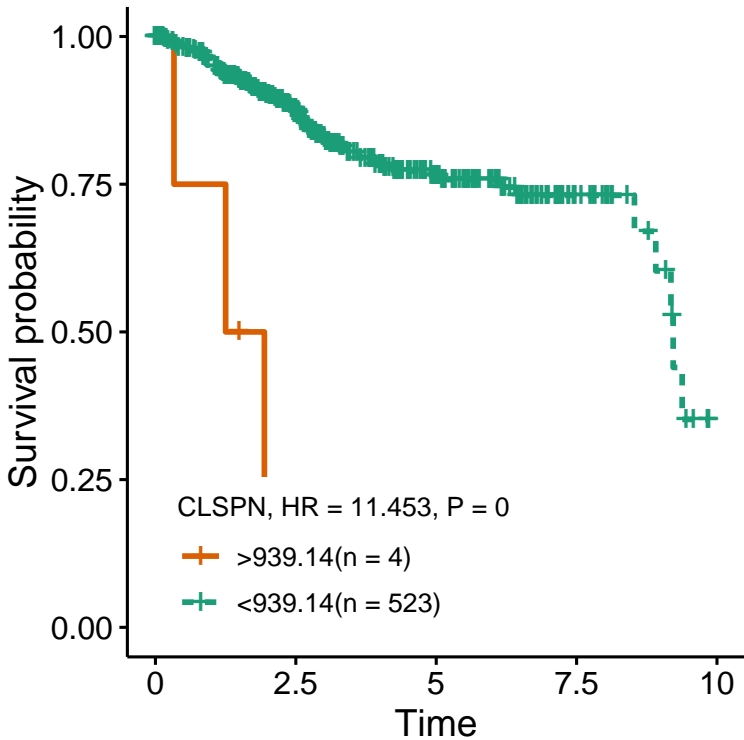

Supplement: Table S11 [file peerj-06-6091-s011.zip › Table S11/good_CLSPN.pdf]

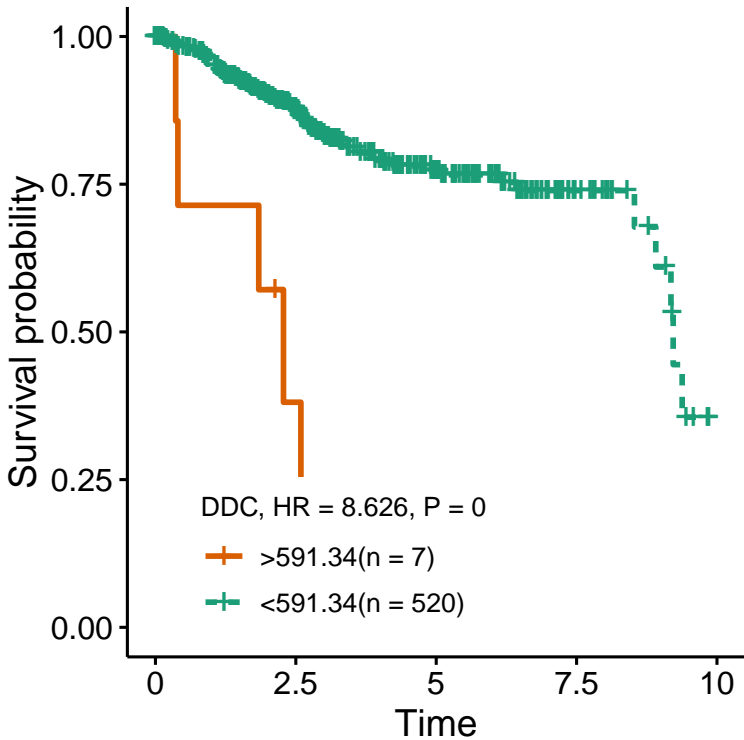

Supplement: Table S11 [file peerj-06-6091-s011.zip › Table S11/good_DDC.pdf]

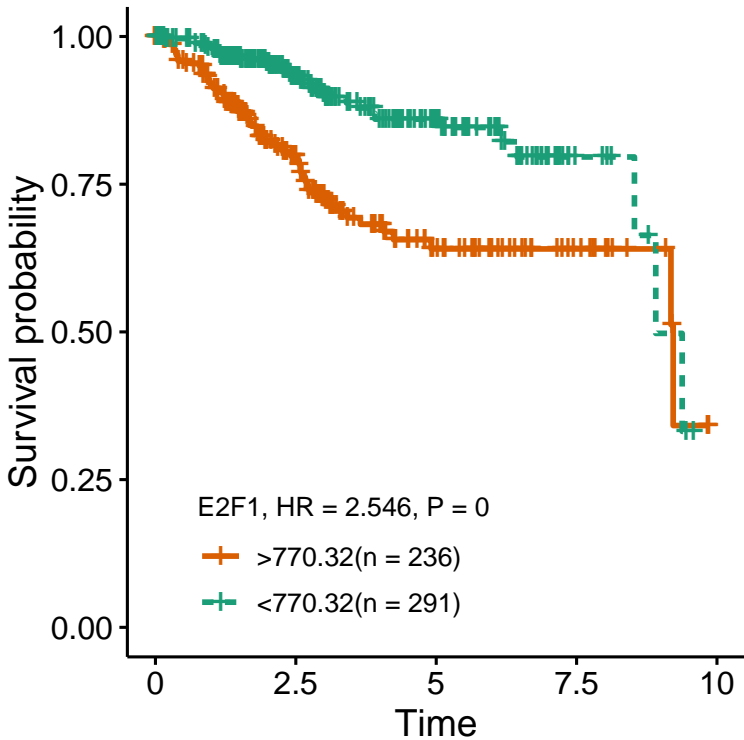

Supplement: Table S11 [file peerj-06-6091-s011.zip › Table S11/good_E2F1.pdf]

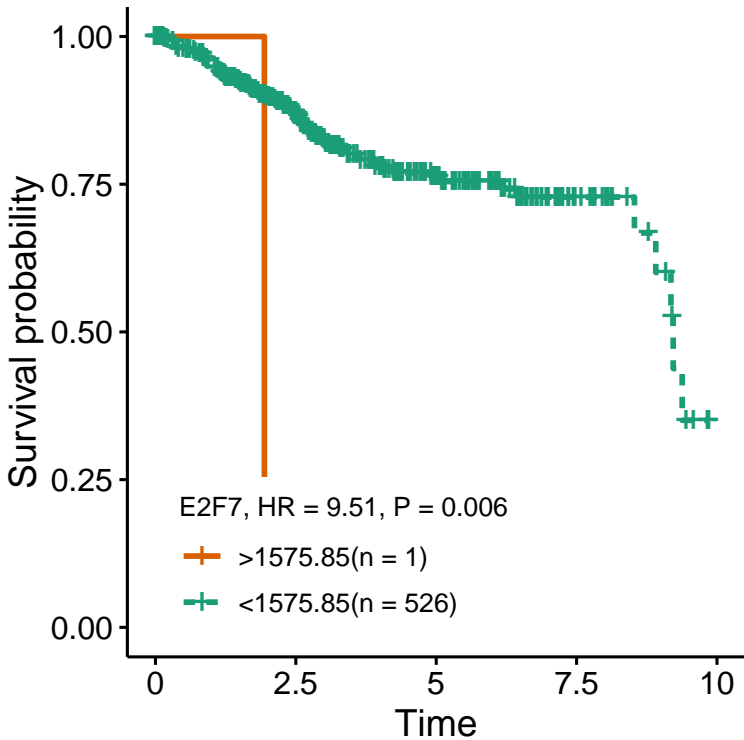

Supplement: Table S11 [file peerj-06-6091-s011.zip › Table S11/good_E2F7.pdf]

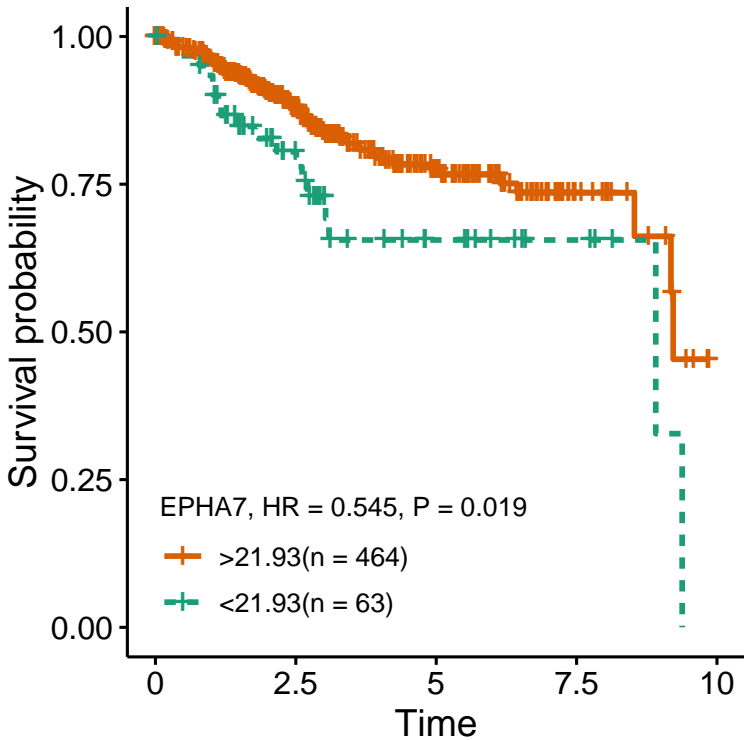

Supplement: Table S11 [file peerj-06-6091-s011.zip › Table S11/good_EPHA7.pdf]

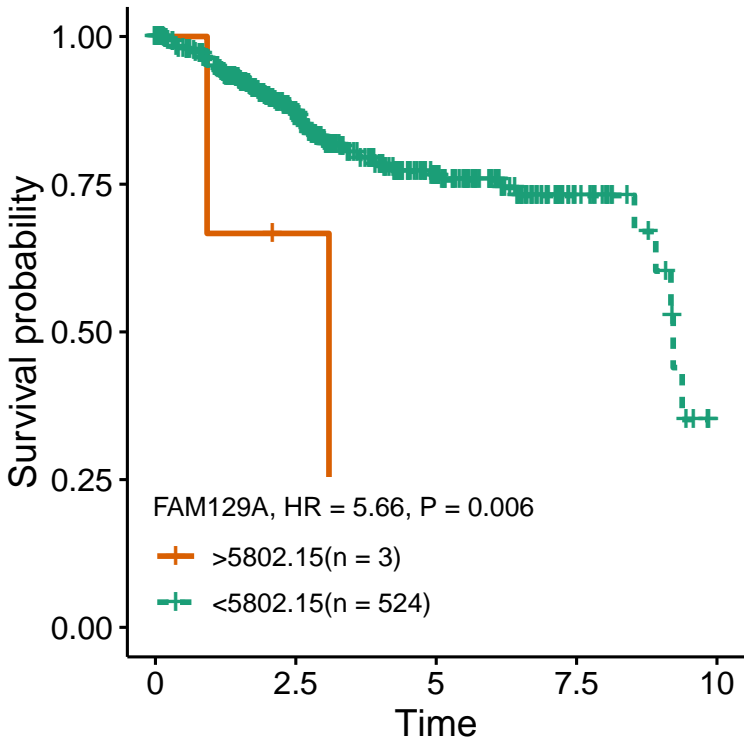

Supplement: Table S11 [file peerj-06-6091-s011.zip › Table S11/good_FAM129A.pdf]

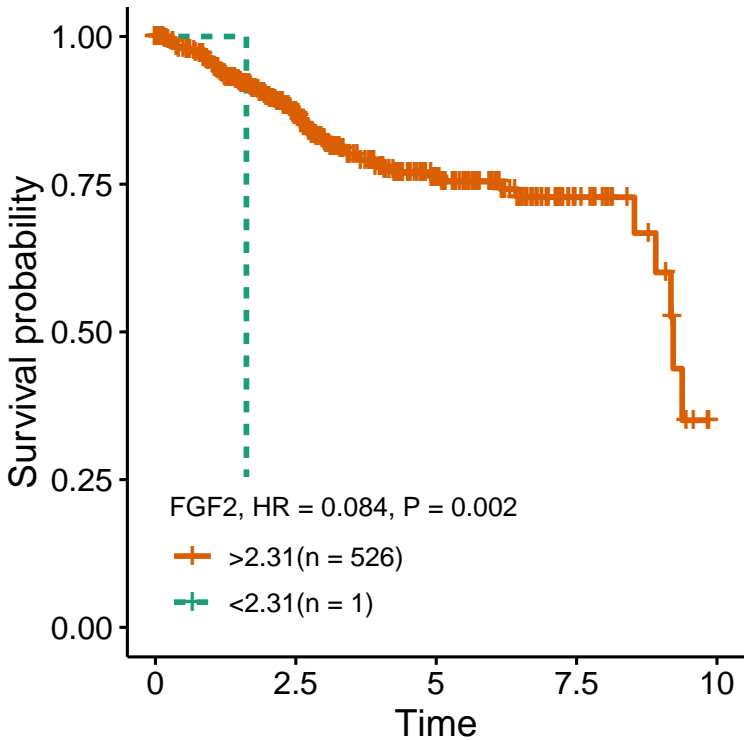

Supplement: Table S11 [file peerj-06-6091-s011.zip › Table S11/good_FGF2.pdf]

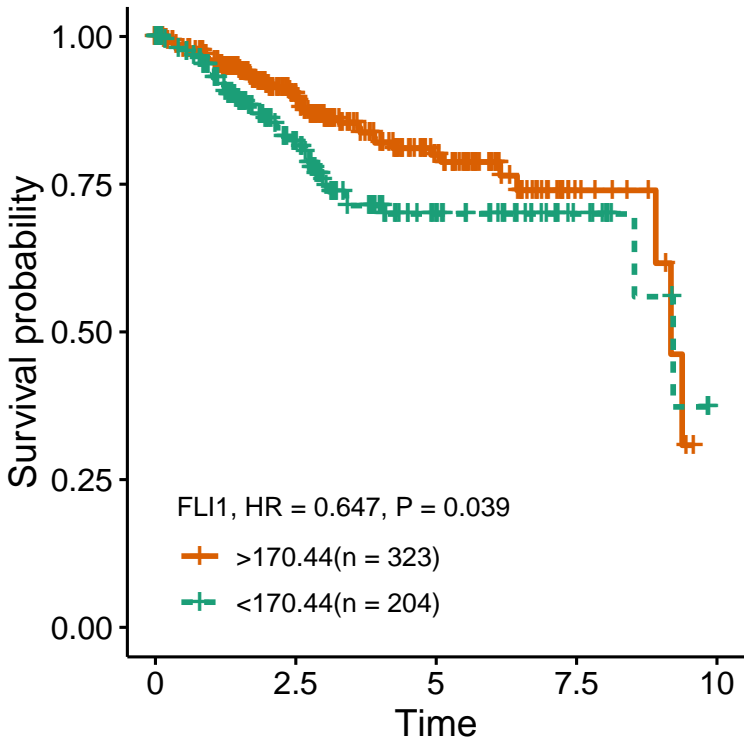

Supplement: Table S11 [file peerj-06-6091-s011.zip › Table S11/good_FLI1.pdf]

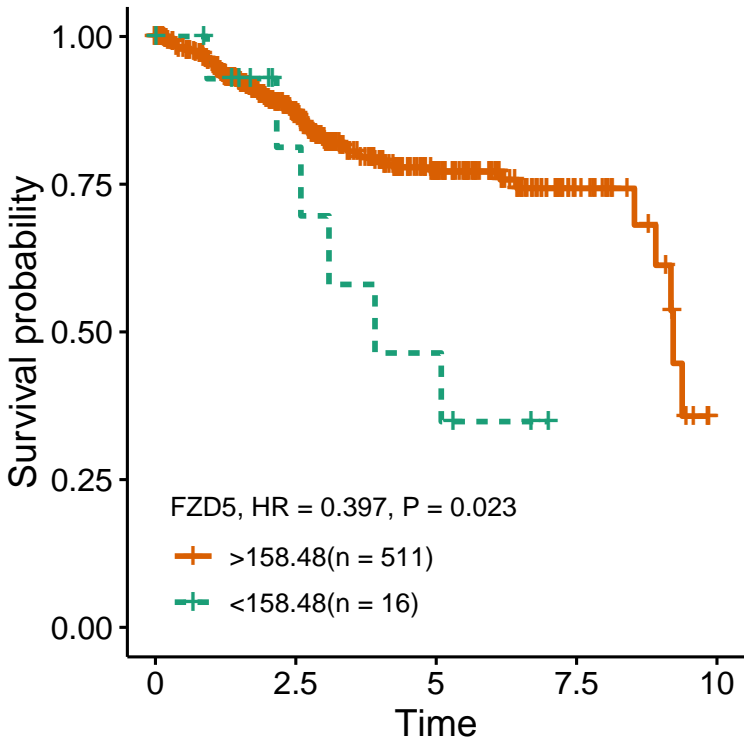

Supplement: Table S11 [file peerj-06-6091-s011.zip › Table S11/good_FZD5.pdf]

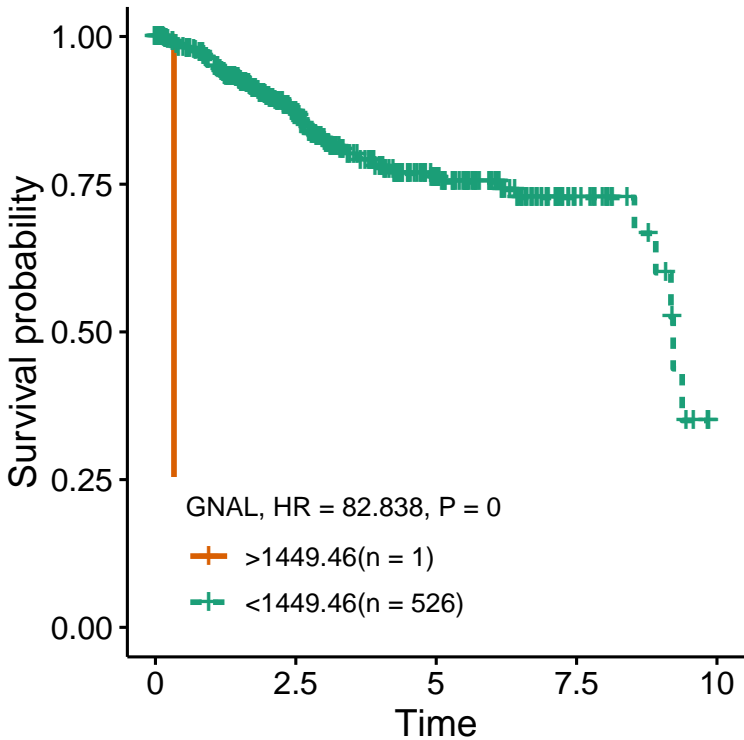

Supplement: Table S11 [file peerj-06-6091-s011.zip › Table S11/good_GNAL.pdf]

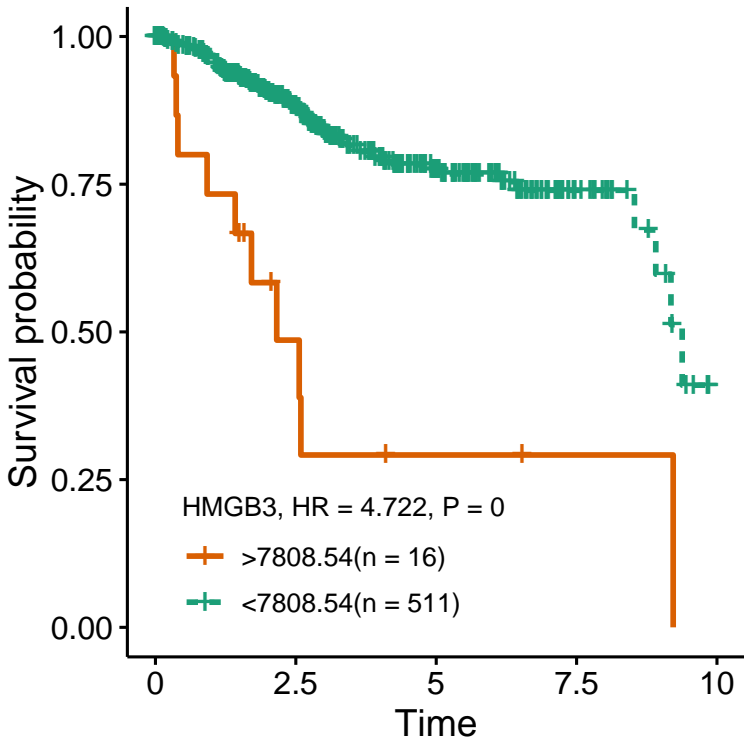

Supplement: Table S11 [file peerj-06-6091-s011.zip › Table S11/good_HMGB3.pdf]

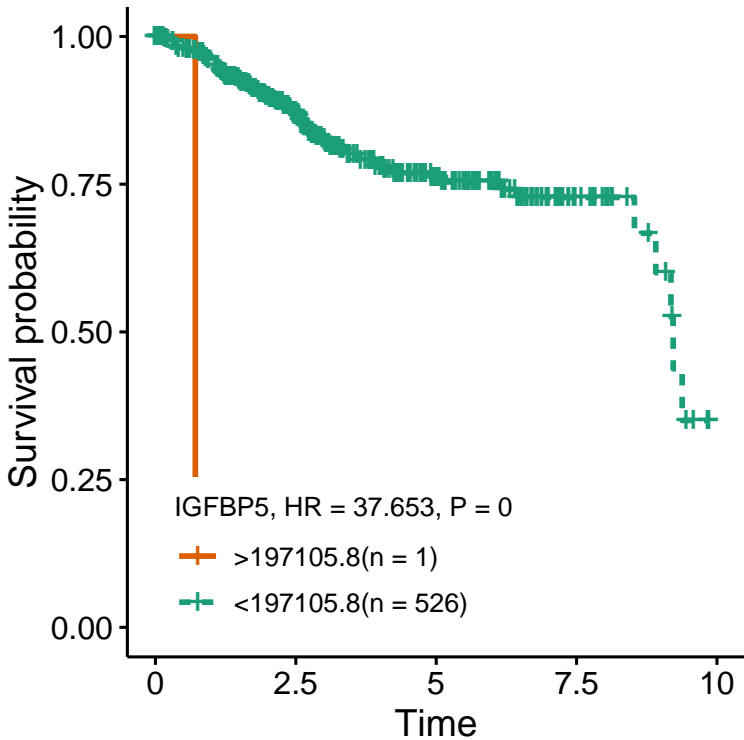

Supplement: Table S11 [file peerj-06-6091-s011.zip › Table S11/good_IGFBP5.pdf]

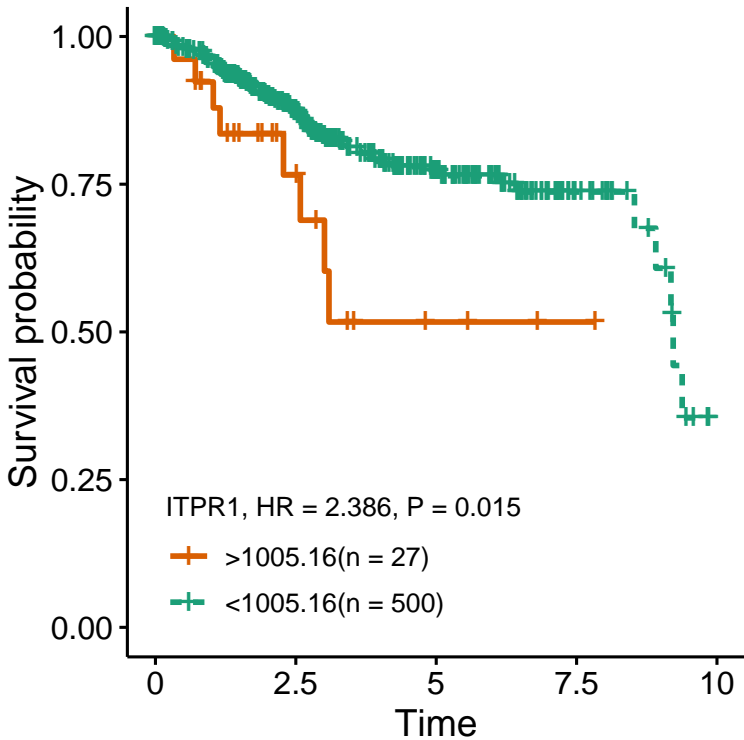

Supplement: Table S11 [file peerj-06-6091-s011.zip › Table S11/good_ITPR1.pdf]

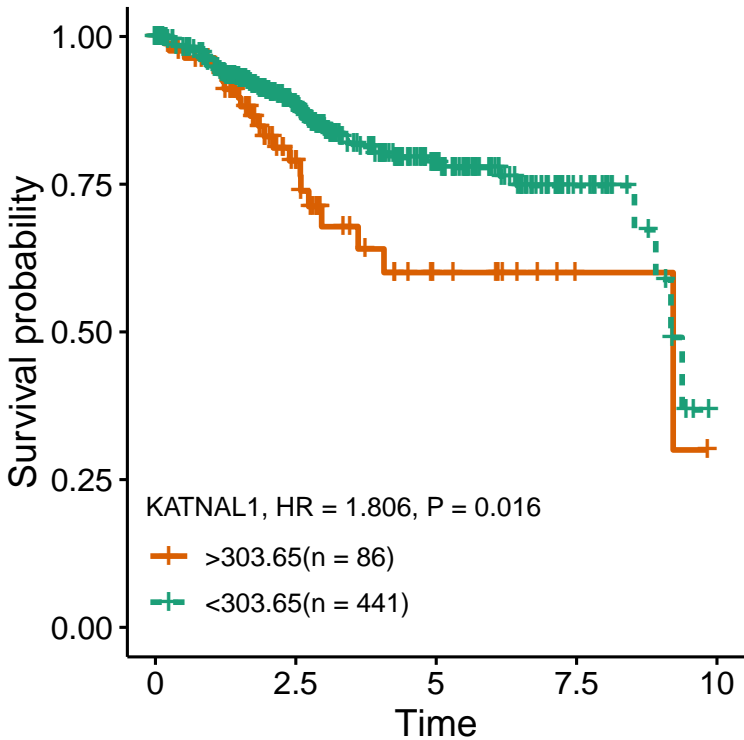

Supplement: Table S11 [file peerj-06-6091-s011.zip › Table S11/good_KATNAL1.pdf]

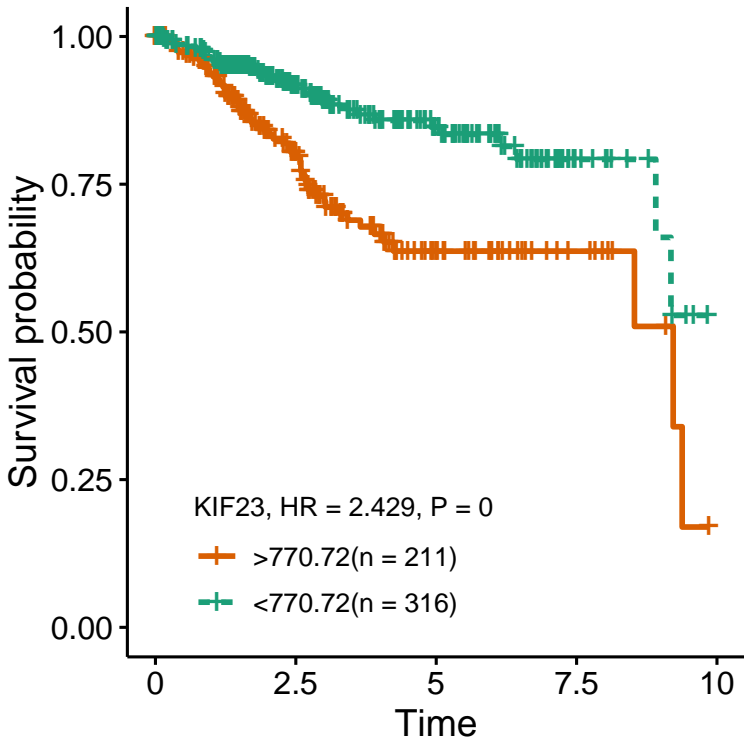

Supplement: Table S11 [file peerj-06-6091-s011.zip › Table S11/good_KIF23.pdf]

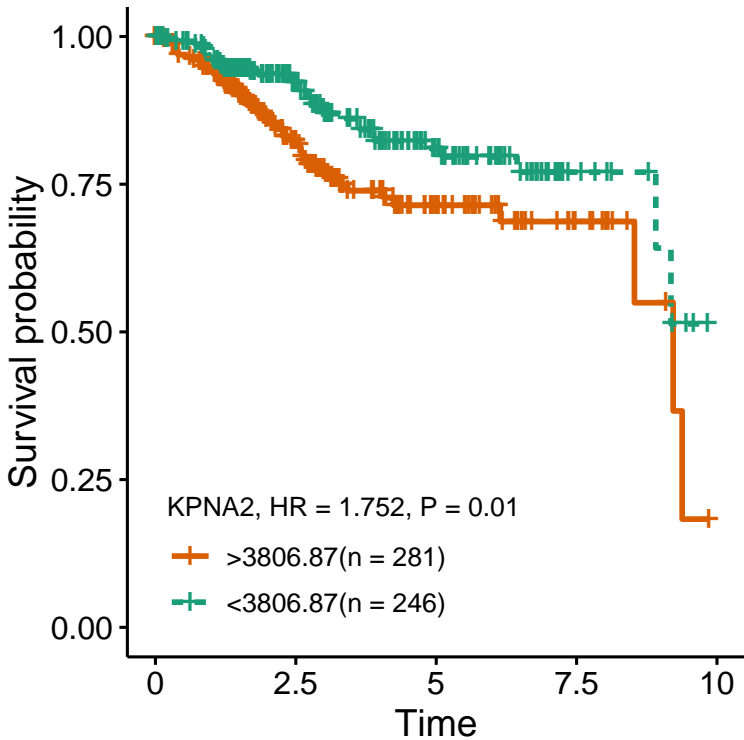

Supplement: Table S11 [file peerj-06-6091-s011.zip › Table S11/good_KPNA2.pdf]

Survival probability

1.00  
0.75  
0.50  
0.25  
0.00

0

2.5

5

7.5

10

Time

LRRK2, HR = 5.395, P = 0.008

+ >12.35(n = 475)

+ <12.35(n = 52)

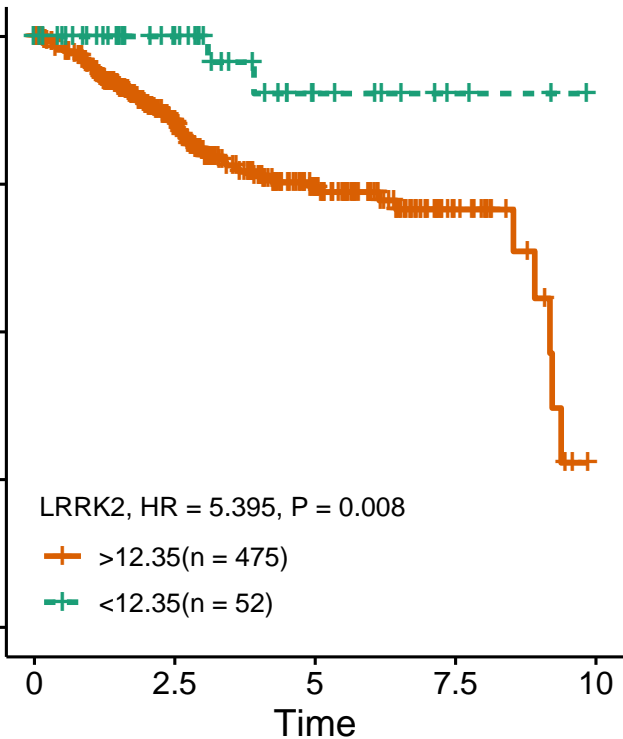

Supplement: Table S11 [file peerj-06-6091-s011.zip › Table S11/good_LRRK2.pdf]

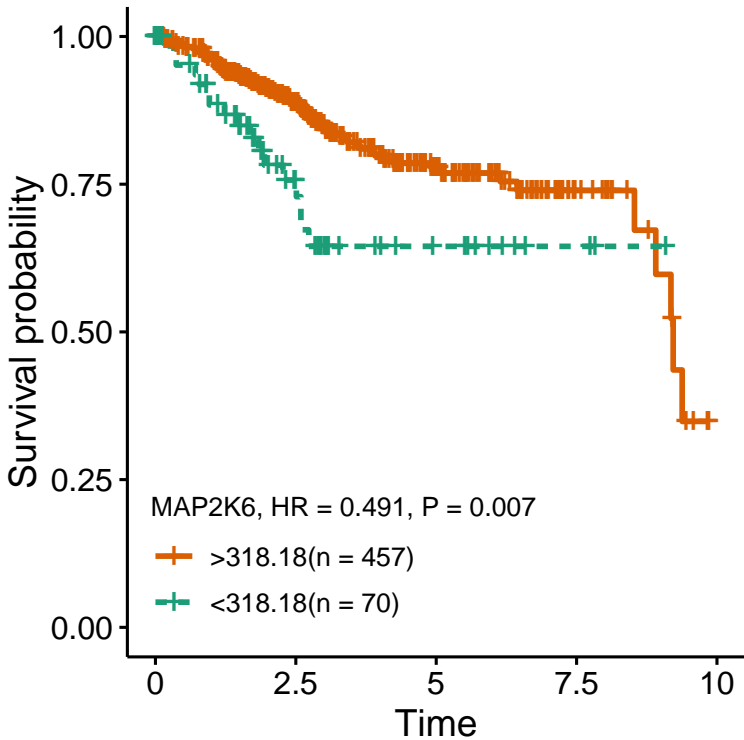

Supplement: Table S11 [file peerj-06-6091-s011.zip › Table S11/good_MAP2K6.pdf]

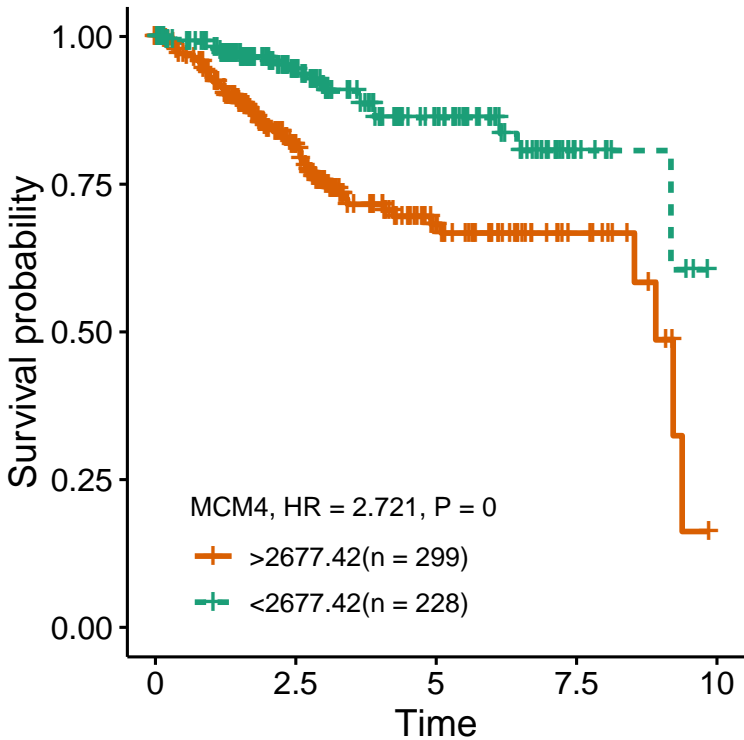

Supplement: Table S11 [file peerj-06-6091-s011.zip › Table S11/good_MCM4.pdf]

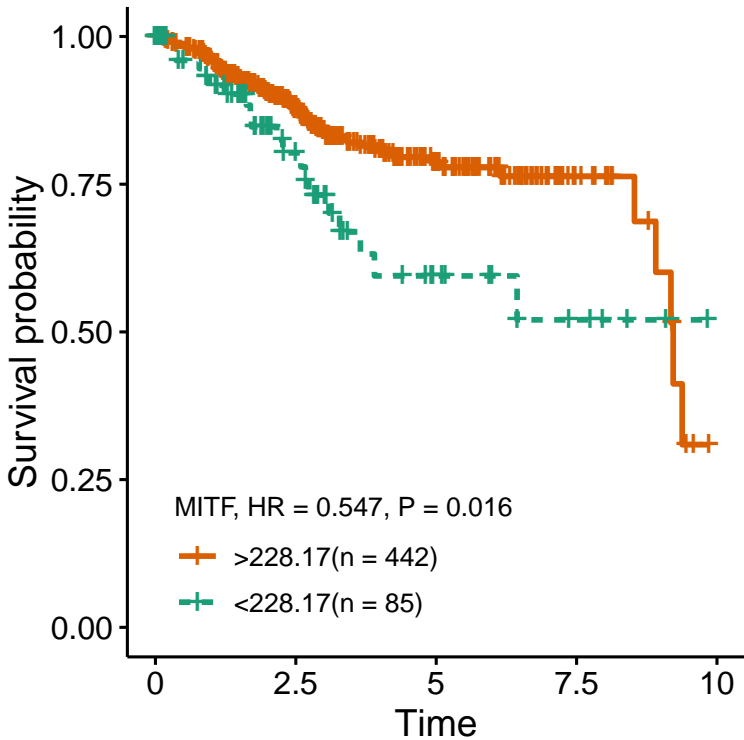

Supplement: Table S11 [file peerj-06-6091-s011.zip › Table S11/good_MITF.pdf]

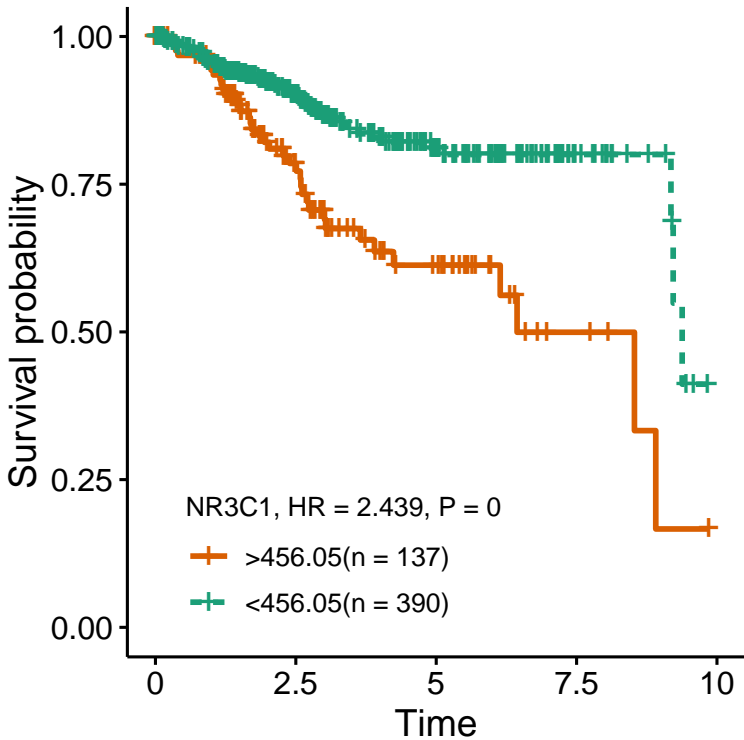

Supplement: Table S11 [file peerj-06-6091-s011.zip › Table S11/good_NR3C1.pdf]

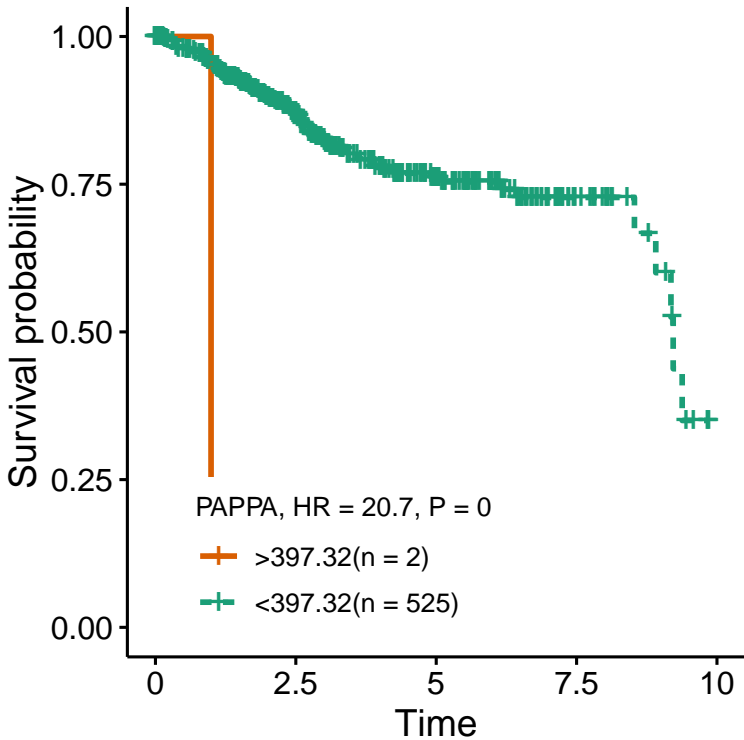

Supplement: Table S11 [file peerj-06-6091-s011.zip › Table S11/good_PAPPA.pdf]

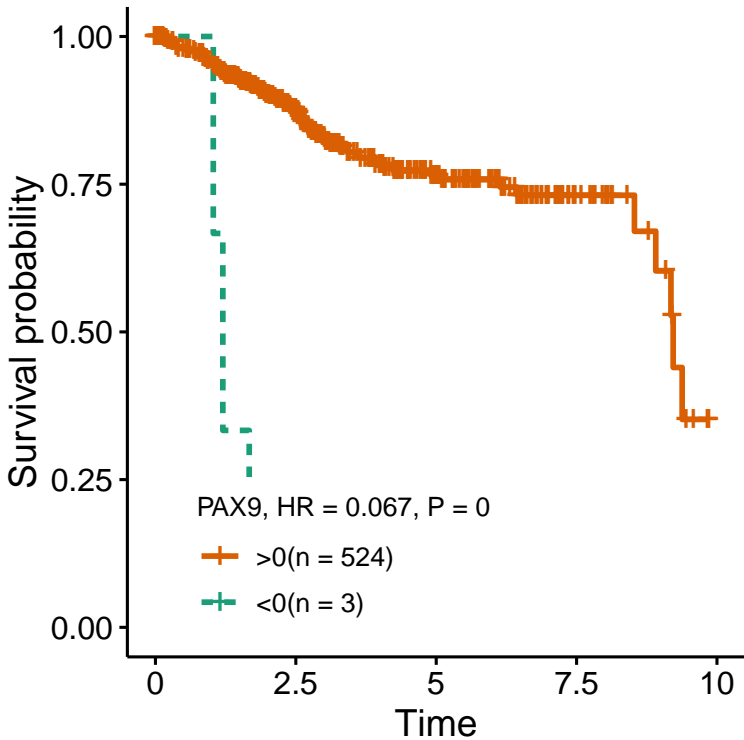

Supplement: Table S11 [file peerj-06-6091-s011.zip › Table S11/good_PAX9.pdf]

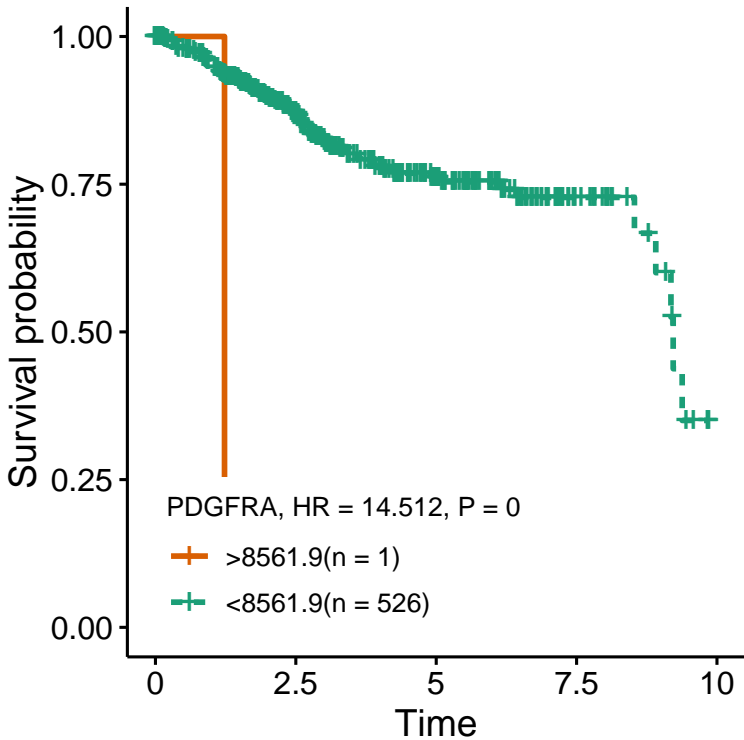

Supplement: Table S11 [file peerj-06-6091-s011.zip › Table S11/good_PDGFRA.pdf]

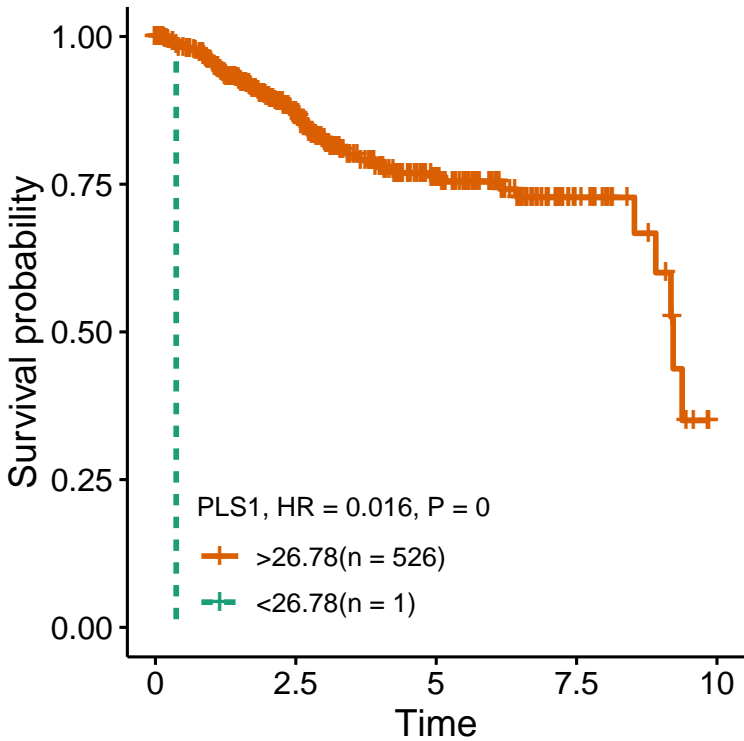

Supplement: Table S11 [file peerj-06-6091-s011.zip › Table S11/good_PLS1.pdf]

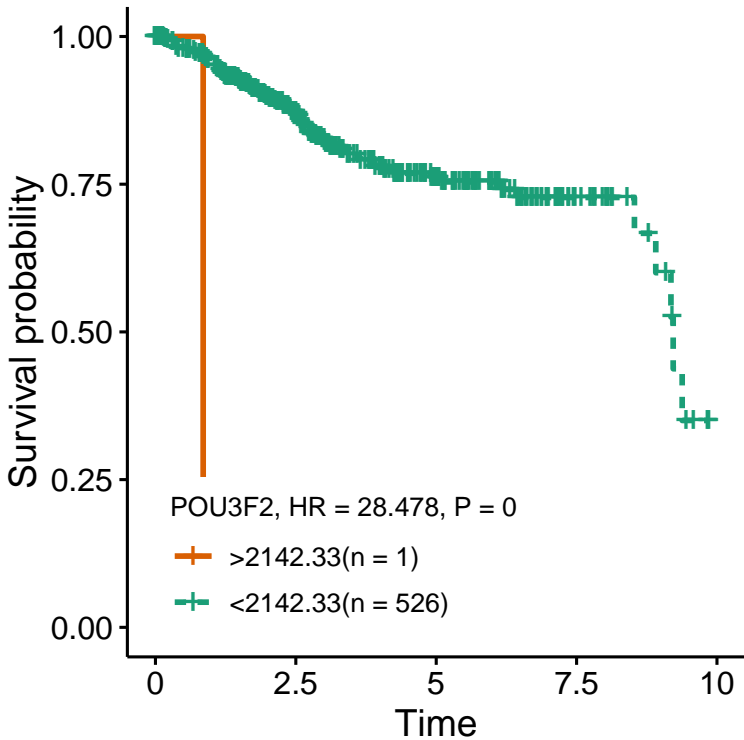

Supplement: Table S11 [file peerj-06-6091-s011.zip › Table S11/good_POU3F2.pdf]

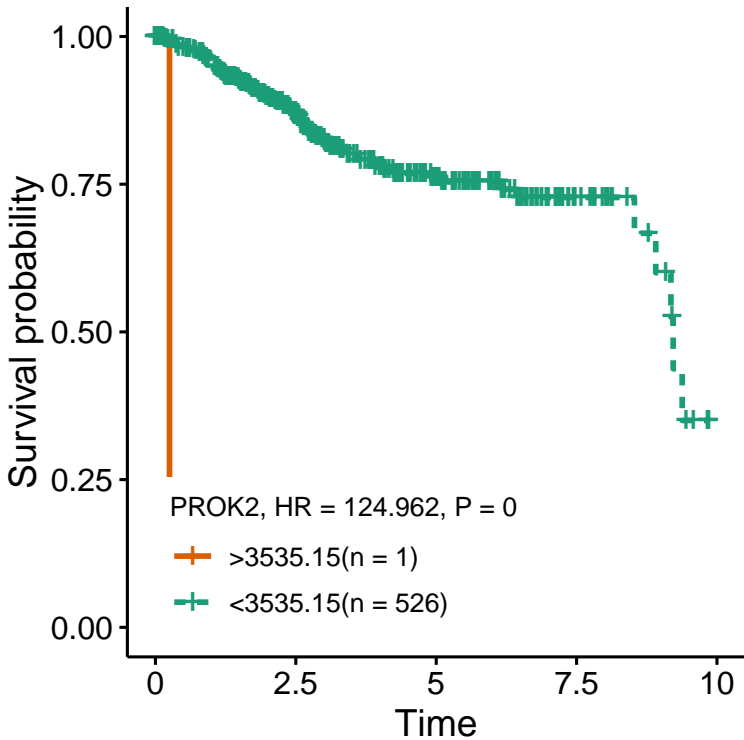

Supplement: Table S11 [file peerj-06-6091-s011.zip › Table S11/good_PROK2.pdf]

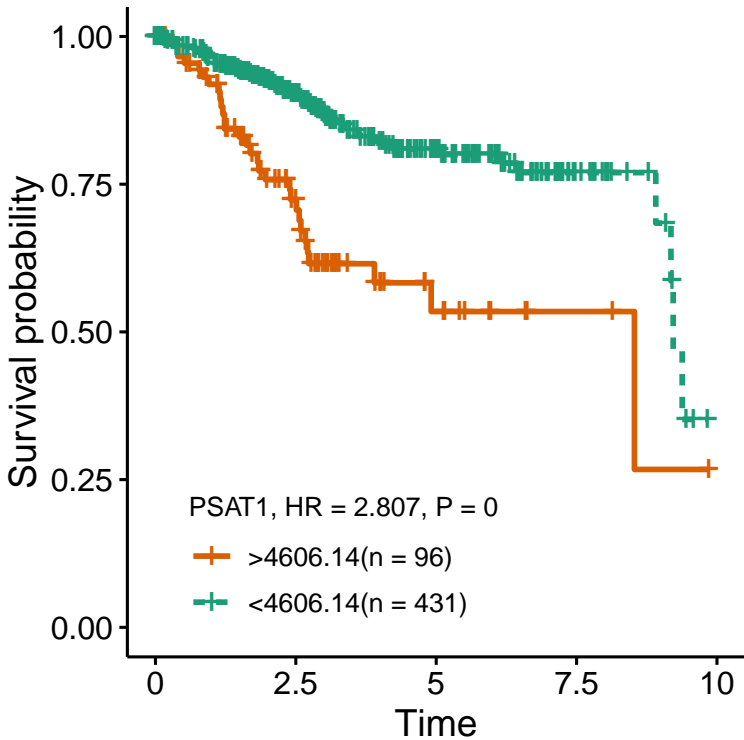

Supplement: Table S11 [file peerj-06-6091-s011.zip › Table S11/good_PSAT1.pdf]

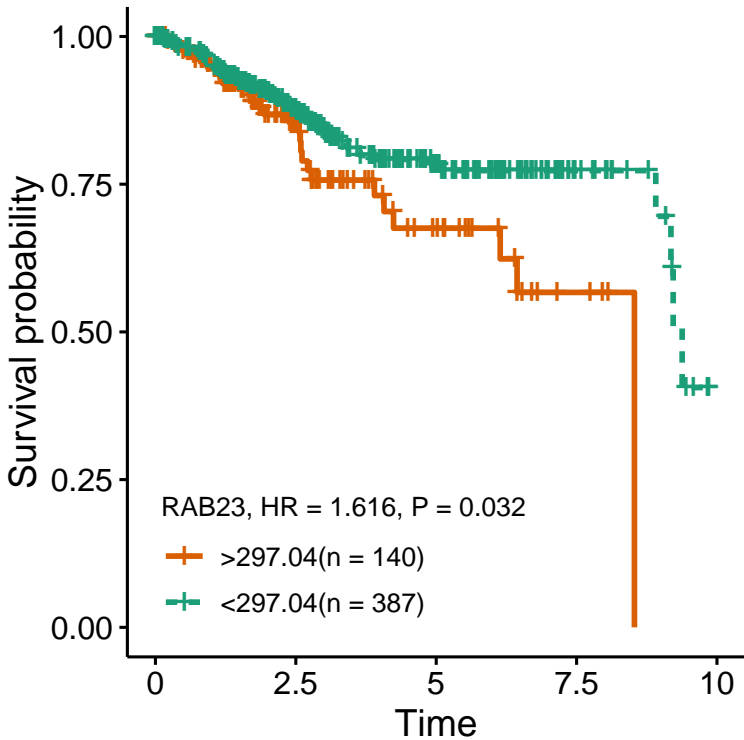

Supplement: Table S11 [file peerj-06-6091-s011.zip › Table S11/good_RAB23.pdf]

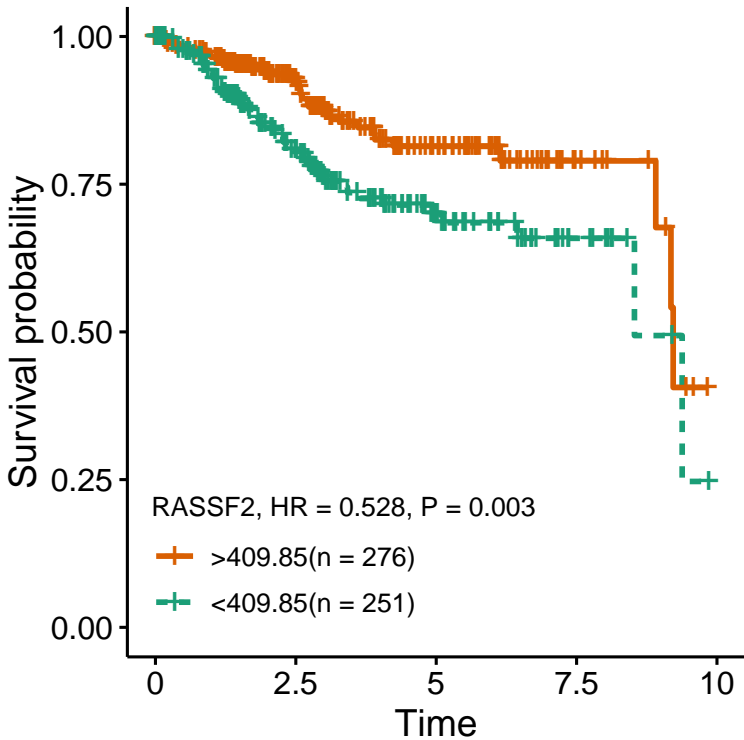

Supplement: Table S11 [file peerj-06-6091-s011.zip › Table S11/good_RASSF2.pdf]

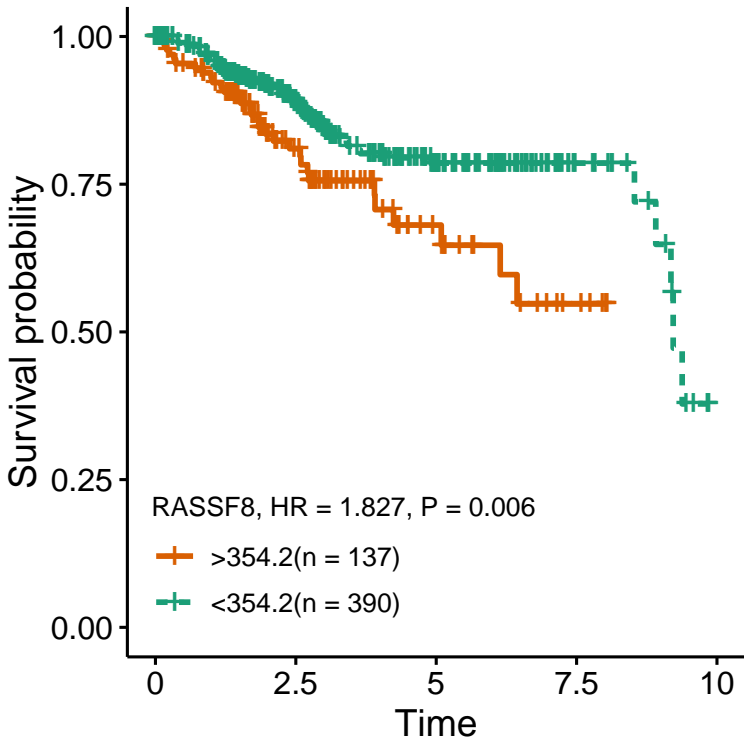

Supplement: Table S11 [file peerj-06-6091-s011.zip › Table S11/good_RASSF8.pdf]

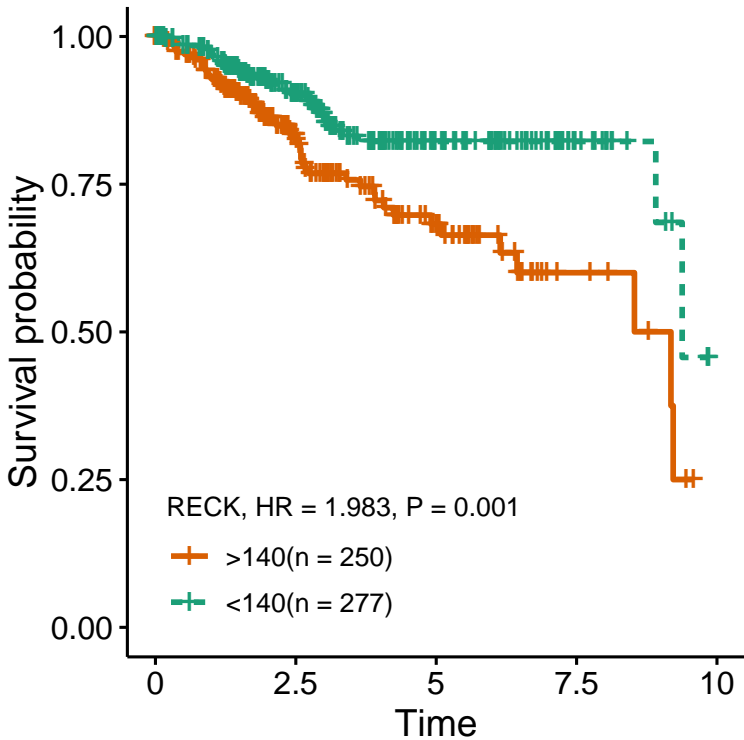

Supplement: Table S11 [file peerj-06-6091-s011.zip › Table S11/good_RECK.pdf]

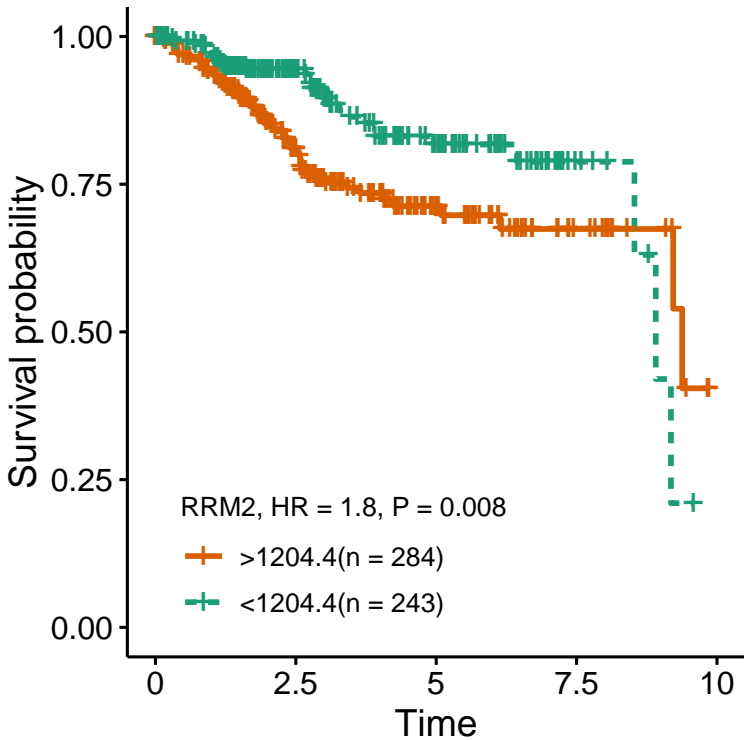

Supplement: Table S11 [file peerj-06-6091-s011.zip › Table S11/good_RRM2.pdf]

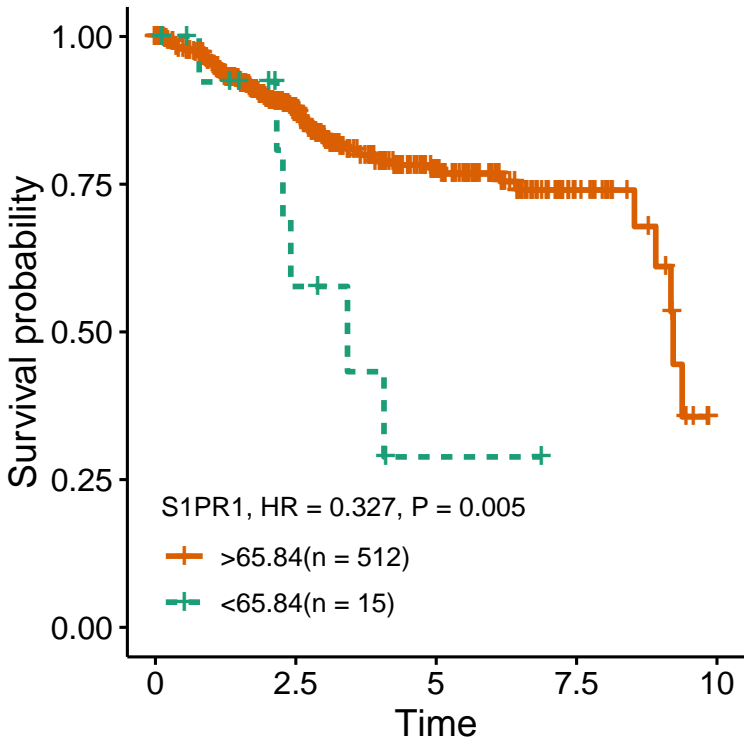

Supplement: Table S11 [file peerj-06-6091-s011.zip › Table S11/good_S1PR1.pdf]

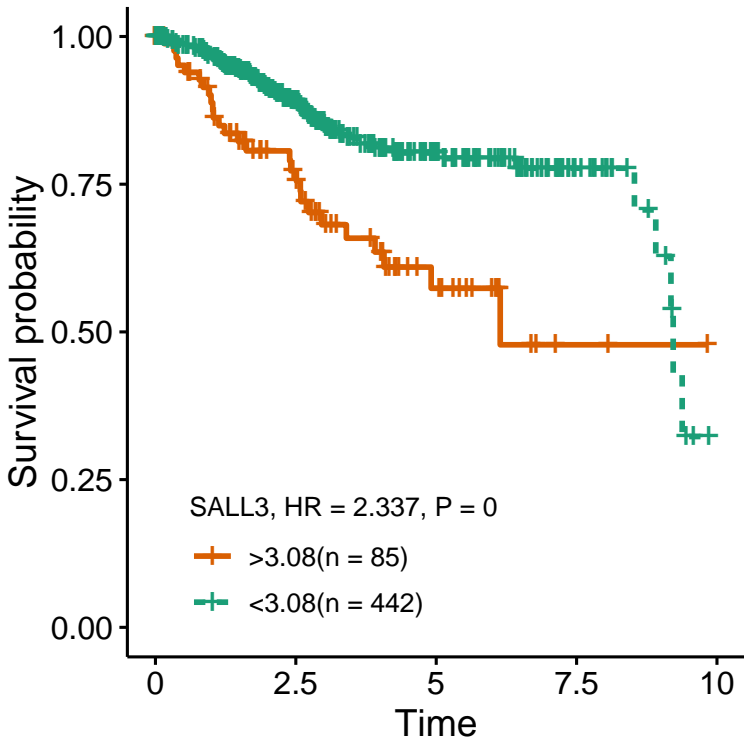

Supplement: Table S11 [file peerj-06-6091-s011.zip › Table S11/good_SALL3.pdf]

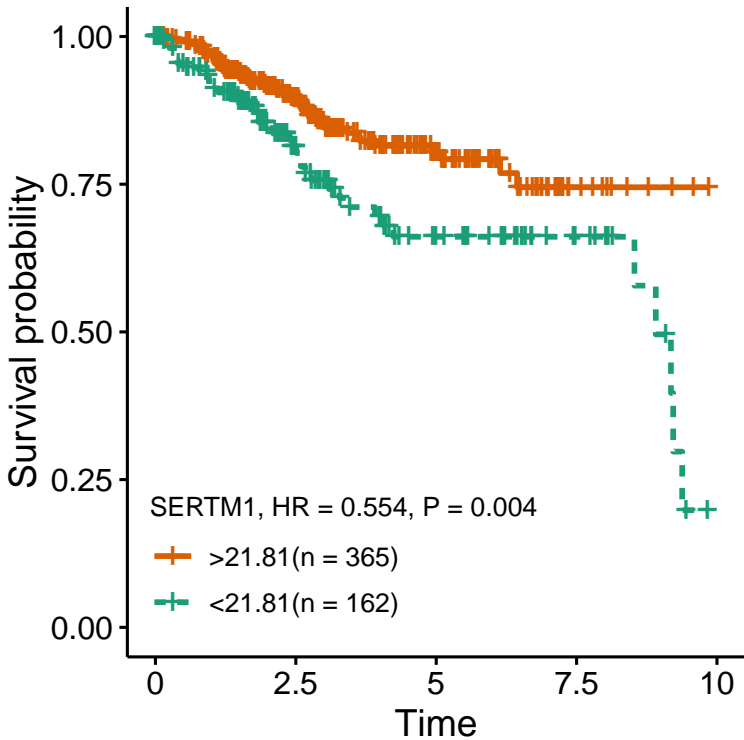

Supplement: Table S11 [file peerj-06-6091-s011.zip › Table S11/good_SERTM1.pdf]

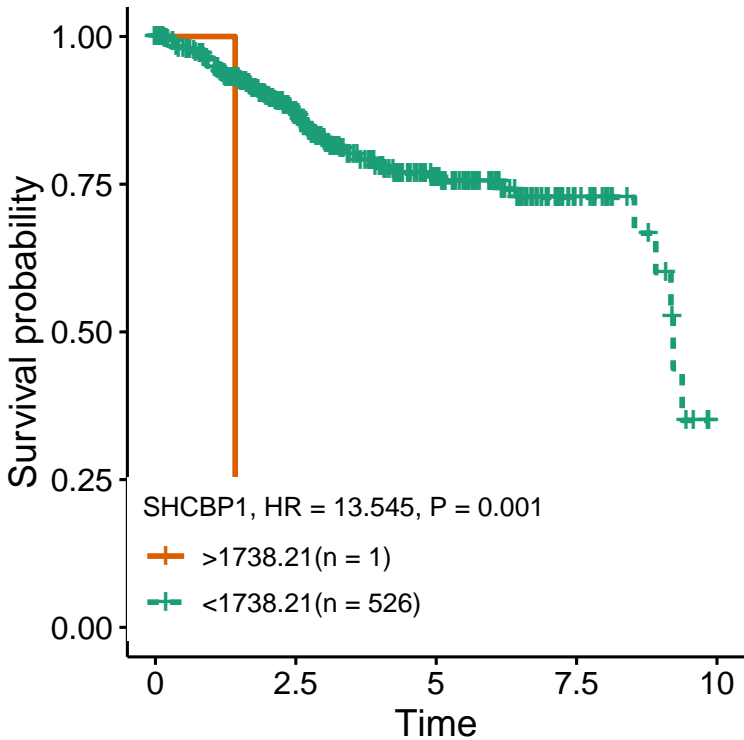

Supplement: Table S11 [file peerj-06-6091-s011.zip › Table S11/good_SHCBP1.pdf]

Survival probability

1.00  
0.75  
0.50  
0.25  
0.00

0

2.5

5

7.5

10

Time

SLC12A5, HR = 2.613, P = 0.002

—+— >363.7(n = 32)

—+— <363.7(n = 495)

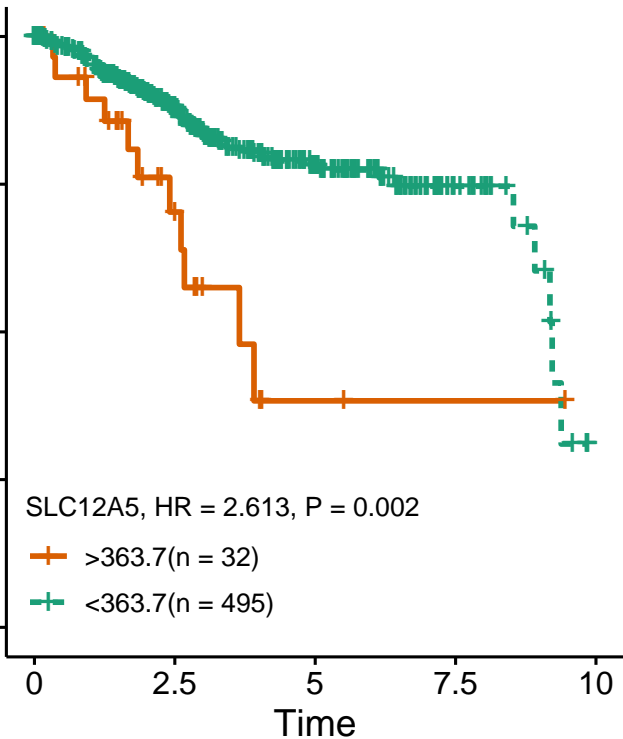

Supplement: Table S11 [file peerj-06-6091-s011.zip › Table S11/good_SLC12A5.pdf]

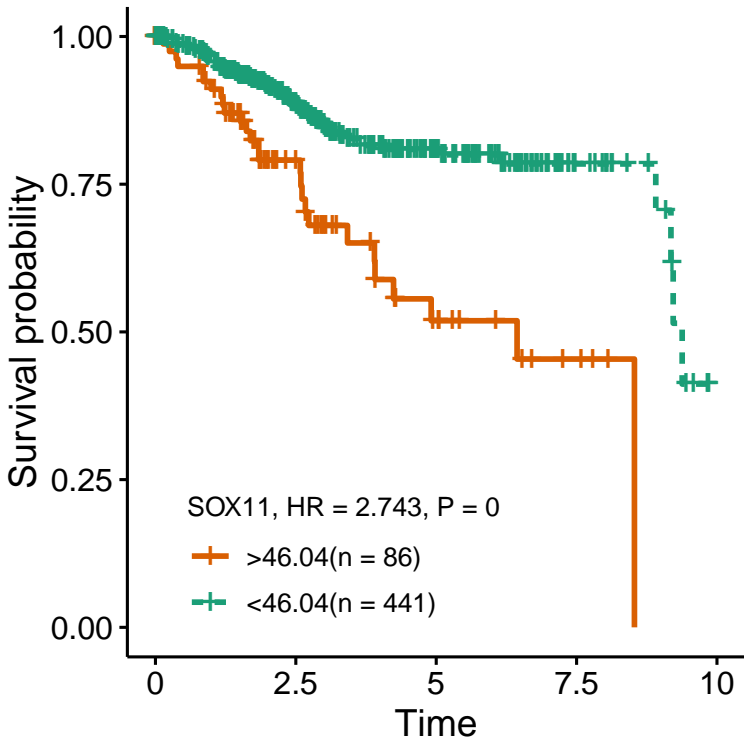

Supplement: Table S11 [file peerj-06-6091-s011.zip › Table S11/good_SOX11.pdf]

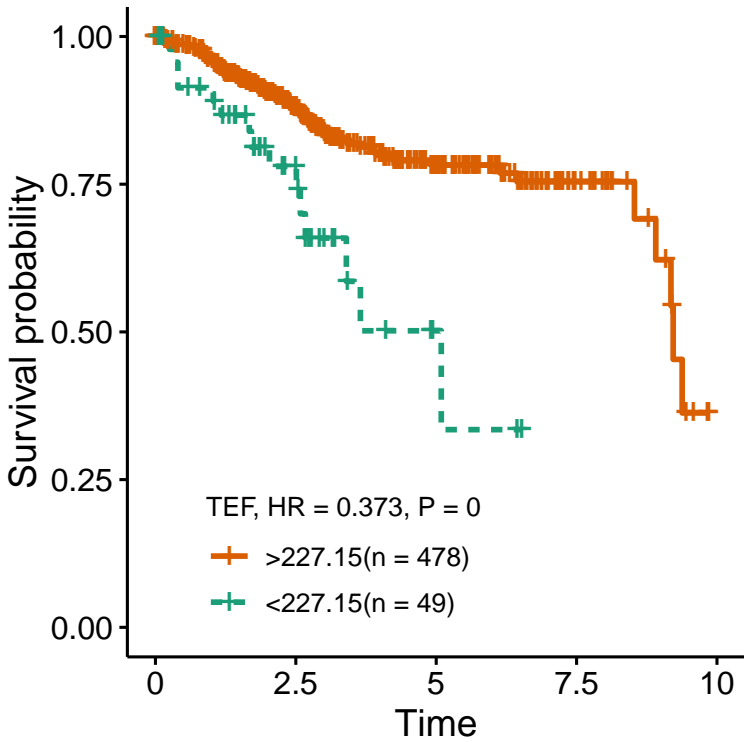

Supplement: Table S11 [file peerj-06-6091-s011.zip › Table S11/good_TEF.pdf]

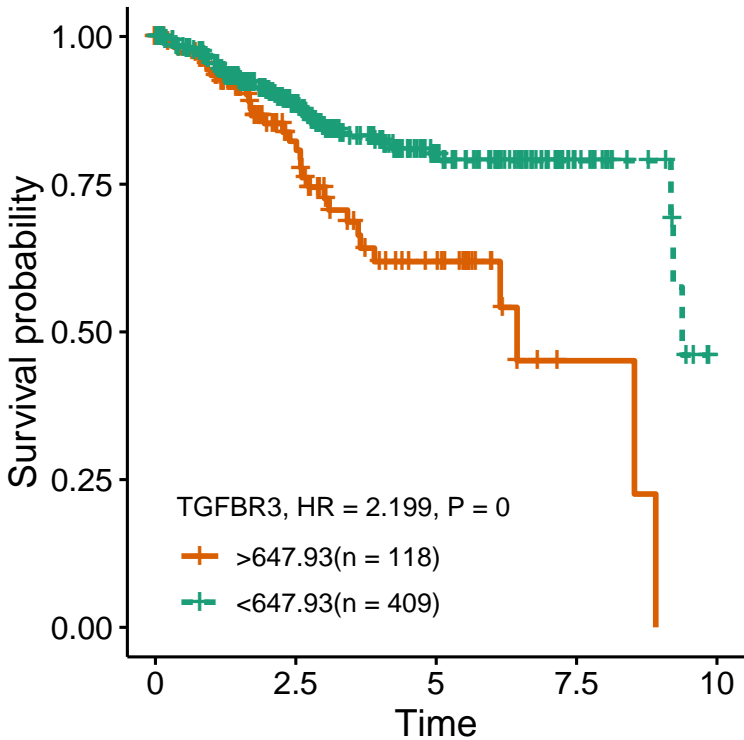

Supplement: Table S11 [file peerj-06-6091-s011.zip › Table S11/good_TGFBR3.pdf]

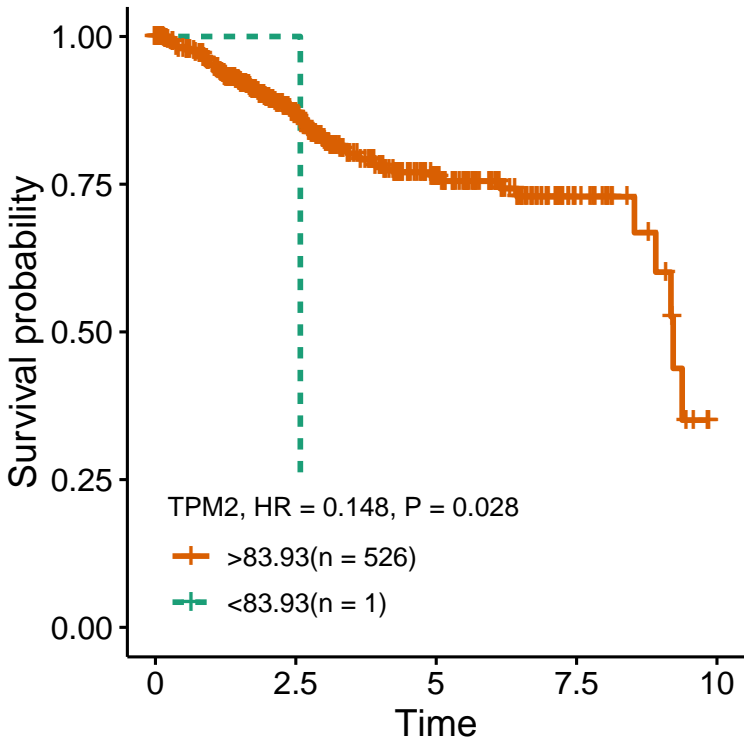

Supplement: Table S11 [file peerj-06-6091-s011.zip › Table S11/good_TPM2.pdf]

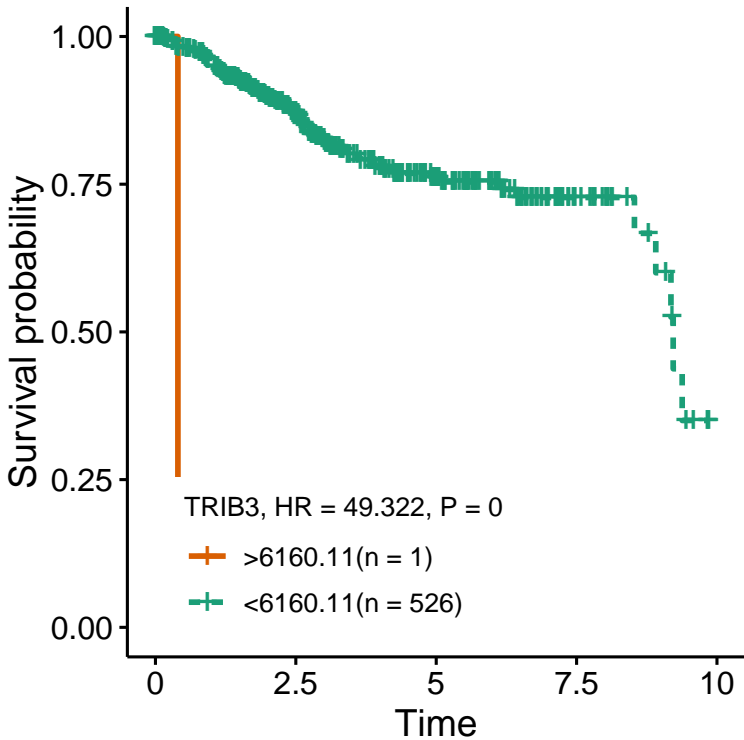

Supplement: Table S11 [file peerj-06-6091-s011.zip › Table S11/good_TRIB3.pdf]

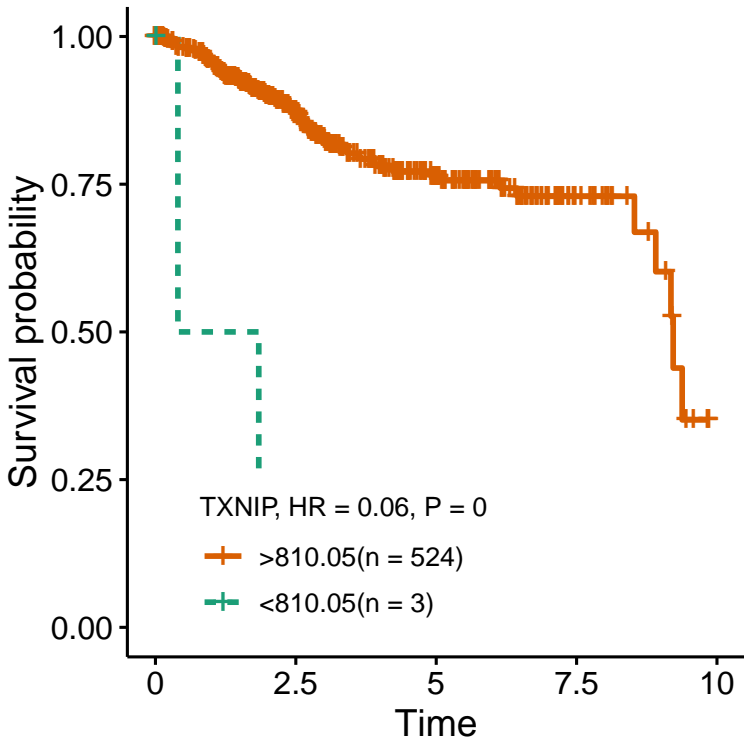

Supplement: Table S11 [file peerj-06-6091-s011.zip › Table S11/good_TXNIP.pdf]

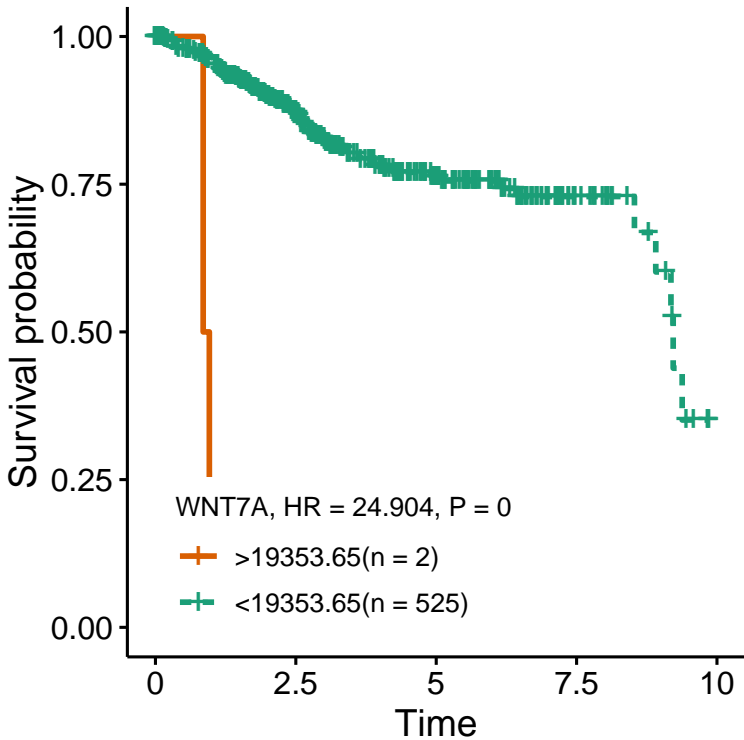

Supplement: Table S11 [file peerj-06-6091-s011.zip › Table S11/good_WNT7A.pdf]

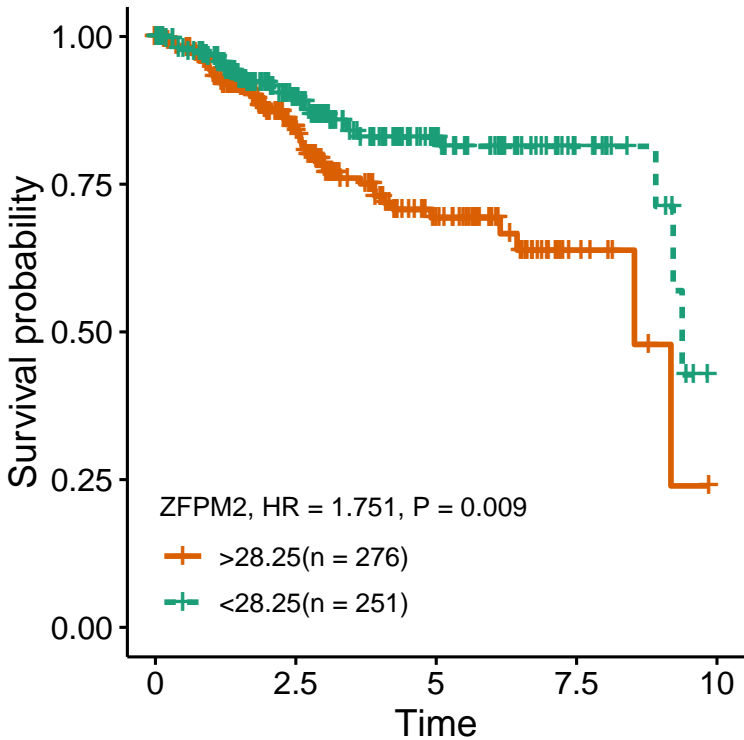

Supplement: Table S11 [file peerj-06-6091-s011.zip › Table S11/good_ZFPM2.pdf]
